# KPNA2 promotes renal cell carcinoma proliferation and metastasis via NPM

**DOI:** 10.1111/jcmm.16846

**Published:** 2021-09-01

**Authors:** Song Zheng, Xiaofan Li, Ting Deng, Rong Liu, Junjie Bai, Teng Zuo, Yinan Guo, Jianhui Chen

**Affiliations:** ^1^ Department of Urology Fujian Medical University Union Hospital Fuzhou China; ^2^ Department of Hematology Fujian Institute of Hematology Union Hospital Fujian Medical University Fuzhou China; ^3^ Fujian Provincial Key Laboratory on Hematology Fujian Medical University Fuzhou China; ^4^ Department of Gynecology Fujian Maternity and Child Health Hospital Fuzhou China

**Keywords:** kidney renal clear cell carcinoma, KPNA2, NPM, proliferation, tumorigenesis

## Abstract

Karyopherin α2 (KPNA2), involved in nucleocytoplasmic transport, has been reported to be up‐regulated in tumorigenesis. However, comprehensive studies of KPNA2 functions in renal cell carcinoma (RCC) are still lacking. In this study, we aim to investigate the roles of KPNA2 in kidney tumour development. Our results showed that down‐regulation of KPNA2 inhibited the proliferation and invasion of kidney tumour cell cells in *vitro*, while the cell cycle arrest and cellular apoptosis were induced once KPNA2 was silenced. Repression of KPNA2 was proved to be efficient to repress tumorigenesis and development of kidney tumour in in nude mice. Furthermore, one related participator, NPM, was identified based on Co‐IP/MS and bioinformatics analyses. The up‐regulation of NPM attenuates the efficiency of knockdown KPNA2. These results indicated that KPNA2 may regulate NPM to play a crucial role for kidney tumour development.

## 1 INTRODUCTION

Renal cell carcinoma (RCC) is one the most common cancers worldwide, accounting for 2% of global cancer deaths and more than 90% of cancer diagnoses in the kidney ^1^. More than 14,770 deaths were estimated in the United States at 2019 ^2^. Three major subtypes, clear cell RCC ^3^, papillary RCC ^4^ and chromophobe RCC ^5^, and a rare subtypes ^6^ unable to fit any subtype diagnostic criteria, were present in clinical patients ^7^. Among these subtypes of RCC, clear cell RCC (ccRCC), also known as kidney renal clear cell carcinoma (KIRC), is the most common one, causing approximately 13,000 deaths in China at 2014 ^8^. As one of the well‐known cancers with resistant to radiotherapy and chemotherapy ^1^, ccRCC causes less than 20% 2‐year survival rate of patients, which requires well‐defined diagnostic biomarkers to facilitate early detection and risk stratification. Surgery is the primary treatment choice for localized renal cancer. Localized RCC can be treated with partial or radical nephrectomy ablation or active surveillance ^9^, ^10^, ^11^, yet up to 30% of these patients develop metastases eventually nonetheless ^12^. There are currently no efficient immunotherapy and molecular targeted drugs because of the overt complexity of intratumour and intertumour heterogeneity for clinical outcomes observed ^4^, ^13^, ^14^, ^15^. Therefore, it is imperative to reveal the underlying molecular mechanisms and to identify molecular biomarkers of ccRCC for therapeutic targets.

Nuclear transporters play a crucial role in regulating the pathological development of cancers. Karyopherin α2 (KPNA2, also known as importin α1) belongs to 7 members of the karyopherin α (also called importin α) protein family, which plays a major role in nucleocytoplasmic transport ^16^, ^17^. KPNA2 is essential for proper nuclear localization and multiple functions of NBS1, and the interaction between KPNA2 and NBS1 is crucial for the DNA repair role process ^18^, ^19^. The abnormal expression of KPNA2 exhibited a considerably changed activity in DSB repair, which is proved critical for the DNA damage‐activated cell cycle checkpoint. Apart from this, KPNA2 has also been identified to mediate nuclear transport of tumour suppressors ^17^, ^20^. Recent studies have demonstrated the association between the up‐regulation of KPNA2 and various types of malignancies ^21^, ^22^, ^23^, ^24^. These results suggested the effect exerted by KPNA2 in tumour formation and progression is through cell differentiation, proliferation and apoptosis. Although accumulating evidence confirmed the participating of KPNA2 in the progression of tumour, little is known about the oncogene effects of KPNA2 in ccRCC, as well as its potential effective function for suppression of ccRCC development.

As a well‐known nucleolar phosphoprotein, NPM1 has been found to be strongly correlated with cell proliferation and cancer pathogenesis. This gene has oncogenic and tumour‐suppressing functions through the frequent overexpression or genetic modification ^25^, ^26^, ^27^, ^28^. The mutation of NPM1 has been proved to be related to leukaemia and lymphoma ^29^, ^30^. NPM1 is overexpressed in a variety of human solid tumours including tumours of colon ^31^, ovary ^32^and prostate and other tumours ^33^, ^34^. The specific contribution of overexpressed NPM1 to cancer development is not fully understood, but it may arise from multiple factors. Inhibition of NPM1 effectively suppressed the viability of tumour cell lines ^35^, ^36^, ^37^, making it as one of the hopeful targets for cancer treatment ^28^.

In this study, we found the expression of KPNA2 was significantly increased in the late stage compared with the early stage in ccRCC patients. Based on the clinical information, the higher expression of KPNA2 was remarkably correlated with the survival rate of patients. Further analysis based on siRNA (small interfering RNA) of KPNA2 demonstrated the key role of KPNA2 in kidney tumour cell proliferation, migration and apoptosis, which grants it one of the most promising biomarkers and therapeutic targets for early diagnosis of kidney tumour. Silencing of KPNA2 significantly repressed the development of tumour cells in *vivo*. Furthermore, NPM1 was identified as a cooperative protein for KPNA2 in tumorigenesis process. Meanwhile, increasing the expression of NPM1 could attenuate the repression effect of silencing KPNA2 in tumour cells. The relationship between KPNA2 and NPM1 would provide a new therapeutic strategy for ccRCC.

## 2 MATERIALS AND METHODS

### 2.1 Cell culture

Human renal clear cell carcinoma cell lines, Caki−1, 786‐O and ACHN, were obtained from the Cell Repository of Shanghai Institute of Life Sciences, Chinese Academy of Sciences (Shanghai, China). 786‐O cells were cultured in RPMI 1640 (Gibco, USA) supplemented with 10% (v/v) foetal bovine serum (Gibco, USA). ACHN cells were maintained in Dulbecco's modified Eagle's medium (DMEM; HyClone Laboratories, Logan, UT, USA) supplemented with 10% foetal bovine serum (FBS, Gibco), 100 µg/ml streptomycin and 100 µg/ml penicillin. Caki‐1 cells were cultured in RPMI 1640 medium with 10% (v/v) foetal calf serum (FCS) and 100 U/ml penicillin. All cells were incubated in an incubator at 37℃ with a humidified atmosphere of 5% CO_2_. When the cells in the culture flask reached 80% confluence, the culture solution was discarded and washed once, and 2.5g/L trypsin was added at a dilution of 1: 5.

### 2.2 shRNA design and lentivirus preparation

Short‐hairpin RNA (shRNA) specifically targeting *KPNA2*, 5’‐ACCTCTGAAGGCTACACTT‐3’, was designed based on human *KPNA2* gene (NM_002266), and the DNA oligonucleotides of target sequence were synthesized by Shanghai GeneRay Biotech Co., Ltd. KPNA2 siRNAs were inserted into the GV610 lentivirus core vector containing an enhanced green fluorescent protein (Genechem, Shanghai, China; Figure S1) using the Fermentas T4 DNA Ligase (Fermentas, Burlington, Canada) according to the manufacturer's instructions. The positive clones were sequenced using the identified primers, and clones that are consistent with the target sequence were selected. Lentiviral vector without siRNA insertion was used as a negative control.

### 2.3 Cell transfection

To investigate the role of KPNA2 in human renal clear cell carcinoma cell, 786‐O and ACHN were infected with lentiviruses expressing either shCtrl or shKPNA2. In brief, cells were seeded into 6‐, 12‐ or 24‐well plates, and when the cells grew up to about 30%, the lentiviral vector was added to the cells. The cultures were abandoned, and normal culture medium was added 12 hours later. When the positive rate of GFP was up to 70%‐80% and the cell density was about 80%, the cells were collected and used in the following experiments. The percentage of cells that was GFP‐positive cells represented the lentiviral infection efficiency, which was examined using a fluorescence microscope.

The full‐length coding sequence of the human KPNA2 was cloned into the lentiviral vector GV610 lentivirus core vector with C‐terminal 3 × FLAG tag (Jikai, Shanghai, China). The KPNA2‐encoding virus was then transfected into ACHN cell line. The full‐length coding sequence of the human NPM1 was cloned into the lentiviral vector GV610 lentivirus core vector containing EGFP. Cells were cultured in 96‐well plates. And when the cells grow up to about 30%, lentiviral vectors with target sequence were added to the cells. The cultures were abandoned, and normal culture medium was added 12 hours later. The expression of green fluorescent protein (GFP) was observed under fluorescence microscope. When the positive rate of GFP was up to 70%‐80% and the cell density was about 80%, the cells were collected and used in the following experiments. The primers used were as follows: KPNA2, 5’‐TGTGGTAGATGGAGGTGC‐3’ (forward) and reverse, 5’‐GAGCCAACAGTGGGTCA’ (reverse). The experiments were repeated three times.

### 2.4 Western blotting for protein expression analysis

Total cellular proteins were harvested and separated by SDS‐PAGE gels and then transferred onto PVDF membranes (Millipore). Equal protein loading was verified by the Bradford assay. The primary antibodies used in Western blot were the same as those in ‘Immunocytochemistry’. Membranes were blocked for 30 min at room temperature with 5% non‐fat dry milk in TBS containing 0.05% Tween‐20 (TBST), probed with the primary antibodies at a 1:1000 dilution for 1 h at room temperature and washed four times with TBST. The secondary anti‐mouse/rabbit IgG‐HRP (DAKO) was added for 1 h at room temperature. After extensive rinsing, immunoreactive protein bands were visualized with the ECL detection system (Santa Cruz, USA) and subsequently exposed to film. Densitometric assay was performed using Quantity one software (Bio‐Rad, USA). The specific primary antibodies were purchased from the following resource: anti‐FLAG (Sigma), anti‐MCM3 (Cell Signaling Technology, California, USA), anti‐NPM1 (Sigma), anti‐RAN (Cell Signaling Technology, California, USA), anti‐YBX1 (Abcam, Cambridge, UK), anti‐GAPDH (Santa Cruz Biotechnology, Dallas, TX, USA) and anti‐KPNA2 (Abcam, Cambridge, UK).

### 2.5 RNA extraction and Quantitative Real‐Time PCR (RT‐qPCR)

The total RNA was extracted using TRIzol reagent (Shanghai Pufei, Shanghai, China) according to the manufacturer's instructions. The RNA concentration was determined with the NanoDrop 2000/2000C spectrophotometer (cat. no. 2000/2000C; NanoDrop Technologies; Thermo Fisher Scientific, Inc.). Reverse transcription was performed with the Promega M‐MLV RT (Promega, Durham, NM, USA) for cDNA first‐strand synthesis. 1 µl oligo‐dT (0.5 µg/µl; cat. no. B0205; Sangon, Shanghai, China) and 2.0 µg total RNA were used. After incubation at 70℃ for 10 min, the mixture was then placed in an ice bath immediately for 2 min. M‐MLV RT 5× reaction buffers, M‐MLV Reverse Transcriptase RNase Minus and 10 mM dNTP (cat.no. U1240; Promega) were added to the mixture and then incubated at 42℃ for 1 h, followed by 70℃ for 10 min. cDNA was stored at −80℃. The mRNA of KPNA2 was examined by using SYBR Master Mixture (TAKARA, DRR041B, Dalian, China), and RT‐qPCR was carried out with SYBR Master Mixture (DRR041B; Takara, Dalian, China) on the MX3000p real‐time PCR system (Agilent, Utrecht, the Netherlands). For PCR amplification, glyceraldehyde‐phosphate dehydrogenase (GAPDH) was employed as the internal reference gene. The results were recorded when the cycle was finished. Relative gene expression was calculated with the 2^−ΔΔCt^ method. The primers used were as follows: KPNA2, 5’‐TGTGGTAGATGGAGGTGC‐3’ (forward) and reverse, 5’‐GAGCCAACAGTGGGTCA‐3’ (reverse); and GAPDH, forward, 5ʹ‐TGACTTCAACAGCGACACCCA‐3ʹ (forward) and reverse, 5ʹ‐CACCCTGTTGCTGTAGCCAAA‐3ʹ (reverse). The experiments were repeated three times.

### 2.6 Cell growth assays

Cell growth was examined using Celigo (Nexcelom, Lawrence, MA) with EGFP. Kidney tumour cells in each experimental group in logarithmic growth phase were made into cell suspensions using trypsin. Cells were then seeded in 96‐well plates at a density of 1 × 10^3^ or 2 × 10^3^ cells/well. Each group had three plates, and the culture system was 100 μl per plate. The cells were cultured at 37℃. From the second day after plating, the plates were scanned once a day by Celigo (Nexcelom, Lawrence, MA) for 5 days.

### 2.7 MTT assay

Cell growth status was determined using a routine MTT assay. Briefly, kidney tumour cells infected with lentiviruses either shCtrl or shKPNA2 were made into cell suspensions. The cells in the logarithmic phase were then seeded in 96‐well plates at a density of 2 × 10^3^ cells/well. The cell growth data were determined each day for 5 continuous days. 20 μL of 5 mg/mL MTT was added to the wells 4 hours before removing the media at each day. And 100 μL dimethyl sulphoxide (DMSO) was then added to dissolve the resultant MTT formazan. The optical density value was measured at 490/570 nm using a microplate spectrophotometer (Tecan Infinite, Hombrechtikon, Switzerland). The experiments were repeated three times, and the data were statistically analysed and the cell growth curves were drawn.

### 2.8 Cell cycle analysis

After infected with target virus, cell cycle distribution was investigated to evaluate the effect of KPNA2 on kidney tumour cell. Briefly, the cells were made into cell suspensions using trypsin and collected in a 5‐mL centrifuge tube. The cells were centrifuged at 1300 rpm for 5 minutes, and then, the supernatant was discarded. Then, the cells were washed by D‐Hanks (pH 7.2–7.4). The cells were centrifuged at 1300 rpm for 5 minutes once again and then fixed using 75% ethanol for at least 1 hour. The cells were centrifuged at 1300 rpm for 5 minutes and then washed by D‐Hanks (pH 7.2–7.4) once again. 40 × propidium iodide and 100 × RNase were fixed and added to the cells (0.6–1 mL). Cell cycles of these cells were then examined by flow cytometer (Millipore, Darmstadt, Germany). In addition, the analysis was performed with ModFit LT 5.0 software (Becton Dickinson).

### 2.9 Cell apoptosis

The apoptosis of transfected cells was examined by the annexin V‐APC assay followed by flow cytometry. Briefly, the cells infected by lentiviruses were cultured for 3 days. Then, the cells were collected and washed with PBS. The cells were suspended using 200 μL 1 × binding buffer at a final density of 1 × 10^6^/ml. Then, 10 μL annexin V‐APC (eBioscience, Waltham, MA) was added to 100‐μL cell suspensions in a dark place at room temperature for 10 to 15 minutes. Then, the percentage of the apoptosis of the cells was examined by flow cytometer (Millipore, Germany).

### 2.10 Wound healing assay

Kidney tumour cells in in the logarithmic phase were seeded on a collagen type 1‐coated, 6‐well plate and grown overnight to 80% confluence. After overnight serum starvation, a wound was created using a P200 pipette tip. The culture wound was photographed at time 0, 4 and 8 h later, and the rate of closure was assessed.

### 2.11 Transwell assay

Migration was performed using the transwell filter chambers (Costar, Corning, USA) according to the manufacturer's instructions. Briefly, 2 × 10^3^ cells were suspended in serum‐free medium and added into the top chamber. Medium with 10% FBS was added to the lower chamber. After 12 h of incubation, cells on the lower surface of the membrane were stained, photographed and counted using a microscope in six random fields per field for each group. For invasion assay, the porous filter is overlaid by a thin layer of ECM, before cells were seeded into the top chamber. The ECM occludes the membrane pores, blocking non‐invasive cells from migrating. These experiments were performed in triplicate. For proliferation assay, cells were seeded into 96‐well plates (3000/well), and cell viability was determined using Cell Counting Kit‐8 (APExBIO Dojindo Laboratories, Houston, USA) every 24 h.

### 2.12 Co‐immunoprecipitation mass spectrometry (Co‐IP/MS) analysis

ACHN cells were harvested 48h after transfected with KPNA2‐encoding lentivirus or control lentivirus. 1 × 10^6^ cells were digested and lysed in lysis buffer (50 mM Tris‐HCl, pH 8.0; 150 mM NaCl; 0.5% NP‐40; 1 mM EDTA; and protease inhibitors cocktail) for 30 min at 4℃ after they were washed twice with PBS buffer. Then, lysed tissue supernatant was coupled with FLAG‐beads (Sigma) to acquire the co‐immunoprecipitated products. A negative control was performed to collect the co‐immunoprecipitated products without KPNA2‐3 × FLAG. The enriched co‐immunoprecipitation products were separated in 12% SDS‐polyacrylamide gel electrophoresis (PAGE), which was then analysed by mass spectrometry with the assistance of Shanghai Applied Protein Technology Co., Ltd. (China) using LC/MS. The mass spectrometry–derived data were searched with MASCOT2.6 search engines incorporated in the Proteome Discoverer suite, version 2.1 (Thermo Fischer Scientific, Bremen, Germany). Peptides with scores <20 were removed, and higher scores meant a better degree of matching with the secondary atlas. Peptides were searched and compared qualitatively in UniProt. The UniquePep Count and the Cover Percent were also evaluated as auxiliary metrics for the final identification results. 5800 MALDI‐TOF/TOF (AB Sciex, USA) was used for mass spectrometer (MS) data analyses. For quantitative analysis, a protein must have at minimum one unique peptide match with the MS ratios. A ≥ 3.0‐fold or ≤3.0‐fold cut‐off value was used to identify up‐regulated and down‐regulated proteins with a p value <0.05.

### 2.13 In *vivo* experiments

The Institutional Animal Care and Use Committee of Fujian Medical University has approved our animal studies. All animal experiments were carried out in accordance with the guidelines of Institutional Animal Care and Use Committee of Fujian Medical University. Four‐week‐old female BALB/c nude mice, purchased from GemPharmatech, were used. Five female nude mice were inoculated per group. A total of 2 × 10^7^ ACHN cells were coinjected into the right armpit of mice. By day 9 after incubation of the tumour cells, the body weight and tumour volume were measured every 7 days. Animals were sacrificed 49 days postinjection. Luciferase was measured by an imaging system to evaluate the tumour area.

### 2.14 Immunofluorescence (IF) staining

ACHN cells were washed gently with PBS and fixed with 4% paraformaldehyde (PFA) for 15 min. Next, the cells were permeabilized using 0.025% Triton X‐100 (Beyotime, Shanghai, China) for 10 min. Cells were then incubated with 1% normal goat serum (Solarbio) for 1 h and incubated with primary antibodies overnight at 4℃. Then, cells were washed and incubated with an Alexa Fluor® 594 secondary antibody for 1 h at room temperature. Finally, nuclear staining was performed with DAPI (Beyotime) for 10 min. Representative images were acquired using a confocal microscope (Olympus, Japan).

### 2.15 Statistical analysis

The expression of KPNA2 and related clinical data for ccRCC patients is available in TCGA database. A total of 534 primary KICR samples from patients, including 326 from clinical stage I/II, 205 from clinical stage III/IV and 3 unreported cases, were obtained. The expression and clinical information were analysis. Expression level of KPNA2 from the early stage and late stage was evaluated. Two groups were divided according to the median expression level of each gene. Survival differences among groups were estimated using the R package ‘survival’. The Kaplan‐Meier curve was constructed, and a log‐rank test was used to determine differences among overall survival and progression‐free survival according to the KPNA2 mRNA levels in this study.

## 3 RESULTS

### 3.1 KPNA2 is up‐regulated in ccRCC

**FIGURE 1:**
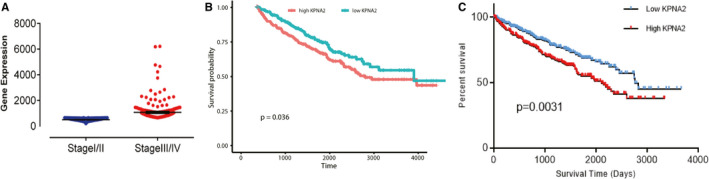
KPNA2 is correlated with the development of ccRCC. A, Gene expression analysis showed that KPNA2 was significantly up‐regulated in clinical stage III/IV patients compared with stage I/II patients. B, Kaplan‐Meier analysis showed that the expression of KPNA2 was significantly associated with poor overall survival rate in ccRCC patients (*P*‐value <0.05, log‐rank test). C, The high expression of KPNA2 showed a poor progression‐free survival (PFS) rate in ccRCC patients (P‐value <0.05, log‐rank test)

To investigate the role of KPNA2 related to the development of ccRCC, samples from four stages were collected. Based on the RNA‐seq data set from 525 tumour samples, we found the expression level of KPNA2 was significantly higher in clinical stage III/IV patients compared with clinical stage I/II patients (Figure 1A). Patients with low expression levels of KPNA2 had a better overall survival rate than those with high expression levels of KPNA2(Figure 1B). Similarly, the high expression of KPNA2 showed a poor progression‐free survival (PFS) rate in ccRCC patients (Figure 1C). Thus, higher expression of KPNA2 might play an important role in the development and progression of ccRCC, which makes it one of the promising biomarkers or therapy target.

### 3.2 Knockdown of KPNA2 inhibits kidney tumour cell growth

**FIGURE 2:**
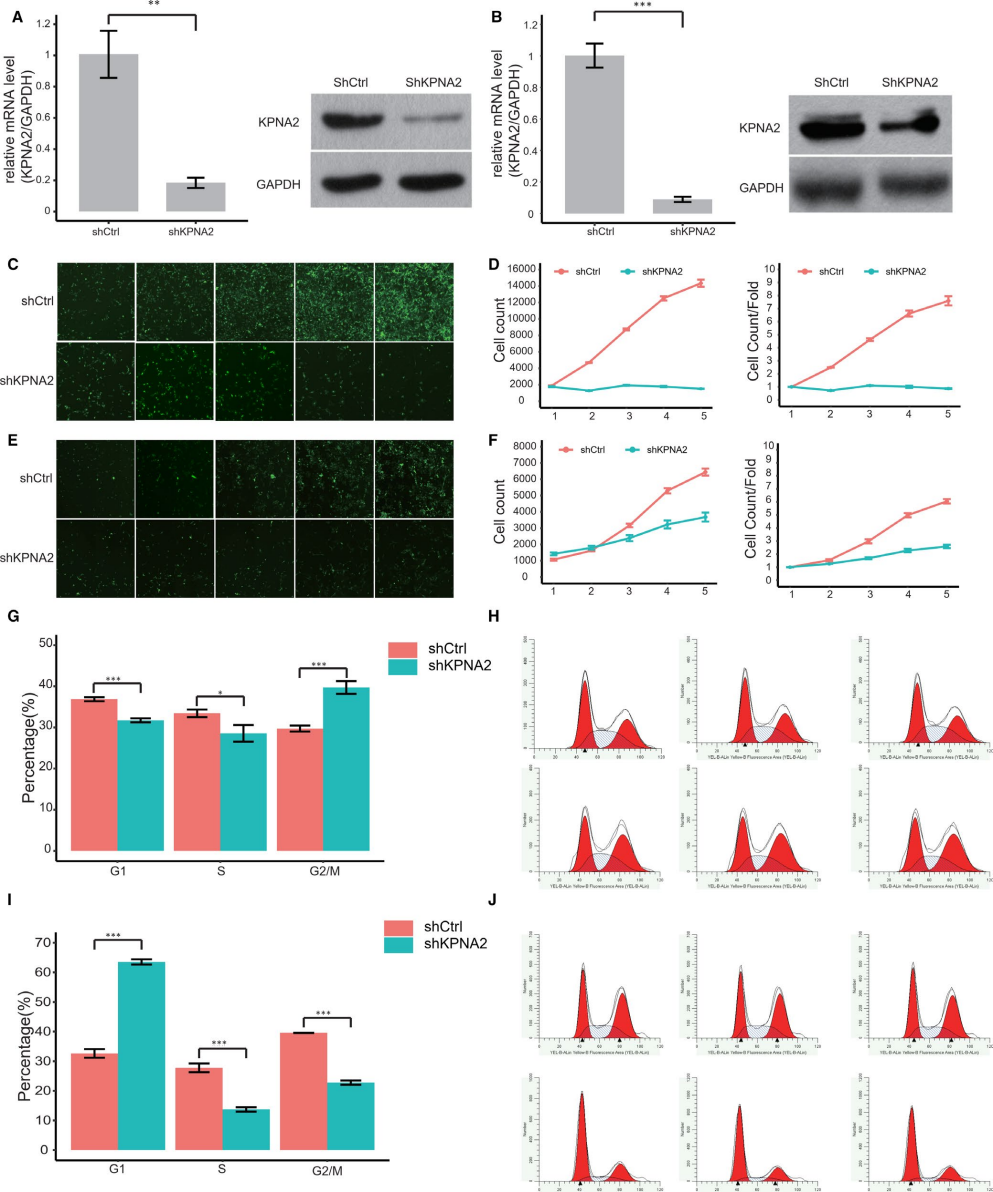
KPNA2 is essential for kidney tumour cell growth. RT‐PCR and Western blotting analysis of KPNA2 knockdown in (A) 786‐O cells and (B) ACHN cells with lentiviruses expressing either shCtrl or shKPNA2. GAPDH was used as the loading control. After knockdown of KPNA2, the cells with GFP fluorescence detected by Celigo significantly decreased in 5 days following after 3 days postinfection in (C) 786‐O cells and (E) ACHN cells. Total count of cell and proliferation rate was plotted for (D) 786‐O cells and (F) ACHN cells. Cells arrested in G1, S and G2/M phase after silencing KNPA2 in (G and H) 786‐O cells and (I and J) ACHN cells (*p<0.05, **p<0.01 and ***p<0.001)

The expression of KPNA2 was examined in three types of kidney tumour cell lines, including 786‐O, ACHN and Caki‐1. Using GAPDH as a control, KPNA2 in all tumour cell lines was strongly expressed (Figure S2). In order to investigate the role of KPNA2 in kidney tumour, functional studies were performed in 786‐O and ACHN cell lines. The expression of KPNA2 was down‐regulated with the lentiviral vector construction shKPNA2. The effective transfection of shKPNA2 was examined at 3 days postinjection using real‐time PCR and Western blot (Figure 2A and 2B). The expression of KPNA2 in 786‐O reduced 80% compared with the control. More effective depletion was found in ACHN cells through transfection of shKPNA2 (91%). Therefore, the possible function of KPNA2 in kidney tumour cell proliferation was investigated at the following five days after 3 days postinjection. Celigo assay proved a predominant effect of shKPNA2 to the proliferation of kidney tumour cells after effective knockdown of KPNA2. The cells with GFP fluorescent detected by Celigo significantly decreased in both cell lines, suggesting knockdown of KPNA2 could inhibit tumour development (Figure 2C‐E). The total counts of tumour cell slightly changed in 786‐O cell, while tight increment was found in ACHN cell. Cell growth rate in both cell lines with KPNA2 knockdown was significantly lower than control (Figure 2D/F). Previous studies have demonstrated the knockdown of KPNA2 led to significant cell cycle arrest in HepG2 cells ^38^. We further examined the cell cycle distribution of kidney tumour cells after deregulated KPNA2. In 786‐O cells, the flow cytometry showed that knockdown of KPNA2 significantly increased the amount of G2/M phase cells and reduced the amount of G1 and S phase cells (*p*<0.01). Silencing of KPNA2 significantly increased the percentage of cells in G1 phase in ACHN cells (*p*<0.01, Figure 2E and 2F). The results showed that 786‐O cells and ACHN cells were arrested at different stages.

### 3.3 Knockdown of KPNA2 promotes kidney tumour cell apoptosis

**FIGURE 3:**
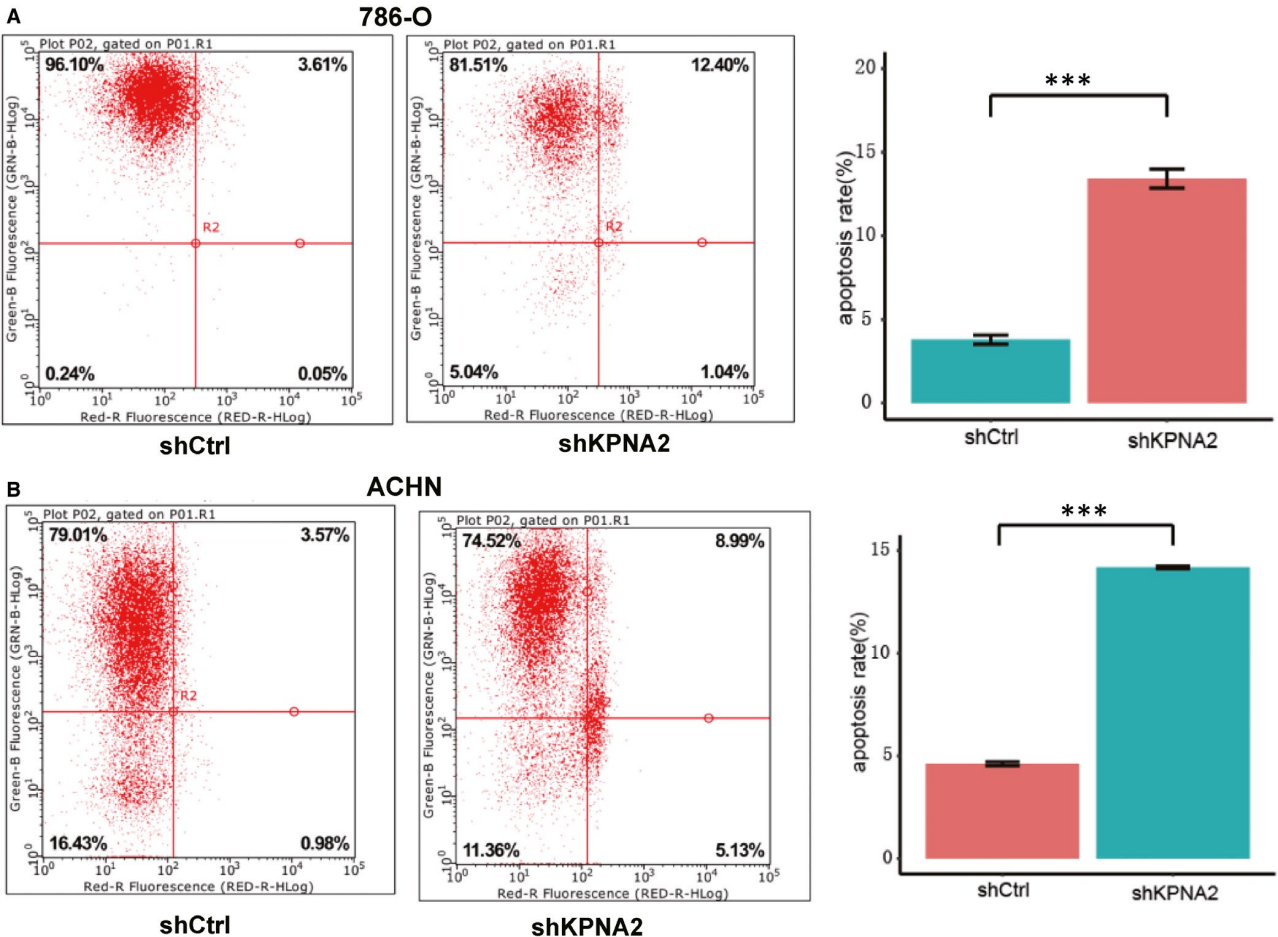
Knockdown of KPNA2 promotes the apoptosis rate. The percentage of apoptotic cells in (A) 786‐O cells and (B) ACHN cells infected with lentiviruses expressing either shCtrl or shKPNA2 (***p<0.001)

Subsequently, we investigated whether the apoptotic cell death could be affected by the KPNA2. Then, flow cytometry was performed. As shown in Figure 3A and 3B A significant increase in apoptosis phenomenon was observed in shKPNA2 cells. Both in 786‐O cells and in ACHN cells, apoptosis was found in less than 5% cells infected with lentiviruses without shKPNA2, while an apoptosis rate over 10% was observed in KPNA2 knockdown cells. The results indicated that knockdown of KPNA2 promotes kidney tumour cell apoptosis.

### 3.4 Knockdown of KPNA2 inhibits kidney tumour cell migration and invasion

**FIGURE 4:**
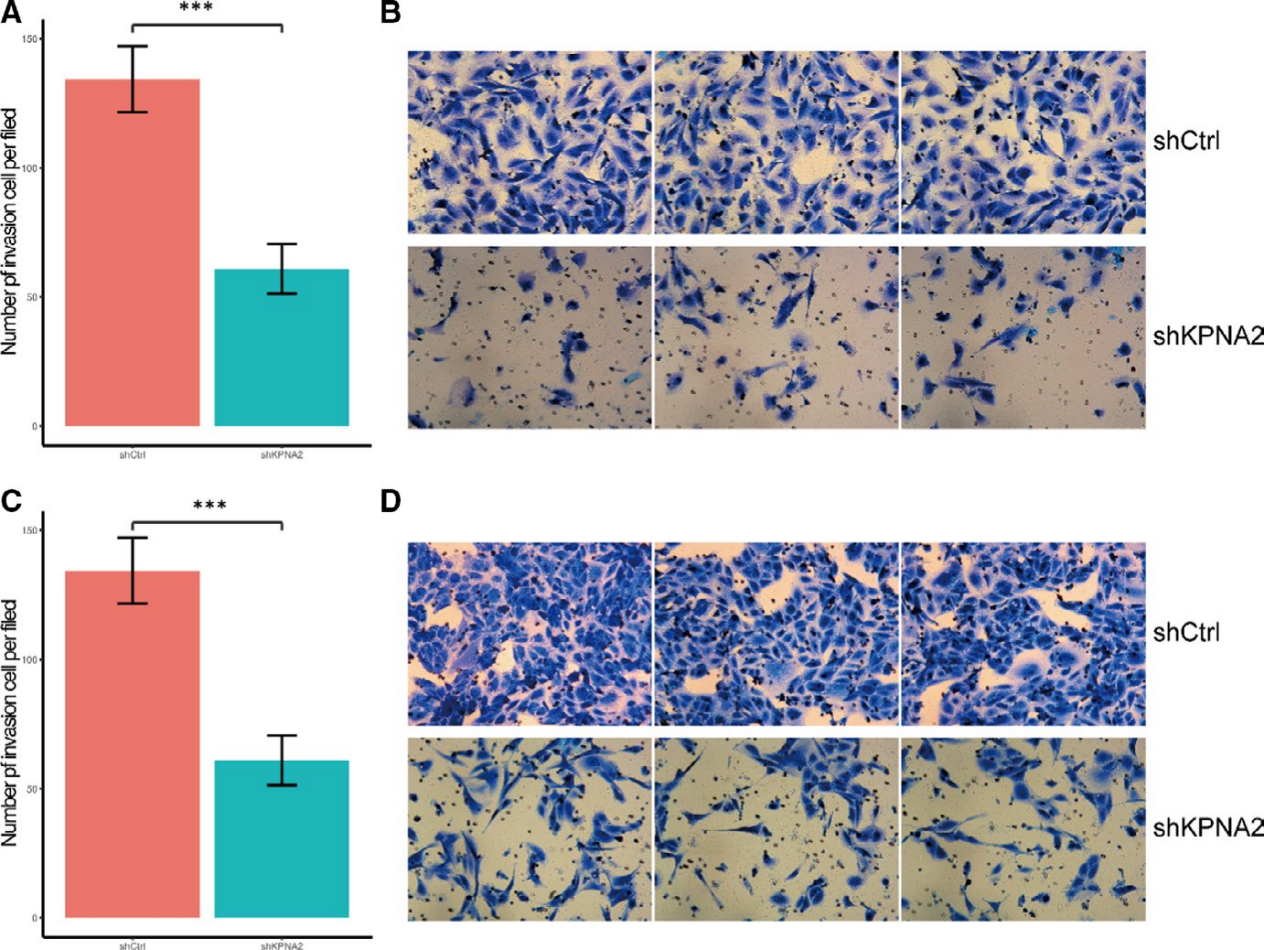
Silencing KPNA2 reduced the invasion process of 786‐O/ACHN cells. Transwell migration assay of A/B) 786‐O and C/D) ACHN cells infected with lentiviruses expressing either shCtrl or shKPNA2. Quantification of invasive properties induced by foetal bovine serum in KPNA2 knockdown and control groups in (A) 786‐O and (C) ACHN cells. Invasion ability analysed by the transwell migration assay in (B) 786‐O and (D) ACHN cells with different KPNA2‐expression pattern (×200; *p < 0.001). Data were obtained from three independent experiments and yielded similar results

Recent studies also indicated that high expression of KPNA2 was correlated with the development and progression of several tumours^21^, ^39^. We further assessed the effect of KPNA2 on cell migration and invasion. When KPNA2 was down‐regulated, the migration of cells increased slightly compared with the control cells in the following 8 hours (Figure S3 and S4). However, the invasion ability was significantly reduced when KPNA2 was down‐regulated (*p*<0.05, Figure 4). These *in vitro* data demonstrated that KPNA2 has an impact on metastasis of kidney cancer cells.

### 3.5 Repression of KPNA2 reduced the development of kidney cancer in *vivo*

**FIGURE 5:**
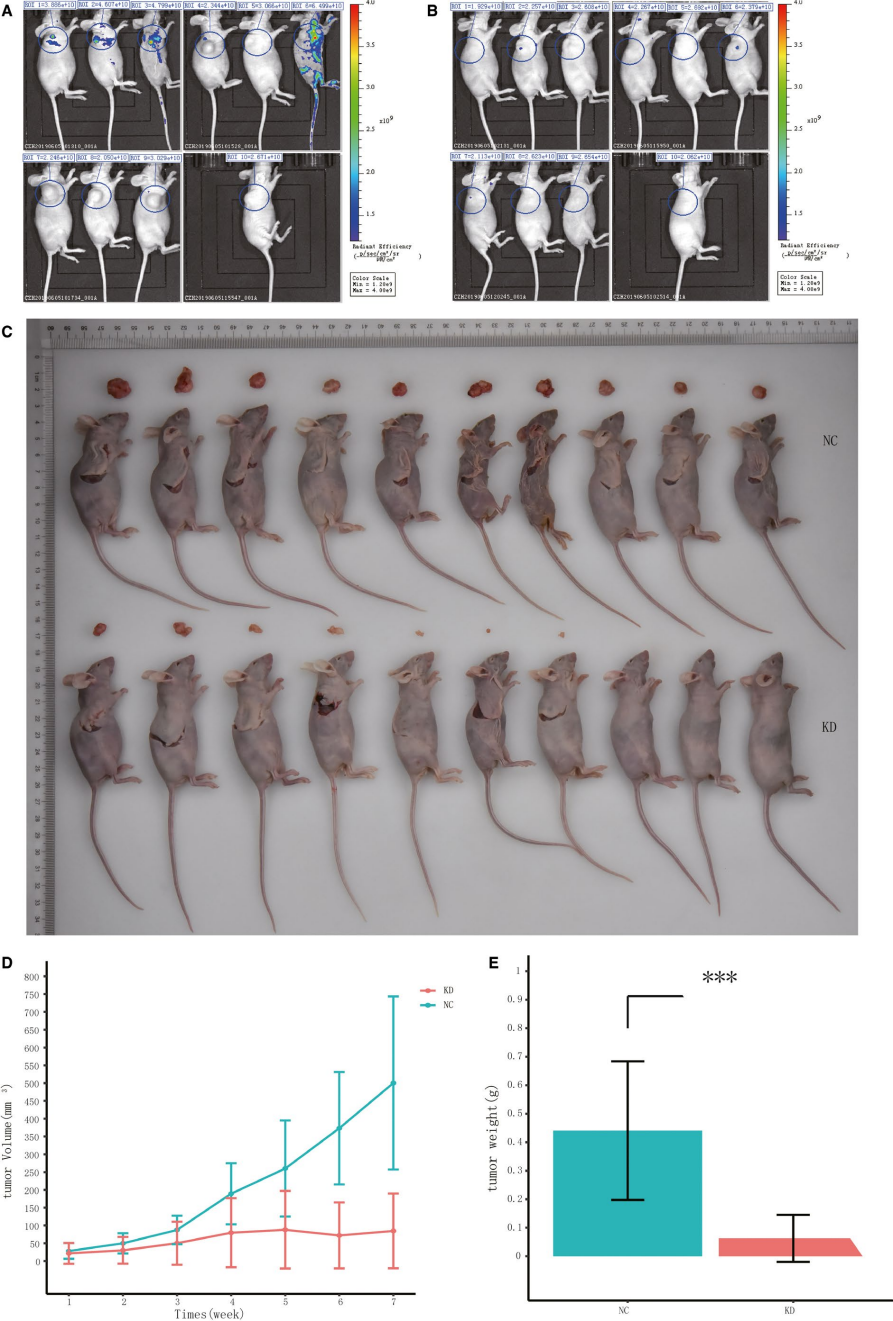
Silencing KPNA2 reduced the development of ccRCC in *vivo*. Bioluminescence imaging was performed in mice after infected with ACHN cells expressing shKPNA2 (KD) or shCtrl (NC) in the following seven weeks. (A) Bioluminescence imaging was performed in control mice (NC). (B) Bioluminescence imaging was performed in KPNA2 knockdown mice (KD). (C) All of the mice were killed, and the kidney tumours were collected. Visible kidney metastases and body of control mice(top) and KPNA2‐silencing mice (bottom) are shown. Tumour volumes (D) and tumour weight (E) of subcutaneous injection models of KPNA‐silencing and control mice are shown

To confirm the oncogenic activity of KPNA2 in *vivo*, we established a BALB/c nude mouse model using ACHN cells with shKPNA2 or shCtrl. After 4 weeks, ACHN cells with shKPNA2 or shCtrl were microinjected into right armpit of female nude mice and the tumour volume was measured every 7 days until day 49. The luciferase was employed to measure the tumour area, as well as tumour metastasis and severity (Figure 5A and 5B). Bioluminescence imaging in living mice clearly showed that EGFP signals were significantly higher in the NC group than those in the shKPNA2 group. Statistical analysis demonstrated that the tumour volume and weight of mice treated with the shKPNA2 were significantly decreased relative to those of NC (*p*<0.05, Figure 5C‐E). Mice in the shKPNA2 group exhibited an obviously repressed development of metastasis than those in the NC group. These data proved that knockdown of KPNA2 is sufficient to repress the development of tumour.

### 3.6 NPM1 was one of the targets of KPNA2

**FIGURE 6:**
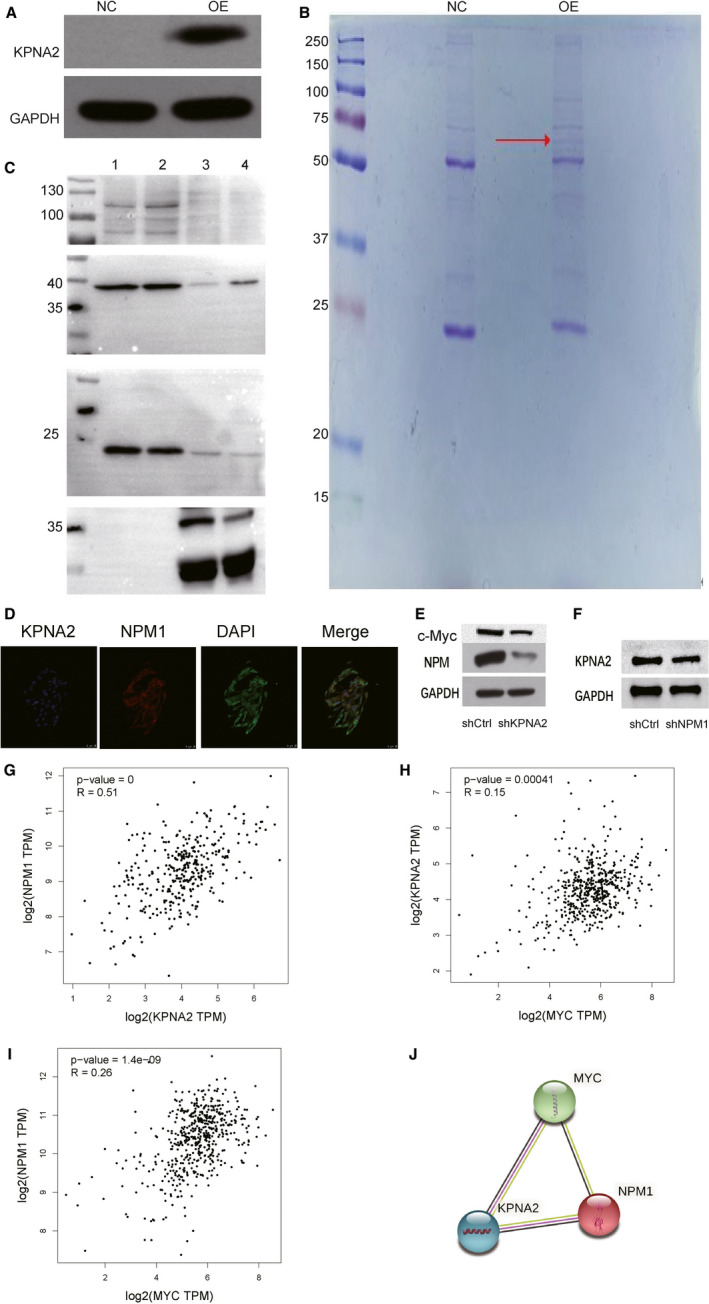
Purification of KPNA2‐interacting proteins using co‐immunoprecipitation. A, SDS‐PAGE separation of the immunoprecipitates obtained from ACHN cells infected with KPNA2 overexpression (OE) or control (NC). GAPDH was used as an internal control, and the gel was visualized using silver staining. B, The total cell lysate extracted from ACHN cells in the NC or OE group was assessed by affinity purification. The immunoprecipitates were separated on SDS‐PAGE and stained by Coomassie Blue. The target lane was excised and analysed by mass spectrometry. The red arrow indicated the KPNA2 protein. C, Western blot of four candidate proteins. From top to bottom: MCM3 (110 kDa), NPM1 (38 kDa), RAN (24 kDa) and YBX1 (36 kDa). The lanes 1,2,3 and 4 are NC‐input, OE‐input, NC‐IP and OE‐IP. D, Double IF staining for KPNA2 and NPM1 in ACHN cells to observe their localization status. E, The NPM1 and c‐Myc protein expression level in ACHN cells after KPNA2 knockdown. F, The KPNA2 protein expression level in ACHN cells after NPM1 knockdown. G‐I, The Pearson correlation analysis between KPNA2 and NPM1, MYC and KPNA2, MYC and NPM1 by the GEPIA database.*(*p*<0.05) (http://gepia.cancer‐pku.cn/). J, The protein protein interaction (PPI) network of KPNA2 NPM1 and MYC

To screen out the interacting proteins of KPNA2, we conducted co‐immunoprecipitation mass spectrometry (Co‐IP/MS) analysis with KPNA2‐overexpressed ACHN cell line. The full length of KPNA2, tagged with 3 × FLAG at the N‐terminal, was cloned into the lentiviral vector, which was then transfected into ACHN cell line. Lentiviral vector without KPNA2 was used as control. Total proteins were extracted using lysis buffer. The anti‐3 × FLAG antibody was conjugated to the surface of magnetic beads using DSS. Then, protein extract was incubated with the bead isolate KPNA2 and its interacting proteins. The obtained immunoprecipitates in KPNA2 overexpression and control were separated using SDS‐PAGE (Figure 6A‐B), and the target gel lane was digested with trypsin for LC‐MS/MS analyses. A total of 853 peptides were found in KPNA2 overexpression cells, compared with 341 peptides in control cells (Table S1; TableS2). 209 proteins were identified with relatively high expression level in KPNA2 overexpression cells (Table S3). Western blotting analysis was performed to evaluate the expression level of four candidate KPNA2 interaction proteins from Table S3 (Figure 6C). Co‐IP results showed a specific interaction between KPNA2 and NPM1(Figure 6C). IF staining further validated a considerable degree of colocalization for KPNA2 and NPM1 in ACHN cells. The expression of two proteins was colocalized in the nucleus (Figure 6D). Then, Western blotting of NPM1 with KPNA2 knockdown in ACHN cells was performed. We found that NPM1 expression was consistent with KPNA2 (Figure 6E‐F). Also, c‐Myc was down‐regulated after KPNA2 knockdown (Figure 6E). A previous study indicates that KPNA2 can transport c‐Myc into the nucleus, which could trigger tumorigenicity of cells ^39^, and c‐Myc can directly bind to the NPM1 promoter to induce its expression ^40^. Further, the expression of KPNA2 is positively correlated with NPM1 in kidney cancer (Figure 6G). Also, we found that correlation between Myc and KPNA2, Myc and NPM1 was significantly positive in KIRC (Figure 6H‐I). Furthermore, by using the String database(https://www.string‐db.org/), we found that KPNA2, NPM1 and Myc interact closely with each other (Figure 6J). So, KPNA2 may regulate NPM1 via c‐Myc to promote the proliferation of kidney cancer cells.

### 3.7 KPNA2 promotes kidney cancer cell proliferation and migration by targeting NPM1

**FIGURE 7:**
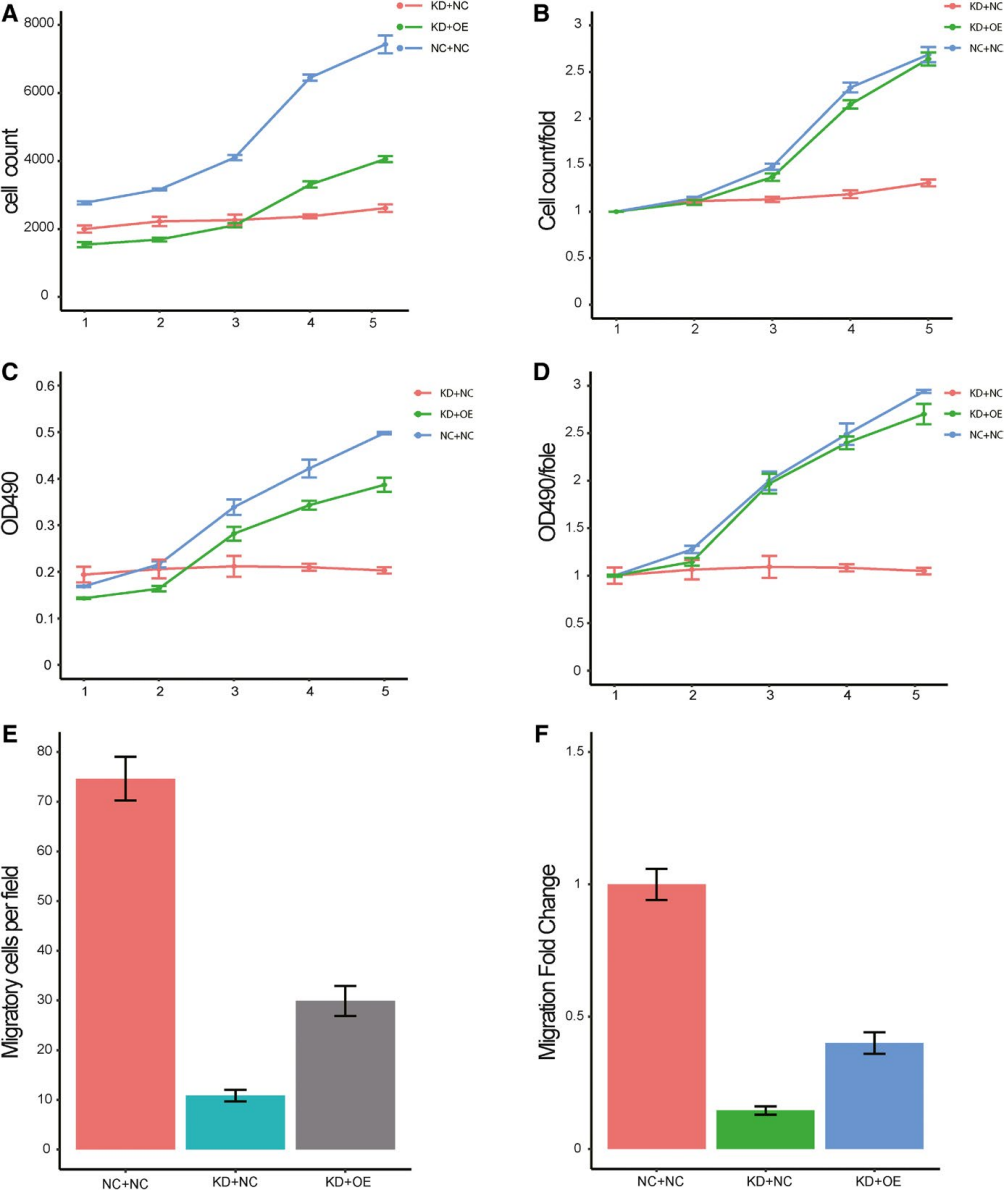
NPM1 promotes metastasis in ACHN cell line. ACHN cells were infected by shKPNA2 (KD+NC), shKPNA2 +NPM1 lentivirus (KD+OE) and control group (NC+NC). A, B, Cell counts and fold change in three groups in the following five days after infection process. C, D, OD 490 value and change fold in the three groups of cells. E/F) Transwell assay evaluated the invasion of cells in the three groups of cells

To assess whether NPM1 contributes to the proliferation and migration progression of kidney cancer through the regulation of KPNA2, we transfected KPNA2‐targeting siRNA into ACHN cells with forced expression of NPM1. As shown in Figure 7, overexpression of NPM1 attenuated the repression of silencing KPNA2 on cell proliferation. The proliferation rate significantly reduced after silencing KPNA2, while increasing the expression of NPM1 led to the similar proliferation rate compared with control cells, which was also indicted by MTT assay results. Furthermore, overexpression of NPM1 also promoted the migration and invasion of cancer cells even though KPNA2 was repressed. These data suggested that NPM1 could rescue phenotypic deletion caused by KPNA2 knockdown in ACHN cells.

## 4 DISCUSSION

As one of the major roles involved in nucleocytoplasmic transport process, KPNA2 has been proved to be related to proliferation, migration and invasion of tumour cells ^17^, ^22^, ^23^, ^41^, ^42^, ^43^, ^44^. According to a previously published report ^45^, KPNA2 is essential for some cancer, whereas it is not essential for some cancer. In the analysis of hepatocellular carcinoma tumour, KPNA2 was up‐regulated in HCC tumours compared with its normal liver tissue, while changing the expression of KPNA2 successfully modulated the proliferation of HCC cells ^45^. In our study, we firstly evaluated the different expression levels of KPNA2 among patients with various development stages of ccRCC. The results proved KPNA2 was up‐regulated in the late stage of ccRCC, and higher KPNA2 is associated with poor survival rate of patients, demonstrating that the expression level of KPNA2 in tumour tissue has a good prognostic value for ccRCC. We then investigated the effect of KPNA2 knocked down on kidney cancer cell lines. Using siRNA, the expression level of KPNA2 was suppressed in both of the two cell lines, resulting in the reduced protein after 5 days infection. CCK‐8 assay was employed to detect the cell viability of ACHN/786‐O cells. The results showed that the cell viability was remarkably decreased by silencing KPNA2 in both cell lines. Disruption of KPNA2 could result in decreased tumour cell growth and increased apoptosis though p53 and p21 ^46^. According to the data extracted from our study, compared with ACHN cell line, less KPNA2 was expressed and accumulated, with less viable cells, in 786‐O cell line. Knockdown of *KPNA2* successfully repressed the cell viability of kidney cancer cells. After investigating the proliferation rate and apoptosis rate, we found silencing of KPNA2 increased apoptosis in both cell lines, while the proliferation rate was remarkably decreased.

The cell cycle pathway was proved to be affected by KPNA2, and down‐regulation of KPNA2 attenuated c‐Myc transcriptional transactivating activity, which contributes to the transition of G1/S cell cycle ^39^, ^45^. In our data, silencing KPNA2 in ACHN cells significantly increased the percentage of cells at G1 phase, while cells at S or G2/M phase significantly decreased. However, cells in G2/M phase increased after KPNA2 knockdown in 786‐O cell. Our results suggested the dysfunction of KPNA2 in modulating cell cycle process. Cell cycle was abrupted after the repression of KPNA2 with siRNA, which might eventually be helpful to inhibit tumour cell growth in ccRCC. Tumour invasion is also one of the key steps leading to metastasis and poor prognosis ^47^, which could arise from several consecutive mutation events and epigenetic elements. We discovered that invasion and migration in both cancer cells were inhibited after silencing KPNA2.

Based on the Co‐IP/MS analysis, we identified a key protein, NPM1, that could interact with KPNA2. Even though down‐regulation of KPNA2 could significantly repress cancer cell proliferation and further invasion ability, we found that overexpression of NPM1 could promote cell proliferation, as well as the migration ability and invasion of cancer cells in some degree. These results firstly proved the cooperation of KPNA2 and NPM1 in tumorigenesis.

As a well‐known nucleolar phosphoprotein, NPM1 has been found to be strongly correlated with cell proliferation and cancer pathogenesis. This gene has oncogenic and tumour‐suppressing functions through the frequent overexpression or genetic modification ^25^, ^26^, ^27^. Mutation of NPM1 had been proved to be related to leukaemia and lymphoma ^29^, ^30^. Furthermore, NPM1 is overexpressed in many types of major human solid tumours including tumours of colon^31^, ovary ^32^ and prostate and other tumours ^33^, ^34^. Moreover, the oncogenic c‐Myc can directly bind to the NPM1 promoter to induce its expression in leukaemia ^40^. Previous study also proved that overexpression of KPNA2 can transport c‐Myc into the nucleus, which could trigger tumorigenicity of cells ^39^. So, we hypothesize that the overexpression of KPNA2 in ccRCC triggers the up‐regulation of c‐Myc, which further induced the expression of NPM1, and eventually contributes to tumorigenesis. In addition, the detailed mechanism of KPNA2 and NPM1 as an oncogenic factor in cancer cells should be evaluated in future studies.

In this study, we evaluated the expression level of KPNA2 in ccRCC patients from different stages. KPNA2 is overexpressed in the late stage of ccRCC, and its up‐regulation is associated with poor prognosis. Significantly higher expression of KPNA2 was found in the late stage, while higher KPNA2 is significantly associated with poor survival rate. Next, in *in vitro* and *vivo* analysis, our results indicated that KPNA2 may regulate NPM1 to promote the progression of kidney cancer.

## CONFLICT OF INTEREST

The authors confirm that there are no conflicts of interest.

## AUTHOR CONTRIBUTION

**Song Zheng:** Conceptualization (equal); Project administration (equal); Writing‐original draft (equal); Writing‐review & editing (equal). **Xiao‐Fan Li:** Data curation (equal); Formal analysis (equal); Funding acquisition (equal); Software (equal); Visualization (equal); Writing‐review & editing (equal). **Ting Deng:** Data curation (equal); Formal analysis (equal); Software (equal); Visualization (equal). **Rong Liu:** Data curation (equal); Formal analysis (equal); Writing‐original draft (equal). **Junjie Bai:** Investigation (equal); Methodology (equal); Writing‐review & editing (equal). **Teng Zuo:** Data curation (equal); Methodology (equal); Writing‐review & editing (equal). **Yinan Guo:** Data curation (equal); Writing‐review & editing (equal). **Jianhui Chen:** Conceptualization (equal); Data curation (equal); Funding acquisition (equal); Investigation (equal); Project administration (equal); Supervision (equal); Validation (equal); Writing‐original draft (equal); Writing‐review & editing (equal).

## Supporting information

Figure S1

Figure S2

Figure S3

Figure S4

Figure Legends

Table S1

Table S2

Table S3
