# Supplementary figures and images for: KPNA2 promotes renal cell carcinoma proliferation and metastasis via NPM
Source: J Cell Mol Med. 2021 Sep 1;25(19):9255–67. doi: 10.1111/jcmm.16846 (PMC8500977; doi:10.1111/jcmm.16846)

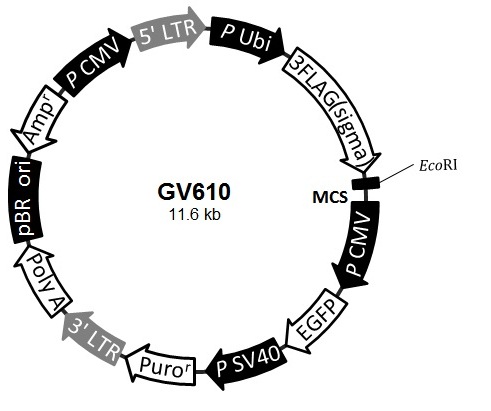

Supplement: Supplementary file 1 — Figure S1 [file JCMM-25-9255-s007.jpg]

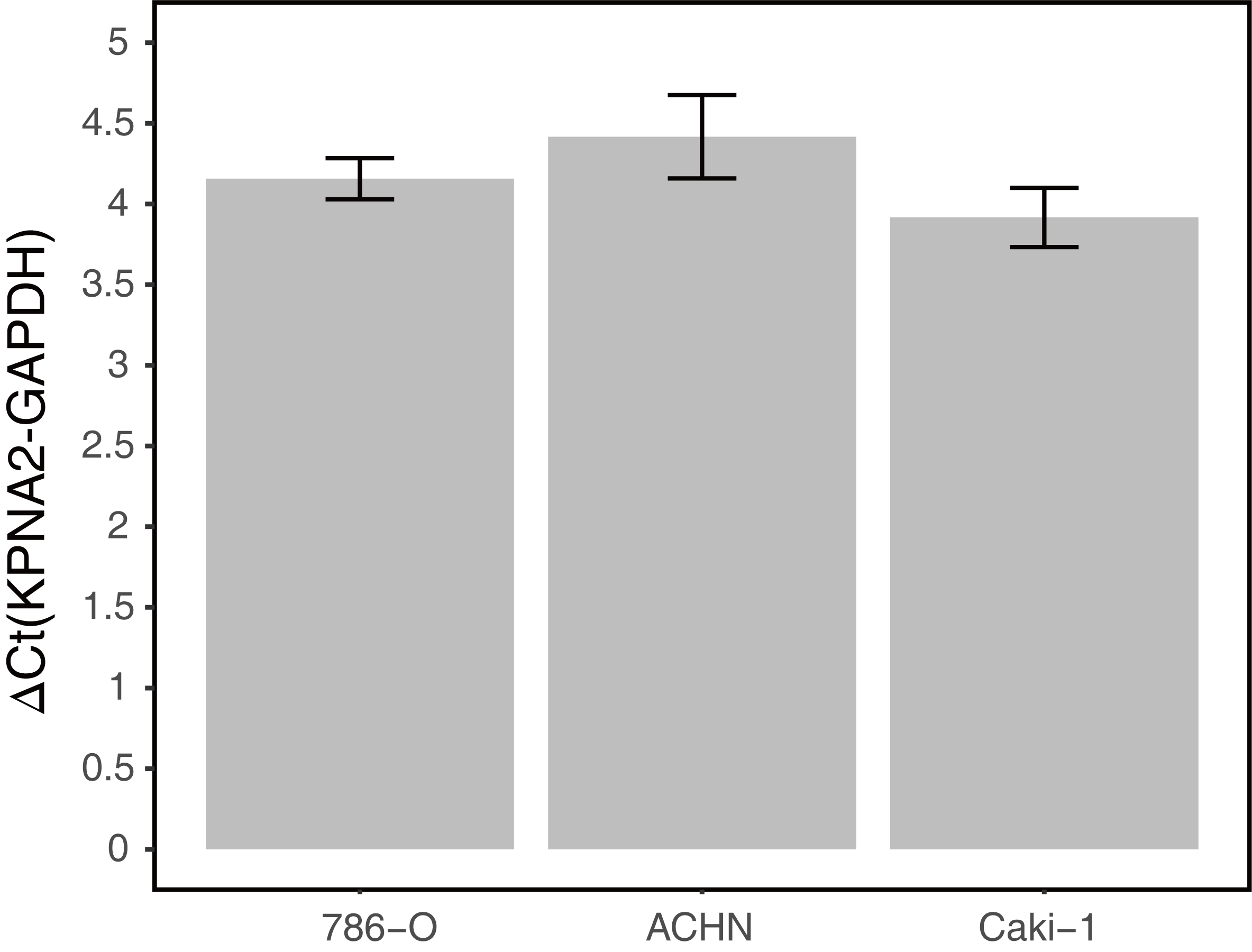

Supplement: Supplementary file 2 — Figure S2 [file JCMM-25-9255-s006.png]

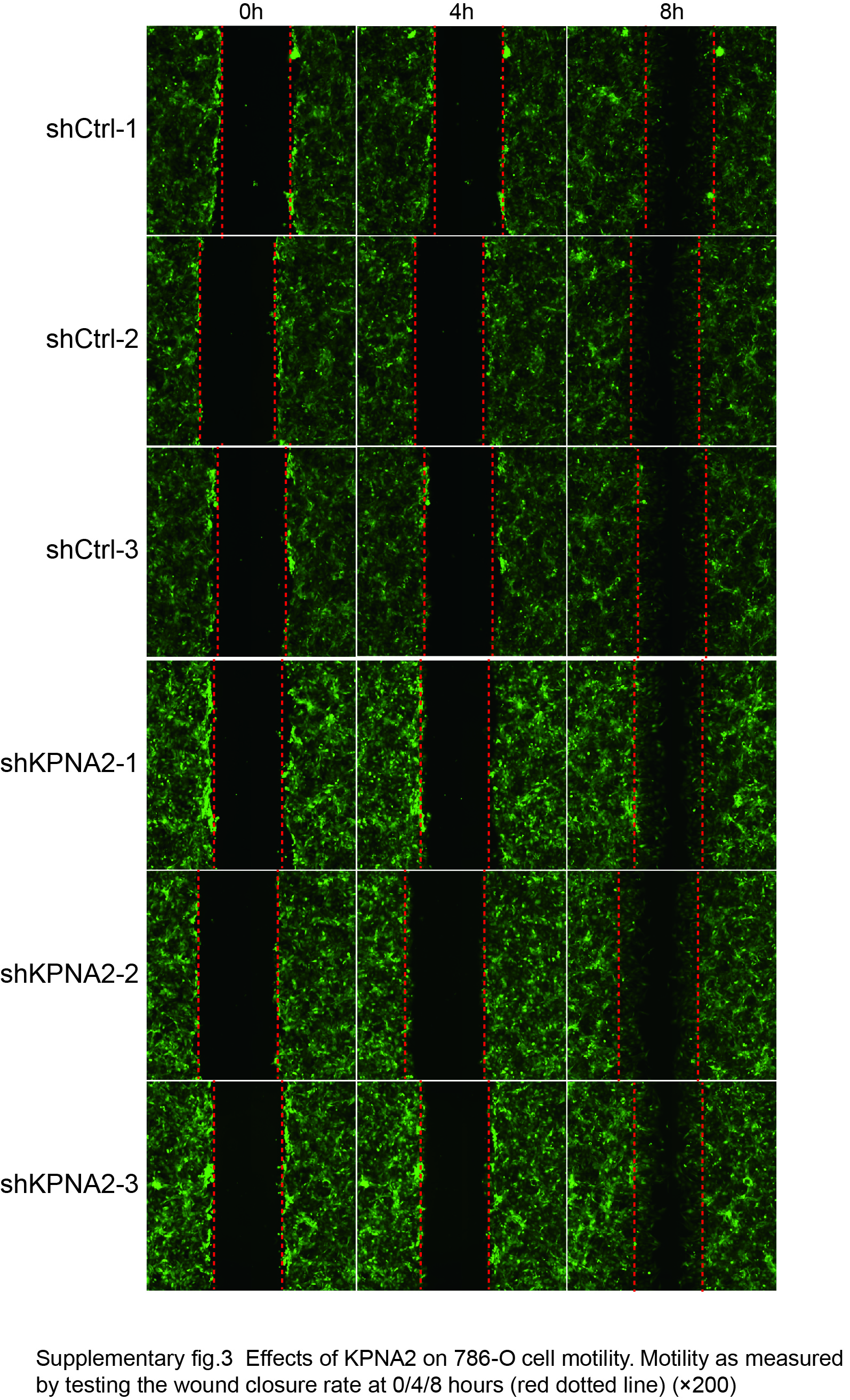

Supplement: Supplementary file 3 — Figure S3 [file JCMM-25-9255-s002.jpg]

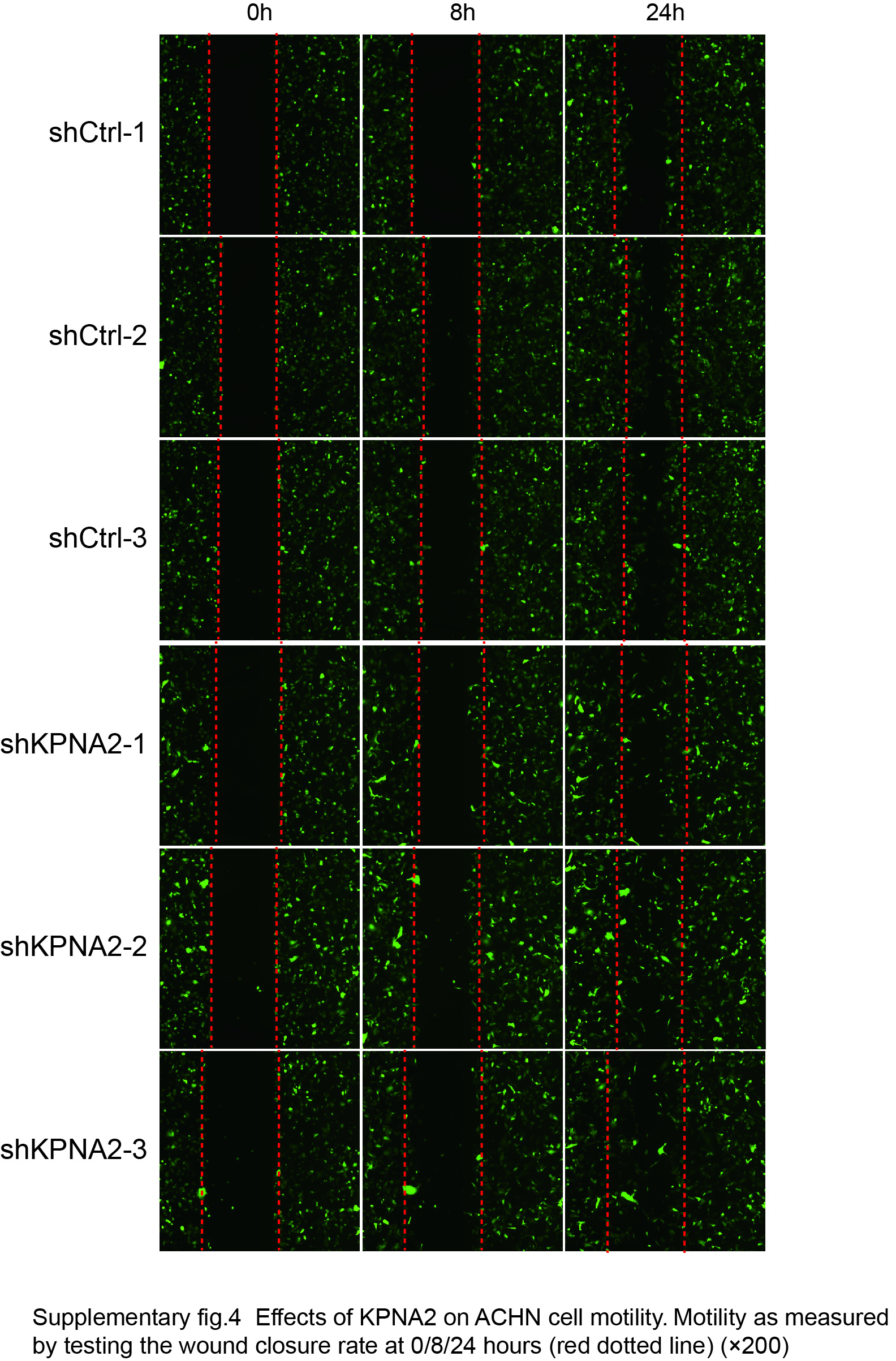

Supplement: Supplementary file 4 — Figure S4 [file JCMM-25-9255-s005.jpg]
