# Supplementary material for: KPNA2 promotes renal cell carcinoma proliferation and metastasis via NPM
Source: J Cell Mol Med. 2021 Sep 1;25(19):9255–67. doi: 10.1111/jcmm.16846 (PMC8500977; doi:10.1111/jcmm.16846)
Supplement: Supplementary file 5 — Figure Legends [file JCMM-25-9255-s001.docx]

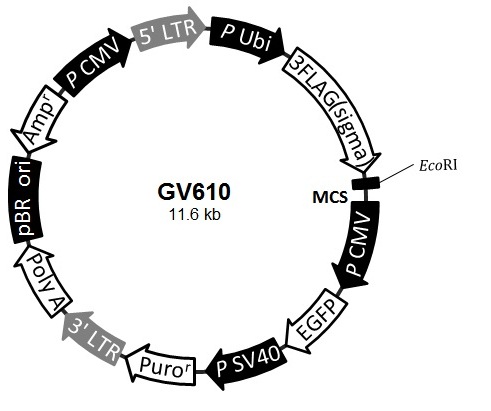


Supplementary fig.1 vectorinformation_of_GV610.


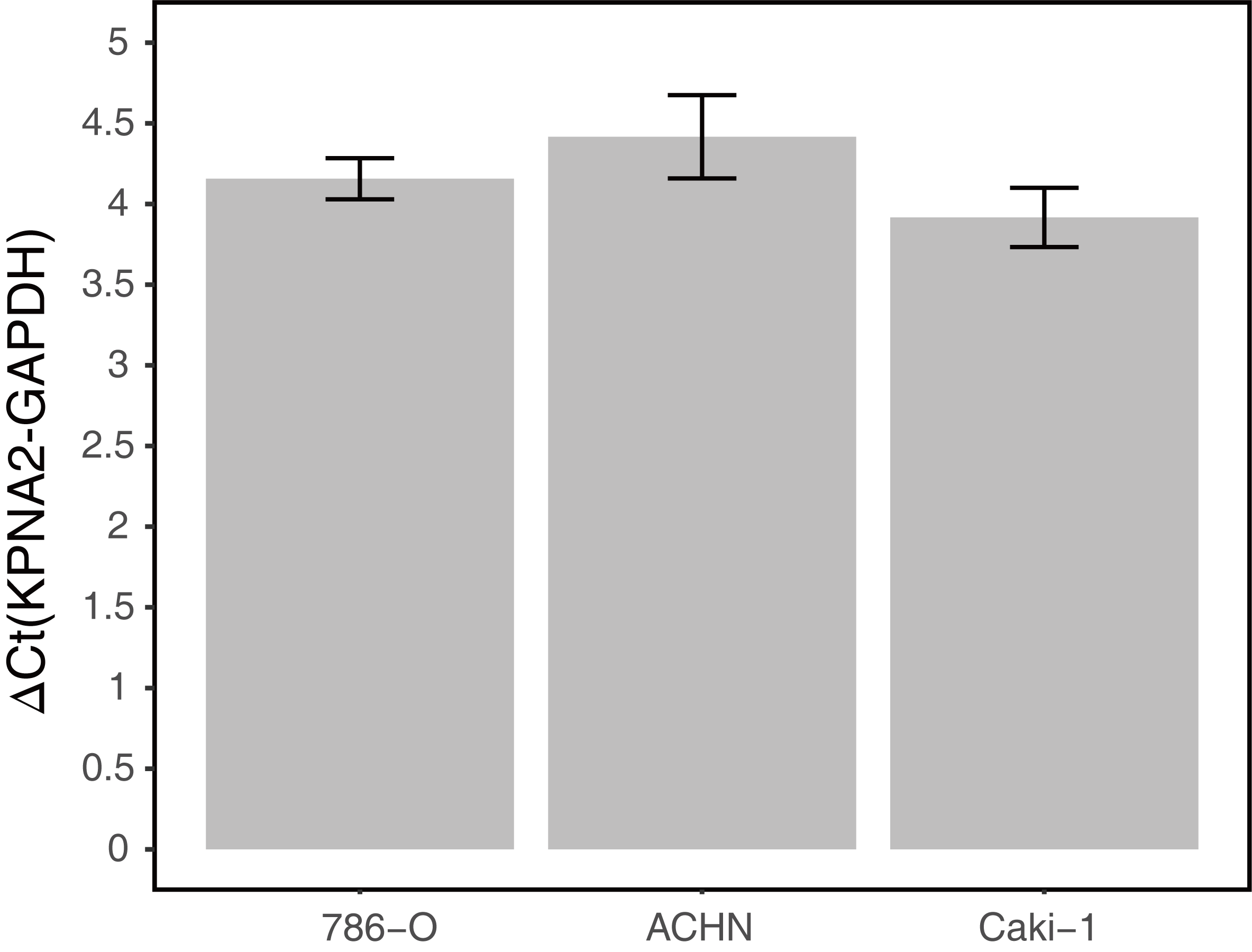


Supplementary fig.2 Expression of KPNA2 in three kidney tumor cells.


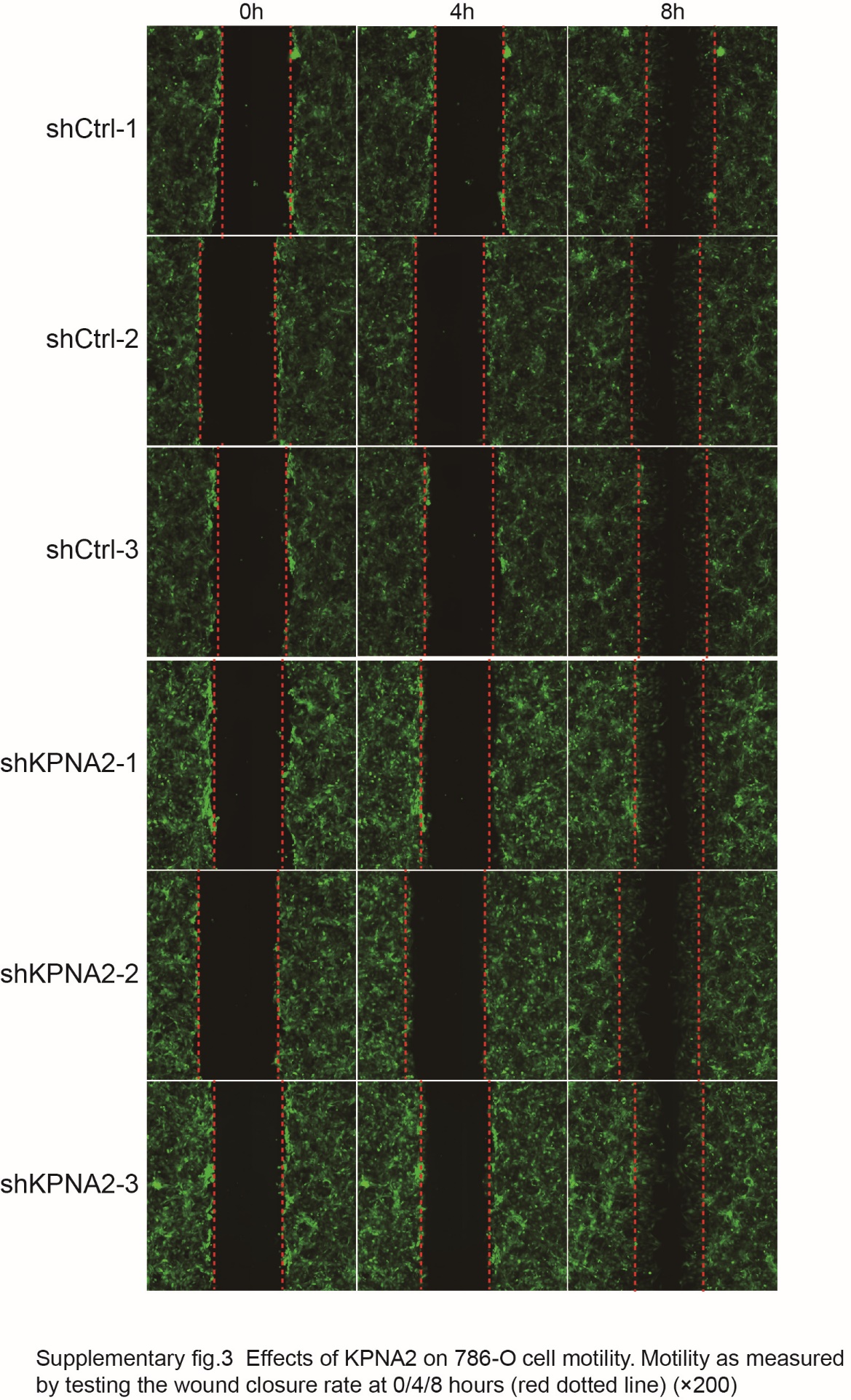
Supplementary fig.3 Effects of KPNA2 on 786-O cell motility. Motility as measured
by testing the wound closure rate at 0/4/8 hours (red dotted line) (×200).


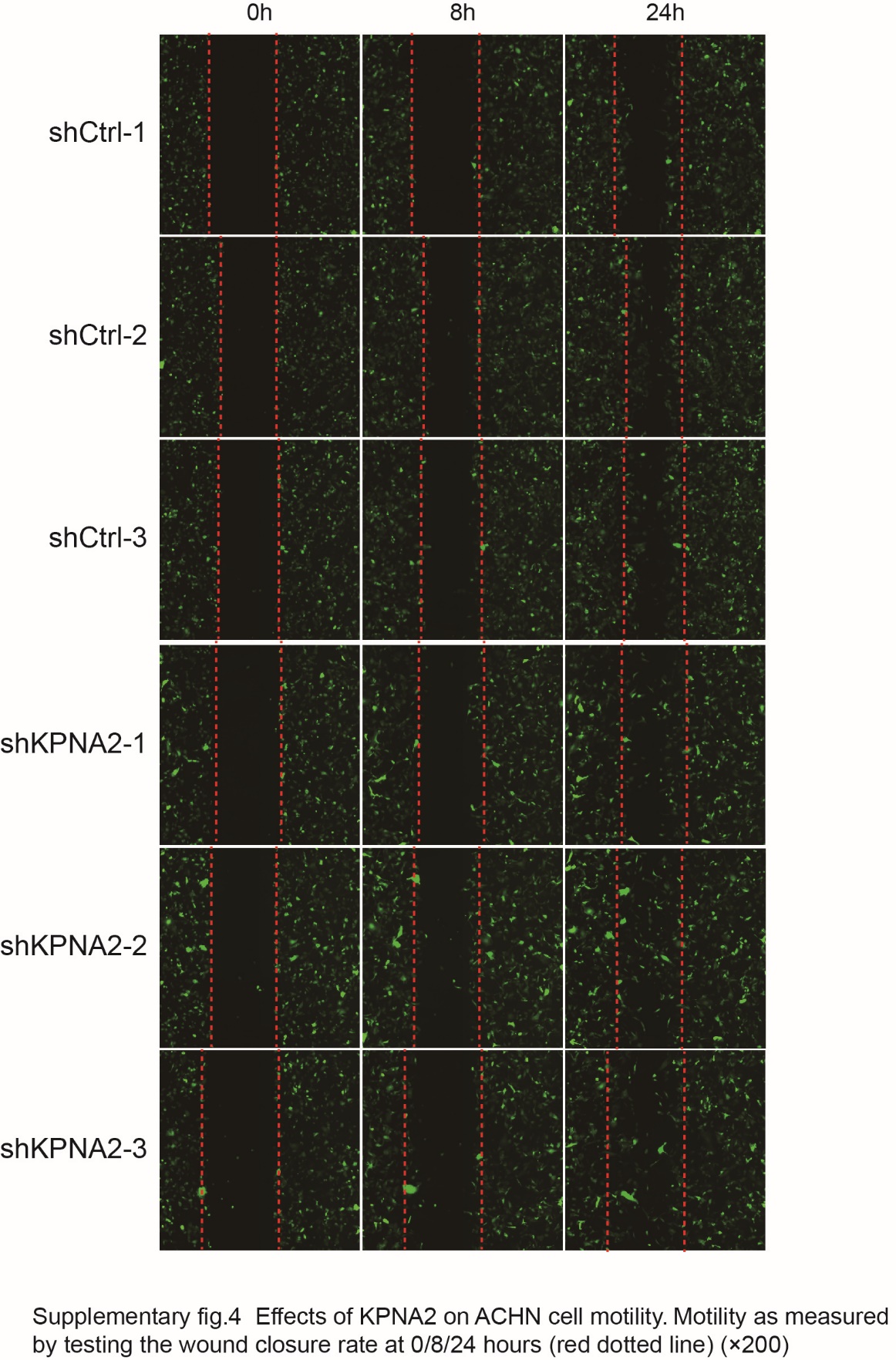


Supplementary fig.4 Effects of KPNA2 on ACHN cell motility. Motility as measured
by testing the wound closure rate at 0/8/24 hours (red dotted line) (×200).
