# Supplementary material for: KPNA2 promotes renal cell carcinoma proliferation and metastasis via NPM
Source: J Cell Mol Med. 2021 Sep 1;25(19):9255–67. doi: 10.1111/jcmm.16846 (PMC8500977; doi:10.1111/jcmm.16846)
Supplement: Supplementary file 6 — Table S1 [file JCMM-25-9255-s003.docx]

| **Supplementray table1 peptide identified in ACHN cells without KPNA2 overexpressed(NC)** | | | | | | | | | |
| --- | --- | --- | --- | --- | --- | --- | --- | --- | --- |
| Sequence | Qvality PEP | Qvality q-value | # PSMs | Master Protein Accessions | Theo. MH+ [Da] | Ions Score Mascot | Charge Mascot | DeltaM [ppm] Mascot | RT [min] Mascot |
| RQQEEEER | 0.002 | 0.000 | 1 | Q01082 | 1103.508 | 49.86 | 2 | 1.461 | 0.014 |
| SGASSSEQNNNSYETK | 0.005 | 0.000 | 1 | O60825 | 1744.726 | 34.6 | 2 | -0.704 | 18.132 |
| AGFAGDDAPR | 0.001 | 0.000 | 3 | P60709 | 976.448 | 70.46 | 2 | -0.370 | 18.798 |
| LLADQAEAR | 0.008 | 0.000 | 1 | P84098 | 986.527 | 41.27 | 2 | 0.064 | 18.309 |
| CCTESLVNR | 0.009 | 0.000 | 1 | P02768 | 1138.498 | 25.96 | 2 | 2.268 | 18.555 |
| SAVTTVVNPK | 0.003 | 0.000 | 1 | P05556 | 1015.578 | 48.65 | 2 | 2.582 | 18.595 |
| QLETLGQEK | 0.003 | 0.000 | 1 | P05787 | 1045.552 | 45.49 | 2 | 2.569 | 18.637 |
| EVYQQQQYGSGGR | 0.000 | 0.000 | 1 | Q99729 | 1499.687 | 49.25 | 2 | 2.007 | 18.671 |
| SQLLGSAHEVQR | 0.000 | 0.000 | 2 | Q13813 | 1324.697 | 72.1 | 2 | 0.950 | 18.701 |
| LVSDGNINSDR | 0.002 | 0.000 | 1 | Q01082 | 1189.581 | 49.92 | 2 | -1.658 | 18.829 |
| TKFETEQALR | 0.007 | 0.000 | 1 | P08727; Q04695 | 1222.643 | 26.19 | 2 | -0.500 | 18.831 |
| GSCGIGGGIGGGSSR | 0.010 | 0.000 | 1 | P08779 | 1278.586 | 25.97 | 2 | 4.658 | 18.924 |
| SQYEQLAEQNRK | 0.005 | 0.000 | 2 | P13645 | 1493.734 | 42.48 | 2 | -2.448 | 18.035 |
| AEAESLYQSK | 0.006 | 0.000 | 1 | P04264 | 1125.542 | 62.13 | 2 | 2.096 | 18.968 |
| YEELQQTAGR | 0.001 | 0.000 | 1 | P13647 | 1194.575 | 54.1 | 2 | 1.990 | 19.017 |
| ATTATMATSGSAR | 0.012 | 0.000 | 1 | P38919 | 1283.590 | 50 | 2 | 1.078 | 19.024 |
| EMEAELEDER | 0.005 | 0.000 | 1 | P35579 | 1266.515 | 43.27 | 2 | 3.077 | 19.055 |
| AENQSTTLPGPGR | 0.001 | 0.000 | 1 | Q7Z353 | 1327.660 | 49.69 | 2 | 2.493 | 19.103 |
| IQNAGGSVMIQR | 0.000 | 0.000 | 1 | O75688 | 1289.663 | 65.43 | 2 | 3.003 | 19.246 |
| TAVCDIPPR | 0.010 | 0.000 | 4 | P07437 | 1028.519 | 33.8 | 2 | 2.130 | 24.228 |
| QVSDDLTER | 0.013 | 0.000 | 1 | P35232 | 1062.506 | 60.12 | 2 | -0.614 | 19.321 |
| GTQGAEEVLR | 0.000 | 0.000 | 1 | Q15149 | 1059.543 | 75.9 | 2 | -0.939 | 19.361 |
| VTLTSEEEAR | 0.003 | 0.000 | 1 | P00338 | 1134.564 | 40.85 | 2 | -0.162 | 19.362 |
| EANEILQR | 0.011 | 0.000 | 1 | P12268 | 972.511 | 52.01 | 2 | 1.409 | 19.375 |
| QGGGGGGGSVPGIER | 0.000 | 0.000 | 1 | P52272 | 1284.629 | 71.69 | 2 | 0.645 | 19.423 |
| AQYEDIAQK | 0.004 | 0.000 | 1 | P04264 | 1065.521 | 39.08 | 2 | 1.995 | 19.003 |
| QAASSLQQASLK | 0.002 | 0.000 | 1 | P38646 | 1231.664 | 58.31 | 2 | -0.579 | 19.456 |
| DAPQDFHPDR | 0.004 | 0.000 | 1 | P21333 | 1197.528 | 41.44 | 2 | -3.940 | 18.023 |
| QDVDNASLAR | 0.006 | 0.000 | 1 | P08670 | 1088.533 | 59 | 2 | 1.169 | 17.590 |
| EQSQLTATQTR | 0.005 | 0.000 | 1 | Q6P2Q9 | 1262.634 | 35.86 | 2 | 1.412 | 15.503 |
| DSYVGDEAQSKR | 0.000 | 0.000 | 2 | P60709 | 1354.623 | 55.63 | 3 | 2.664 | 15.515 |
| FSSSSGYGGGSSR | 0.001 | 0.000 | 2 | P35527 | 1235.529 | 59.39 | 2 | 1.000 | 16.121 |
| ALVADSHPESER | 0.001 | 0.000 | 1 | Q01082 | 1310.634 | 64.92 | 2 | 2.571 | 15.662 |
| VAQGVSGAVQDK | 0.007 | 0.000 | 1 | P12268 | 1158.611 | 32.12 | 2 | 2.887 | 15.966 |
| GGSGGSYGGGGSGGGYGGGSGSR | 0.000 | 0.000 | 1 | P35527 | 1791.728 | 71.27 | 2 | -1.702 | 16.029 |
| TMQNTSDLDTAR | 0.005 | 0.000 | 1 | P14923 | 1368.606 | 24.52 | 2 | 1.442 | 16.173 |
| NKYEDEINKR | 0.003 | 0.000 | 1 | P04264; P02538; P05787; P13647 | 1308.654 | 42.91 | 3 | 2.834 | 16.291 |
| VNSLGETAER | 0.002 | 0.000 | 1 | Q13813 | 1075.538 | 52.33 | 2 | 2.895 | 16.337 |
| AAAAAAALQAK | 0.009 | 0.000 | 1 | P36578 | 956.552 | 55.71 | 2 | 1.786 | 16.495 |
| SKAEAESLYQSK | 0.001 | 0.000 | 1 | P04264 | 1340.669 | 31.12 | 2 | 0.638 | 16.502 |
| LLEGEEER | 0.011 | 0.000 | 1 | P20700; P02545 | 974.479 | 51.71 | 2 | 2.269 | 17.691 |
| AGQSAAGAAPGGGVDTR | 0.002 | 0.000 | 1 | P21333 | 1442.698 | 36.65 | 2 | 2.316 | 16.515 |
| ITITNDQNR | 0.015 | 0.000 | 1 | P11021 | 1074.554 | 36.9 | 2 | 1.313 | 16.673 |
| VAAYDKLEK | 0.012 | 0.000 | 1 | P35579 | 1036.567 | 33.04 | 2 | 2.092 | 16.820 |
| GGSGGGGSISGGGYGSGGGSGGR | 0.002 | 0.000 | 1 | P35908 | 1741.748 | 79.54 | 2 | 4.847 | 16.831 |
| VAPAQPSEEGPGR | 0.000 | 0.000 | 1 | P23588 | 1294.639 | 56.38 | 2 | 1.315 | 16.865 |
| SGGGGGGGGCGGGGGVSSLR | 0.000 | 0.000 | 1 | P13645 | 1549.677 | 89.75 | 2 | 2.114 | 17.028 |
| EAGEQGDIEPR | 0.003 | 0.000 | 1 | Q13283 | 1200.549 | 46.06 | 2 | -0.001 | 17.073 |
| VTDALNATR | 0.005 | 0.000 | 1 | P10809 | 960.511 | 53.25 | 2 | -0.671 | 17.185 |
| LQAEEVAQQK | 0.015 | 0.000 | 1 | Q15149 | 1143.600 | 24.7 | 2 | 1.034 | 17.314 |
| DSYVGDEAQSK | 0.004 | 0.000 | 1 | P60709 | 1198.522 | 41.15 | 2 | 1.449 | 17.393 |
| TTTAAAVASTGPSSR | 0.000 | 0.000 | 1 | P27816 | 1377.697 | 57.21 | 2 | -2.674 | 17.403 |
| SDEGQLSPATR | 0.003 | 0.000 | 1 | Q15149 | 1160.554 | 55.79 | 2 | -0.491 | 17.542 |
| SGGGGGGGLGSGGSIR | 0.003 | 0.000 | 1 | P35527 | 1232.598 | 43.02 | 2 | -3.843 | 16.640 |
| EVATNSELVQSGK | 0.000 | 0.000 | 1 | Q04695 | 1361.691 | 42.08 | 2 | 0.464 | 19.465 |
| AQLEPVASPAK | 0.006 | 0.000 | 2 | Q15149 | 1110.615 | 30.08 | 2 | -1.490 | 19.614 |
| NDNDTFTVK | 0.013 | 0.000 | 1 | P21333; Q14315 | 1053.485 | 32.92 | 2 | 3.185 | 19.617 |
| VVTDTDETELAR | 0.002 | 0.000 | 1 | P05198 | 1348.659 | 34.95 | 2 | -3.138 | 22.551 |
| LASYLDKVR | 0.006 | 0.000 | 2 | P08727; P13645; Q04695; P08779 | 1064.610 | 33.36 | 2 | -0.421 | 22.595 |
| AAYEAELGDAR | 0.002 | 0.000 | 1 | P02545 | 1165.548 | 63 | 2 | 2.195 | 23.240 |
| GYSFTTTAER | 0.010 | 0.000 | 1 | P60709 | 1132.527 | 13.99 | 2 | -0.454 | 23.244 |
| YSQYQQAIYK | 0.010 | 0.000 | 1 | O14744 | 1291.632 | 29.47 | 2 | 0.687 | 23.262 |
| VLETAEDIQER | 0.000 | 0.000 | 1 | Q13813 | 1302.654 | 74.78 | 2 | -0.636 | 23.321 |
| FADLSEAANR | 0.002 | 0.000 | 1 | P08670 | 1093.527 | 50.83 | 2 | 2.127 | 23.745 |
| EDQTEYLEER | 0.001 | 0.000 | 1 | P07900; P08238 | 1311.570 | 43 | 2 | 2.759 | 23.836 |
| YEELQVTAGR | 0.005 | 0.000 | 1 | P02538 | 1165.585 | 44.2 | 2 | 2.607 | 23.872 |
| ALGQNPTNAEVLK | 0.001 | 0.000 | 1 | P14649 | 1354.733 | 53.86 | 2 | 0.334 | 24.027 |
| EKQPPIDNIIR | 0.010 | 0.000 | 1 | P52292 | 1322.743 | 34.02 | 3 | 4.525 | 24.268 |
| EITALAPSTMK | 0.006 | 0.000 | 1 | P60709 | 1177.613 | 32.38 | 2 | -0.391 | 21.982 |
| LFGAAEVQR | 0.002 | 0.000 | 1 | Q13813 | 990.537 | 70.81 | 2 | 2.181 | 24.643 |
| IGGIGTVPVGR | 0.009 | 0.000 | 1 | Q05639 | 1025.610 | 40.47 | 2 | 2.808 | 25.841 |
| SSEEIESAFR | 0.002 | 0.000 | 1 | Q13813 | 1154.532 | 52.8 | 2 | 1.999 | 26.128 |
| IVLQIDNAR | 0.013 | 0.000 | 1 | P08727; P05783 | 1041.605 | 51.58 | 2 | 1.201 | 26.299 |
| FLEQQNQVLQTK | 0.002 | 0.000 | 2 | P35908; P04264 | 1475.785 | 43.26 | 2 | -3.601 | 26.312 |
| TNAENEFVTIK | 0.008 | 0.000 | 1 | P04264 | 1265.637 | 54.46 | 2 | 0.423 | 26.753 |
| FSGSGSGTDFTLK | 0.000 | 0.000 | 2 | A0A087WW87 | 1303.616 | 66.42 | 2 | 4.237 | 27.743 |
| DVNAAIATIK | 0.010 | 0.000 | 1 | P68363 | 1015.578 | 44.35 | 2 | 1.741 | 27.936 |
| TLLDIDNTR | 0.003 | 0.000 | 1 | P35527 | 1060.563 | 52.23 | 2 | 1.694 | 28.516 |
| VLDELTLTK | 0.001 | 0.000 | 1 | P13645 | 1031.598 | 48.35 | 2 | 4.271 | 29.748 |
| VPLVAPEDLR | 0.008 | 0.000 | 1 | O14744 | 1108.636 | 52.63 | 2 | 2.266 | 29.966 |
| ITALDEFATK | 0.005 | 0.000 | 1 | Q13813 | 1108.588 | 31.78 | 2 | -0.256 | 30.932 |
| ALEEANADLEVK | 0.001 | 0.000 | 1 | P08779 | 1301.658 | 43.25 | 2 | 1.528 | 25.291 |
| EAESSPFVER | 0.005 | 0.000 | 1 | P14625 | 1150.537 | 44.66 | 2 | 1.617 | 21.861 |
| STSSFSCLSR | 0.002 | 0.000 | 1 | P35908 | 1131.510 | 43.51 | 2 | 2.622 | 21.850 |
| SLVGLGGTK | 0.007 | 0.000 | 1 | P35908 | 831.493 | 60.63 | 2 | 2.437 | 21.712 |
| NTTGVTEEALK | 0.006 | 0.000 | 1 | Q13813 | 1162.595 | 32.05 | 2 | 0.849 | 19.724 |
| SLLEGEGSSGGGGR | 0.002 | 0.000 | 2 | P13645 | 1262.597 | 51.57 | 2 | 3.449 | 20.281 |
| QLEEAEEEATR | 0.005 | 0.000 | 1 | P35580 | 1304.596 | 41.08 | 2 | 3.663 | 19.792 |
| SNVSDAVAQSTR | 0.002 | 0.000 | 1 | P60174 | 1234.602 | 49.73 | 2 | 1.485 | 19.848 |
| AAMAVGGAGGSR | 0.002 | 0.000 | 1 | O14744 | 1062.500 | 58.03 | 2 | 1.276 | 19.867 |
| GAFSSVSMSGGAGR | 0.001 | 0.000 | 1 | P19013 | 1286.579 | 60.12 | 2 | 4.094 | 19.909 |
| VEIIANDQGNR | 0.004 | 0.000 | 4 | P11142; P11021 | 1228.628 | 42.74 | 2 | 1.738 | 20.106 |
| AQYEDIANR | 0.010 | 0.000 | 1 | P05787 | 1079.512 | 45.46 | 2 | 2.178 | 20.181 |
| LATNTSAPDLK | 0.009 | 0.000 | 1 | P27816 | 1130.605 | 41.83 | 2 | 2.890 | 20.218 |
| LQLEATER | 0.014 | 0.000 | 1 | Q15149 | 959.516 | 47.93 | 2 | 2.837 | 20.331 |
| TNQELQEINR | 0.004 | 0.000 | 1 | A6NMY6 | 1244.623 | 39.25 | 2 | 4.134 | 20.438 |
| VIGSGCNLDSAR | 0.000 | 0.000 | 1 | P00338 | 1248.600 | 80.03 | 2 | 3.254 | 20.503 |
| DQNTVETLQR | 0.006 | 0.000 | 1 | Q01082 | 1203.596 | 36.42 | 2 | 4.730 | 20.536 |
| AFEEDQVAGR | 0.008 | 0.000 | 1 | O43818 | 1121.522 | 35.86 | 2 | 5.193 | 20.602 |
| AATASAGAGGIDGKPR | 0.008 | 0.000 | 1 | Q02978 | 1441.739 | 38.12 | 2 | 6.552 | 20.609 |
| NNASTDYDLSDK | 0.001 | 0.000 | 1 | P39023 | 1342.576 | 36.68 | 2 | 2.638 | 20.619 |
| ELEDATETADAMNR | 0.000 | 0.000 | 1 | P35579 | 1581.670 | 38.03 | 2 | 1.015 | 20.752 |
| YENEVALR | 0.013 | 0.000 | 1 | P13645 | 993.500 | 45.92 | 2 | -0.368 | 20.822 |
| YIDQEELNK | 0.003 | 0.000 | 1 | P07900; P08238 | 1151.558 | 33.74 | 2 | 0.649 | 20.853 |
| AGELTEDEVER | 0.007 | 0.000 | 1 | P62269 | 1247.575 | 42.67 | 2 | -1.665 | 21.252 |
| IYIDSNNNPER | 0.009 | 0.000 | 1 | Q00610 | 1334.634 | 30.61 | 2 | 2.616 | 21.261 |
| VAVVNQIAR | 0.006 | 0.000 | 1 | Q01082 | 969.584 | 55.82 | 2 | 1.428 | 21.339 |
| ASITALEAK | 0.004 | 0.000 | 1 | P35579 | 903.515 | 58.64 | 2 | 0.406 | 21.378 |
| VLDNAIETEK | 0.003 | 0.000 | 1 | Q01082 | 1131.589 | 43.86 | 2 | 1.910 | 21.448 |
| SQYEQLAEQNR | 0.001 | 0.000 | 1 | P13645 | 1365.639 | 43.36 | 2 | 1.160 | 21.551 |
| ATCAPQHGAPGPGPADASK | 0.000 | 0.000 | 1 | P21333 | 1789.829 | 37.97 | 3 | 4.933 | 15.056 |
| SCCSCCPVGCAK | 0.000 | 0.000 | 1 | P13640 | 1445.510 | 52.99 | 2 | 2.249 | 14.959 |
| ASREEILAQAK | 0.002 | 0.000 | 1 | P35579 | 1215.669 | 33.69 | 2 | -1.255 | 18.112 |
| SGGGGGGGFGR | 0.004 | 0.000 | 1 | P13647 | 865.391 | 69.2 | 2 | -0.146 | 8.215 |
| STPKEDDSSASTSQSTR | 0.004 | 0.000 | 3 | P23588 | 1783.794 | 37.61 | 3 | 2.456 | 8.388 |
| ATGDETGAKVER | 0.000 | 0.000 | 2 | P61247 | 1233.607 | 56.29 | 2 | -0.584 | 7.993 |
| QLAEGTAQQR | 0.002 | 0.000 | 1 | Q15149 | 1101.565 | 64.57 | 2 | 1.804 | 12.179 |
| GPAGPSGPAGK | 0.006 | 0.000 | 1 | P08123 | 895.463 | 39.03 | 2 | 1.814 | 8.234 |
| NAEQYKDQADK | 0.000 | 0.000 | 1 | P35579 | 1309.602 | 46.3 | 2 | 4.172 | 8.379 |
| LAKEQEQAQK | 0.002 | 0.000 | 2 | Q6P3W7 | 1172.627 | 45.82 | 2 | 6.891 | 3.209 |
| ESDGASDEAEESGSQGK | 0.000 | 0.000 | 1 | O75688 | 1682.662 | 56.24 | 2 | 2.266 | 13.364 |
| KGDSSAEELK | 0.001 | 0.000 | 1 | P26373 | 1063.527 | 32.5 | 2 | 1.323 | 6.709 |
| GADGSPGKDGVR | 0.005 | 0.000 | 2 | P02452 | 1115.544 | 64.96 | 2 | 0.453 | 4.903 |
| RQEENDKLR | 0.011 | 0.000 | 1 | Q13813 | 1187.613 | 30.9 | 3 | 0.909 | 4.134 |
| GSGTAEVELKK | 0.002 | 0.000 | 1 | P14618 | 1118.605 | 62.73 | 2 | 4.012 | 13.344 |
| ALTQTGGPHVK | 0.014 | 0.000 | 1 | P21333 | 1108.611 | 22.11 | 2 | 1.280 | 13.101 |
| SQSSDTEQQSPTSGGGK | 0.000 | 0.000 | 2 | P23588 | 1680.731 | 111.19 | 2 | 0.819 | 8.961 |
| KYEDEINKR | 0.006 | 0.000 | 1 | P35908 | 1194.611 | 28.33 | 3 | 2.405 | 13.082 |
| YEDEINKR | 0.014 | 0.000 | 2 | P35908; P04264; P02538; P05787; P13647 | 1066.516 | 30.41 | 2 | 2.213 | 12.930 |
| GACAGSEDAVK | 0.007 | 0.000 | 1 | Q13813 | 1064.468 | 27.39 | 2 | 2.752 | 9.060 |
| EGQEDQGLTK | 0.005 | 0.000 | 1 | P26599 | 1104.517 | 55.12 | 2 | 1.670 | 12.926 |
| VLENAEGAR | 0.008 | 0.000 | 1 | P38646 | 958.495 | 51.66 | 2 | 1.201 | 14.793 |
| QASEGPLK | 0.005 | 0.000 | 1 | P04406 | 829.441 | 66 | 2 | 1.674 | 9.787 |
| QVHPDTGISSK | 0.013 | 0.000 | 1 | Q96A08 | 1168.596 | 54.33 | 3 | -0.105 | 12.492 |
| FSSSGGGGGGGR | 0.012 | 0.000 | 1 | P35527 | 982.434 | 58.27 | 2 | -1.113 | 6.264 |
| SRTGSESSQTGTSTTSSR | 0.000 | 0.000 | 2 | P23588 | 1816.827 | 67.6 | 2 | -1.637 | 6.707 |
| QSSEAEIQAK | 0.001 | 0.000 | 1 | Q15149 | 1090.537 | 64.42 | 2 | 1.706 | 12.315 |
| STMQELNSR | 0.006 | 0.000 | 1 | P35527 | 1081.494 | 48.19 | 2 | 2.089 | 10.309 |
| QLAEAHAQAK | 0.000 | 0.000 | 1 | Q15149 | 1066.564 | 64.54 | 2 | -1.460 | 6.695 |
| RQHEAEEGVR | 0.006 | 0.000 | 1 | Q15149 | 1210.592 | 33.22 | 3 | 2.215 | 5.238 |
| KGEITGEVR | 0.006 | 0.000 | 1 | P21333 | 988.542 | 52.4 | 2 | 1.582 | 12.314 |
| RQVDQLTNDK | 0.001 | 0.000 | 1 | P08670 | 1216.628 | 47.47 | 2 | -0.954 | 13.664 |
| TDQQAEAR | 0.010 | 0.000 | 1 | P02585 | 960.438 | 71.4 | 2 | 0.491 | 13.685 |
| DLQGRDEQSEEK | 0.008 | 0.000 | 1 | P35579 | 1433.650 | 22.16 | 2 | 2.487 | 11.335 |
| GSGGGSSGGSIGGR | 0.000 | 0.000 | 3 | P04264 | 1092.503 | 92.75 | 2 | -2.332 | 6.884 |
| ADEVAPAKK | 0.015 | 0.000 | 1 | P53396 | 928.510 | 33.25 | 2 | 1.174 | 2.251 |
| VADISGDTQK | 0.008 | 0.000 | 1 | Q7KZF4 | 1033.516 | 38.87 | 2 | 0.954 | 14.459 |
| EDDSSASTSQSTR | 0.000 | 0.000 | 1 | P23588 | 1370.567 | 48.27 | 2 | -1.102 | 7.146 |
| TGSESSQTGTSTTSSR | 0.000 | 0.000 | 2 | P23588 | 1573.694 | 77.97 | 2 | 0.102 | 7.021 |
| QIEAQEKPR | 0.010 | 0.000 | 1 | Q01082 | 1098.590 | 48.13 | 2 | -2.279 | 7.198 |
| IVTDRETGSSK | 0.000 | 0.000 | 1 | P19338 | 1192.617 | 84.14 | 2 | -2.916 | 7.241 |
| QVDQLTNDKAR | 0.001 | 0.000 | 2 | P08670 | 1287.665 | 53.52 | 2 | 0.327 | 13.834 |
| SSSSGSVGESSSK | 0.003 | 0.000 | 1 | P13645 | 1185.523 | 58.73 | 2 | 1.159 | 2.023 |
| TQEKEQIK | 0.009 | 0.000 | 1 | P05787 | 1003.542 | 32.66 | 2 | 0.310 | 1.946 |
| QEMQEVQSSR | 0.002 | 0.000 | 1 | P22626 | 1237.548 | 54.99 | 2 | 1.706 | 7.518 |
| QVAQQEAQR | 0.008 | 0.000 | 1 | Q99623 | 1057.538 | 54.35 | 2 | 2.658 | 6.971 |
| RAAEEAEEAR | 0.006 | 0.000 | 1 | Q15149 | 1131.539 | 22.94 | 2 | 2.244 | 6.949 |
| GGSISGGGYGSGGGK | 0.001 | 0.000 | 1 | P35908 | 1197.549 | 49.23 | 2 | 4.205 | 14.617 |
| ASSHSSQTQGGGSVTK | 0.010 | 0.000 | 1 | P02545 | 1518.714 | 29.59 | 2 | 0.076 | 2.759 |
| LQKEQEKLQR | 0.006 | 0.000 | 1 | P23588 | 1299.738 | 35.1 | 3 | 0.817 | 7.629 |
| LGDSHDLQR | 0.002 | 0.000 | 1 | Q13813 | 1040.512 | 55.54 | 2 | 2.759 | 14.226 |
| KPEENPASK | 0.002 | 0.000 | 2 | P23588 | 999.511 | 69.07 | 2 | -0.127 | 1.373 |
| AQAVSEDAGGNEGR | 0.006 | 0.000 | 1 | P55884 | 1360.609 | 34.46 | 2 | 1.832 | 13.977 |
| GPAGPSGPAGKDGR | 0.003 | 0.000 | 1 | P08123 | 1223.613 | 45.24 | 2 | -0.004 | 7.785 |
| TENSTSAPAAKPK | 0.008 | 0.000 | 1 | P07305 | 1343.680 | 28.46 | 2 | 2.610 | 13.945 |
| AEEAEAQKR | 0.005 | 0.000 | 1 | Q15149 | 1031.512 | 41.59 | 2 | 1.687 | 0.510 |
| AGGTGLER | 0.015 | 0.000 | 1 | Q14315 | 760.395 | 55.01 | 2 | 1.485 | 7.881 |
| TDSSPNQAR | 0.014 | 0.000 | 1 | P26599 | 975.449 | 40.21 | 2 | -0.741 | 2.813 |
| DLVQGEHLGR | 0.016 | 0.003 | 1 | P23458 | 1123.585 | 34.09 | 2 | 0.479 | 21.296 |
| NTVVPTKK | 0.041 | 0.003 | 1 | P11021 | 886.536 | 34.4 | 2 | 1.157 | 6.191 |
| IAPAEGPDVSER | 0.017 | 0.003 | 1 | Q9Y6M1 | 1240.617 | 31.91 | 2 | -1.524 | 20.194 |
| LAQFEPSQR | 0.031 | 0.003 | 1 | Q08211 | 1075.553 | 29.56 | 2 | 0.741 | 20.750 |
| ALELEQER | 0.039 | 0.003 | 1 | P26038 | 987.511 | 34.82 | 2 | 1.973 | 20.945 |
| EIGQSVDEVEK | 0.038 | 0.003 | 1 | Q01082 | 1232.600 | 35.77 | 2 | 2.396 | 21.209 |
| STSQGSINSPVYSR | 0.023 | 0.003 | 1 | O14639 | 1482.718 | 29 | 2 | 0.657 | 21.087 |
| GFSGLDGAK | 0.030 | 0.003 | 3 | P02452 | 851.426 | 45.16 | 2 | 0.872 | 20.909 |
| VNVGAGSHPNK | 0.022 | 0.003 | 1 | P21333 | 1079.559 | 38.58 | 2 | 6.124 | 6.993 |
| EVDEQMLNVQNK | 0.034 | 0.003 | 1 | P07437 | 1462.684 | 17.37 | 2 | 0.847 | 20.903 |
| AQQDKLNTR | 0.019 | 0.003 | 2 | Q01082 | 1073.570 | 47.38 | 2 | -1.635 | 6.778 |
| AKQTLENER | 0.022 | 0.003 | 1 | P35579 | 1088.569 | 11.8 | 2 | 4.638 | 6.822 |
| VNFAMNVGK | 0.017 | 0.003 | 1 | P14618 | 995.498 | 25.94 | 2 | 2.364 | 20.639 |
| AGVAPLQVK | 0.034 | 0.003 | 1 | P21333 | 882.541 | 35.84 | 2 | 1.486 | 20.721 |
| SAINEVVTR | 0.032 | 0.003 | 1 | P62899 | 988.542 | 38.18 | 2 | 1.953 | 21.238 |
| QLAEEDAAR | 0.043 | 0.003 | 1 | Q15149 | 1002.485 | 37.87 | 2 | -0.274 | 14.839 |
| ISEQSDAK | 0.037 | 0.003 | 1 | P25705 | 877.426 | 46.39 | 2 | 1.371 | 4.419 |
| QLIVGVNK | 0.037 | 0.003 | 1 | Q05639 | 870.541 | 39.87 | 2 | 0.315 | 21.542 |
| ELISNSSDALDK | 0.040 | 0.003 | 1 | P07900 | 1291.638 | 27.79 | 2 | 3.229 | 24.457 |
| ISVYYNEATGGK | 0.033 | 0.003 | 1 | P07437 | 1301.637 | 24.8 | 2 | 3.319 | 24.735 |
| AEGGAADLDTQR | 0.036 | 0.003 | 1 | Q9Y4E8 | 1245.571 | 33.71 | 2 | 2.172 | 25.168 |
| AQIFANTVDNAR | 0.032 | 0.003 | 1 | P05783 | 1319.670 | 26.96 | 2 | 2.571 | 25.190 |
| QEYEQLIAK | 0.023 | 0.003 | 1 | P35527 | 1121.584 | 41.71 | 2 | 3.656 | 25.221 |
| LLVGSEDYGR | 0.034 | 0.003 | 1 | Q13813 | 1108.563 | 41.94 | 2 | 1.511 | 25.507 |
| LQAEIEGLK | 0.016 | 0.003 | 1 | P05787 | 1000.567 | 30.84 | 2 | 2.167 | 25.524 |
| VELQELNDR | 0.025 | 0.003 | 1 | P08670 | 1115.569 | 42.84 | 2 | 4.169 | 24.354 |
| IEISELNR | 0.038 | 0.003 | 1 | P35908; P04264 | 973.531 | 36.24 | 2 | 1.391 | 25.604 |
| STDEVDSK | 0.032 | 0.003 | 1 | Q01082 | 880.389 | 39.12 | 2 | 0.438 | 2.470 |
| SIYYITGESK | 0.026 | 0.003 | 1 | P08238 | 1160.583 | 38.71 | 2 | 2.139 | 26.237 |
| FDQLLAEEK | 0.018 | 0.003 | 1 | P35580; P35579 | 1092.557 | 35.83 | 2 | 0.121 | 26.257 |
| GDYPLEAVR | 0.037 | 0.003 | 1 | P14618 | 1019.516 | 38.03 | 2 | -1.281 | 26.287 |
| NDLAVVDVR | 0.019 | 0.003 | 1 | P19338 | 1000.542 | 57.7 | 2 | -0.328 | 26.309 |
| AGAHLQGGAK | 0.034 | 0.003 | 1 | P04406 | 909.490 | 29.22 | 2 | 0.815 | 2.187 |
| LGESQTLQQFSR | 0.031 | 0.003 | 1 | Q13813 | 1393.707 | 34.11 | 2 | 3.459 | 27.747 |
| AIETTDIISR | 0.017 | 0.003 | 1 | Q96HS1 | 1118.605 | 44.25 | 2 | 3.139 | 25.826 |
| SDGFSIETCK | 0.030 | 0.003 | 1 | P17655 | 1143.499 | 26.52 | 2 | 3.878 | 24.299 |
| VGTECGNQK | 0.038 | 0.003 | 1 | P21333 | 992.447 | 37.74 | 2 | 2.163 | 3.004 |
| ETWLSENQR | 0.037 | 0.003 | 1 | Q01082 | 1162.549 | 29.65 | 2 | 1.388 | 24.047 |
| QTVAVGVIK | 0.019 | 0.003 | 1 | Q05639 | 914.567 | 62.35 | 2 | 2.535 | 21.604 |
| VHIEIGPDGR | 0.042 | 0.003 | 1 | P31943 | 1092.580 | 36.5 | 2 | 1.680 | 21.786 |
| KEEELQAALAR | 0.017 | 0.003 | 1 | P35579 | 1257.680 | 43.24 | 2 | 2.034 | 21.787 |
| ASDVHEVR | 0.039 | 0.003 | 1 | P14618 | 912.453 | 36.02 | 2 | 0.854 | 5.104 |
| QLYEEEIR | 0.037 | 0.003 | 1 | P05787 | 1079.537 | 39.87 | 2 | 4.999 | 23.906 |
| LTVLSEER | 0.029 | 0.003 | 1 | Q13813 | 946.520 | 31.23 | 2 | 0.178 | 22.048 |
| LENEIQTYR | 0.027 | 0.003 | 1 | P13645 | 1165.585 | 18.42 | 2 | 0.512 | 22.489 |
| TVSPALISR | 0.030 | 0.003 | 1 | Q86VP6 | 943.557 | 30.7 | 2 | 6.025 | 22.717 |
| LITDLQDQNQK | 0.019 | 0.003 | 1 | P33176 | 1315.685 | 25.63 | 2 | -0.643 | 22.813 |
| ALAAAGYDVEK | 0.027 | 0.003 | 1 | P16402 | 1107.568 | 30.01 | 2 | -0.242 | 22.859 |
| LEVNLQAMK | 0.023 | 0.003 | 1 | P35579 | 1061.566 | 28.39 | 2 | -0.452 | 23.025 |
| DVNQQEFVR | 0.042 | 0.003 | 1 | P39019 | 1134.554 | 9.66 | 2 | -3.167 | 23.086 |
| TAAAAAEHSQR | 0.021 | 0.003 | 1 | O43242 | 1112.544 | 33.75 | 2 | -1.273 | 3.875 |
| LESLNIQR | 0.032 | 0.003 | 1 | Q14152 | 972.547 | 24.79 | 2 | -0.169 | 23.431 |
| EEQFNSTFR | 0.036 | 0.003 | 1 | P01859 | 1157.522 | 32.78 | 2 | -0.452 | 23.654 |
| STDEVDSKR | 0.028 | 0.003 | 1 | Q01082 | 1036.491 | 35.13 | 2 | 1.868 | 3.155 |
| LVQEQGSHSK | 0.033 | 0.003 | 1 | P35580 | 1112.569 | 28.48 | 2 | 1.356 | 3.052 |
| LIEVDDER | 0.016 | 0.003 | 1 | P62753 | 988.495 | 44.87 | 2 | 1.841 | 21.462 |
| AAVAGEDGR | 0.044 | 0.003 | 1 | P07910 | 845.411 | 30.23 | 2 | 2.033 | 7.224 |
| TLLEGEESR | 0.025 | 0.003 | 2 | P04264 | 1033.516 | 44.64 | 2 | 4.497 | 20.617 |
| SLQSVAEER | 0.017 | 0.003 | 1 | P61313 | 1018.516 | 41.31 | 2 | 1.119 | 19.987 |
| SESPKEPEQLR | 0.029 | 0.003 | 1 | P09651 | 1299.654 | 22.35 | 3 | 2.477 | 15.909 |
| GTNESLER | 0.037 | 0.003 | 1 | P08670 | 905.432 | 57.2 | 2 | 1.684 | 9.788 |
| ATDKSFVEK | 0.030 | 0.003 | 1 | P35579 | 1024.531 | 32.65 | 2 | 3.317 | 9.894 |
| EASLGEASK | 0.041 | 0.003 | 1 | Q01082 | 891.442 | 42.83 | 2 | 1.800 | 10.241 |
| AAQEEYVKR | 0.023 | 0.003 | 1 | P04075 | 1093.564 | 36.39 | 2 | 0.110 | 10.413 |
| AAREPNIDR | 0.030 | 0.003 | 1 | P23588 | 1041.544 | 32.33 | 2 | 2.270 | 11.253 |
| MFGGPGTASRPSSSR | 0.042 | 0.003 | 1 | P08670 | 1510.707 | 21.82 | 3 | 1.029 | 15.944 |
| LQDAEIAR | 0.030 | 0.003 | 1 | P06703 | 915.489 | 39.33 | 2 | 0.807 | 17.626 |
| AQAEQAALR | 0.036 | 0.003 | 1 | Q15149 | 957.511 | 41.25 | 2 | 2.675 | 13.522 |
| SGAQASSTPLSPTR | 0.018 | 0.003 | 1 | P02545 | 1359.686 | 35.63 | 2 | 6.048 | 18.165 |
| AEGPGLSR | 0.021 | 0.003 | 1 | P21333 | 786.410 | 49.54 | 2 | 1.637 | 13.634 |
| VGGSGVNVNAK | 0.038 | 0.003 | 1 | Q09666 | 1001.537 | 18.17 | 2 | 2.039 | 14.254 |
| STESLQANVQR | 0.030 | 0.003 | 1 | P26373 | 1232.623 | 31.46 | 2 | -0.084 | 18.542 |
| LTEDEVEK | 0.025 | 0.003 | 1 | P08590 | 962.468 | 38.02 | 2 | 0.905 | 16.170 |
| LSVAAQEAAR | 0.019 | 0.003 | 1 | Q15149 | 1015.553 | 38.47 | 2 | 0.664 | 18.033 |
| LGQEATVGK | 0.016 | 0.003 | 1 | P53396 | 902.494 | 44.95 | 2 | -2.349 | 13.761 |
| EGQTICVR | 0.033 | 0.003 | 1 | O14744 | 962.472 | 57.1 | 2 | 1.466 | 16.748 |
| SFQQSSLSR | 0.032 | 0.003 | 1 | P43243 | 1039.517 | 36.59 | 2 | -1.222 | 18.329 |
| ASGPGLER | 0.025 | 0.003 | 1 | Q14315 | 786.410 | 46.95 | 2 | 0.473 | 12.371 |
| GVVGPQGAR | 0.029 | 0.003 | 1 | P08123 | 840.469 | 44.55 | 2 | -1.684 | 12.819 |
| TVGVEPAADGK | 0.026 | 0.003 | 1 | P46779 | 1043.537 | 34.22 | 2 | 1.896 | 15.999 |
| EVFTSSSSSSSR | 0.040 | 0.003 | 1 | P08779 | 1260.570 | 22.16 | 2 | -0.590 | 15.786 |
| RQVDQLTNDKAR | 0.040 | 0.003 | 1 | P08670 | 1443.766 | 29.96 | 3 | 1.272 | 13.085 |
| YSVQTADHR | 0.028 | 0.003 | 1 | Q16658 | 1076.512 | 33.67 | 2 | 0.739 | 13.706 |
| ATAVMPDGQFK | 0.033 | 0.003 | 1 | Q06830 | 1180.567 | 28.87 | 2 | 0.528 | 19.872 |
| TQDQISNIK | 0.027 | 0.003 | 1 | Q14847 | 1046.548 | 27.53 | 2 | 2.091 | 17.053 |
| RGDDSFGDK | 0.021 | 0.003 | 1 | P23588 | 996.438 | 44.12 | 2 | 0.657 | 7.353 |
| IEYAKPTR | 0.031 | 0.003 | 1 | P14866 | 977.541 | 33.36 | 2 | -4.586 | 13.461 |
| VPDVQDGVR | 0.044 | 0.003 | 1 | Q15149 | 984.511 | 32.71 | 2 | -1.770 | 19.895 |
| IQQNTFTR | 0.031 | 0.003 | 1 | P21333; Q14315 | 1007.527 | 28.86 | 2 | 0.034 | 17.303 |
| VDQLKSDQSR | 0.032 | 0.003 | 1 | P04264 | 1175.601 | 24.3 | 2 | 1.036 | 7.574 |
| LEPGGGAEAQAVR | 0.043 | 0.003 | 1 | Q14315 | 1254.644 | 34.41 | 2 | -0.459 | 19.198 |
| TASFSESR | 0.036 | 0.003 | 1 | P53396 | 884.411 | 36.54 | 2 | 2.390 | 13.360 |
| LITEDVQGK | 0.023 | 0.003 | 1 | P61247 | 1002.547 | 20.27 | 2 | 1.599 | 19.869 |
| LQAAYAGDK | 0.026 | 0.003 | 1 | Q01082 | 936.479 | 37.71 | 2 | 2.912 | 15.445 |
| TATDEAYKDPSNLQGK | 0.034 | 0.003 | 1 | Q13813 | 1737.829 | 16.27 | 3 | -1.317 | 19.908 |
| NKEEAAEYAK | 0.027 | 0.003 | 1 | P62753 | 1152.553 | 16.43 | 2 | 0.851 | 7.863 |
| VQAVVAVAR | 0.025 | 0.003 | 1 | Q01082 | 912.563 | 33.11 | 2 | 0.424 | 19.446 |
| VLADPSDDTK | 0.029 | 0.003 | 1 | Q15149 | 1060.516 | 27.08 | 2 | 1.130 | 16.961 |
| TVPVEAVTSK | 0.023 | 0.003 | 1 | Q14247 | 1030.578 | 20.22 | 2 | 5.356 | 19.433 |
| YSTSGSSGLTTGK | 0.043 | 0.003 | 1 | Q16891 | 1245.596 | 8.08 | 2 | 2.560 | 17.390 |
| TAEAELSR | 0.039 | 0.003 | 1 | Q08378 | 876.442 | 41.82 | 2 | 0.335 | 14.650 |
| QQYESVAAK | 0.034 | 0.003 | 1 | P08670 | 1023.511 | 43.89 | 2 | 2.500 | 14.544 |
| ILSSDDYGK | 0.034 | 0.003 | 1 | Q01082 | 997.484 | 27 | 2 | 0.146 | 19.300 |
| GELAIKDANAK | 0.036 | 0.003 | 1 | P05787 | 1129.621 | 36.56 | 3 | 2.454 | 15.446 |
| IECDDKGDGSCDVR | 0.030 | 0.003 | 1 | P21333; Q14315 | 1625.653 | 20.81 | 3 | 1.789 | 14.728 |
| LSPSPTSQR | 0.064 | 0.005 | 1 | P02545 | 972.511 | 38.36 | 2 | 3.354 | 13.646 |
| MTLDDFR | 0.071 | 0.005 | 1 | P35527 | 913.408 | 37.49 | 2 | 0.177 | 24.178 |
| DTGNIGQER | 0.060 | 0.005 | 1 | Q01082 | 989.465 | 26.01 | 2 | -0.818 | 13.624 |
| GSDFDCELR | 0.049 | 0.005 | 1 | P61978 | 1098.452 | 20.48 | 2 | -1.089 | 24.191 |
| VTPDTDWAR | 0.073 | 0.005 | 1 | P53396 | 1060.506 | 17.23 | 2 | 2.058 | 23.912 |
| GGSGGSYGR | 0.059 | 0.005 | 1 | P35527 | 797.354 | 38.33 | 2 | -0.193 | 2.626 |
| ASLSLIEK | 0.053 | 0.005 | 1 | P52292 | 860.509 | 29.06 | 2 | 2.927 | 24.627 |
| KGNYAER | 0.055 | 0.005 | 1 | Q96QV6 | 837.421 | 49.96 | 2 | 0.694 | 0.574 |
| LSYNTASNK | 0.068 | 0.005 | 1 | P49207 | 997.495 | 18.07 | 2 | 1.061 | 14.671 |
| SGQGAFGNMCR | 0.045 | 0.005 | 1 | P36578 | 1200.488 | 29.68 | 2 | 2.643 | 15.138 |
| LSVDYGKK | 0.064 | 0.005 | 1 | P68363 | 909.504 | 29.12 | 2 | 2.289 | 15.325 |
| FQNALLVR | 0.069 | 0.005 | 1 | P02768 | 960.563 | 21.83 | 2 | 2.310 | 27.107 |
| VGSAAQTR | 0.058 | 0.005 | 1 | P25705 | 789.421 | 29.73 | 2 | 1.123 | 2.198 |
| YKAEDEKQR | 0.054 | 0.005 | 1 | P11142 | 1166.580 | 30.13 | 2 | 1.445 | 2.327 |
| STVDAPTAAGR | 0.045 | 0.005 | 1 | Q99832 | 1045.527 | 17.9 | 2 | -2.796 | 15.806 |
| YEELQVTAGK | 0.069 | 0.005 | 1 |  | 1137.579 | 22.65 | 2 | 3.676 | 26.028 |
| YEELQSLAGK | 0.069 | 0.005 | 1 | P05787 | 1137.579 | 22.65 | 2 | 3.676 | 26.028 |
| ETPPPLVPPAAR | 0.056 | 0.005 | 1 | Q9BQA1 | 1244.700 | 34.97 | 2 | 4.026 | 25.789 |
| LPREPSNPER | 0.061 | 0.005 | 1 | P23588 | 1194.623 | 15.61 | 3 | 2.199 | 16.011 |
| RQLETLGQEK | 0.053 | 0.005 | 1 | P05787 | 1201.654 | 28.04 | 2 | 2.510 | 16.139 |
| IEEELGSK | 0.056 | 0.005 | 1 | P06733 | 904.462 | 29.47 | 2 | 2.837 | 16.359 |
| ILDQSEAEK | 0.054 | 0.005 | 1 | Q14764 | 1032.521 | 8.1 | 2 | 5.693 | 16.420 |
| GALALEEKR | 0.051 | 0.005 | 1 | P35579 | 986.563 | 38.61 | 2 | 2.406 | 16.498 |
| QAQEEAER | 0.048 | 0.005 | 1 | Q15149 | 960.438 | 45.3 | 2 | 0.554 | 3.180 |
| LLLPGELAK | 0.058 | 0.005 | 1 | Q96A08 | 953.603 | 31.07 | 2 | 5.909 | 31.616 |
| AQQQAEAER | 0.053 | 0.005 | 1 | Q15149 | 1030.491 | 20.95 | 2 | -0.429 | 3.979 |
| ILGPGLNK | 0.063 | 0.005 | 1 | P62906 | 811.504 | 27.33 | 2 | 1.772 | 21.631 |
| DGEAGAQGPPGPAGPAGER | 0.056 | 0.005 | 1 | P02452 | 1690.778 | 27.4 | 2 | -0.981 | 20.821 |
| GSEGPQGVR | 0.049 | 0.005 | 1 | P02452 | 886.438 | 45.6 | 2 | 2.491 | 8.432 |
| VGFAEAAR | 0.062 | 0.005 | 1 | P07339 | 820.431 | 39.53 | 2 | 2.110 | 19.116 |
| SLSAQEEK | 0.058 | 0.005 | 1 | Q13813 | 891.442 | 29.42 | 2 | 0.636 | 7.615 |
| NSQGEEVAQR | 0.053 | 0.005 | 1 | P20700 | 1117.523 | 23.58 | 2 | 5.025 | 8.628 |
| EKQLAAENR | 0.051 | 0.005 | 1 | P35579 | 1058.559 | 37.36 | 2 | -0.473 | 6.357 |
| QGTEIDGR | 0.059 | 0.005 | 1 | P19338 | 875.422 | 55.61 | 2 | 1.399 | 11.124 |
| DIDIHEVR | 0.072 | 0.005 | 1 | Q00839 | 996.511 | 19.7 | 2 | 3.151 | 21.379 |
| INHEGEVNR | 0.060 | 0.005 | 1 | Q09028 | 1067.523 | 22.44 | 2 | -0.774 | 6.886 |
| QINDIQLSR | 0.045 | 0.005 | 1 | Q13347 | 1086.590 | 28.56 | 2 | -0.731 | 23.756 |
| TKQDEVNAAWQR | 0.063 | 0.005 | 1 | Q13813 | 1445.713 | 14.25 | 3 | -0.716 | 19.773 |
| LAPEYEAAATR | 0.048 | 0.005 | 1 | P30101 | 1191.600 | 25.98 | 2 | 0.480 | 21.507 |
| TYETTLEK | 0.052 | 0.005 | 1 | P02768 | 984.488 | 9.79 | 2 | 1.211 | 18.561 |
| GSSGLGGGSSR | 0.058 | 0.005 | 1 | Q04695 | 921.438 | 32.35 | 2 | 1.606 | 5.493 |
| LTVDEAVR | 0.055 | 0.005 | 1 | Q15149 | 902.494 | 40.63 | 2 | 2.588 | 21.594 |
| SEITELR | 0.068 | 0.005 | 1 | P13645 | 847.452 | 45.66 | 2 | 3.432 | 20.531 |
| SLLDACESR | 0.045 | 0.005 | 1 | Q01082 | 1050.488 | 27.99 | 2 | -4.633 | 23.231 |
| FSPGAPGGSGSQPNQK | 0.066 | 0.005 | 1 | Q15942 | 1515.719 | 15.35 | 2 | -0.302 | 17.580 |
| SLVSVTK | 0.048 | 0.005 | 1 | P08238 | 733.445 | 52.03 | 2 | 1.152 | 17.727 |
| NMQDMVEDYR | 0.071 | 0.005 | 1 | P04264 | 1332.519 | 20.53 | 2 | 0.992 | 20.001 |
| YDPEGDNTGEQVAVK | 0.050 | 0.005 | 1 | P23458 | 1621.734 | 21.98 | 2 | 1.377 | 22.664 |
| VNEVNQFAAK | 0.063 | 0.005 | 1 | Q13813 | 1119.579 | 28.18 | 2 | 0.848 | 20.272 |
| LKGLALQR | 0.070 | 0.005 | 1 | Q13813 | 898.583 | 30.71 | 2 | 0.925 | 19.310 |
| SGGGYGGDR | 0.064 | 0.005 | 1 | Q92804 | 825.349 | 27.06 | 2 | -4.895 | 4.639 |
| AQAEVEGLGK | 0.070 | 0.005 | 1 | Q15149 | 1001.526 | 18.66 | 2 | 1.616 | 18.249 |
| NHEEEISTLR | 0.053 | 0.005 | 1 | P08727 | 1227.596 | 36.4 | 2 | -1.428 | 18.446 |
| QQPDTEAVLNGK | 0.067 | 0.005 | 1 | P54105 | 1299.654 | 20.67 | 2 | 0.890 | 21.713 |
| FLQEEAEK | 0.052 | 0.005 | 1 | Q15149 | 993.489 | 22.06 | 2 | 1.847 | 18.479 |
| TKTEISEMNR | 0.065 | 0.005 | 1 | P05787 | 1224.589 | 15.42 | 2 | 0.031 | 7.345 |
| DDRGPPQRPK | 0.067 | 0.005 | 1 | P23588 | 1165.607 | 18.61 | 3 | 0.967 | 6.986 |
| LPLSLLK | 0.071 | 0.005 | 1 | Q9Y4Z0 | 783.534 | 29.12 | 2 | 1.959 | 31.485 |
| EFIQEPAK | 0.057 | 0.005 | 1 | O14744 | 961.499 | 48.57 | 2 | 1.233 | 20.747 |
| VQVEYKGETK | 0.078 | 0.008 | 1 | P11142 | 1180.621 | 29.63 | 3 | -1.634 | 14.385 |
| AVTKYTSSK | 0.078 | 0.008 | 1 | Q96A08 | 984.536 | 31.26 | 2 | 1.386 | 6.000 |
| EKEPIAASTNR | 0.078 | 0.008 | 1 | Q13813 | 1215.633 | 31.95 | 2 | 2.065 | 11.574 |
| LASYLDR | 0.075 | 0.008 | 1 | P05783 | 837.446 | 47.18 | 2 | 2.363 | 21.655 |
| VYEGERPLTK | 0.078 | 0.008 | 1 | P11021 | 1191.637 | 22.89 | 2 | 4.570 | 16.292 |
| LTPEEIER | 0.078 | 0.008 | 2 | P11021 | 986.515 | 19 | 2 | 2.294 | 21.657 |
| DLEGLSQR | 0.075 | 0.008 | 1 | P35579 | 917.469 | 35.69 | 2 | -0.610 | 22.570 |
| VGVNGFGR | 0.074 | 0.008 | 1 | P04406 | 805.432 | 37.59 | 2 | 3.022 | 21.030 |
| QLDQLSR | 0.075 | 0.008 | 1 | Q6P3W7 | 859.463 | 31.96 | 2 | -1.306 | 18.004 |
