# Supplementary material for: KPNA2 promotes renal cell carcinoma proliferation and metastasis via NPM
Source: J Cell Mol Med. 2021 Sep 1;25(19):9255–67. doi: 10.1111/jcmm.16846 (PMC8500977; doi:10.1111/jcmm.16846)
Supplement: Supplementary file 7 — Table S2 [file JCMM-25-9255-s004.docx]

| **Supplementray table2 peptide idetified in ACHN cells infected by KPNA2-overexpressed (OE)** | | | | | | | | | |
| --- | --- | --- | --- | --- | --- | --- | --- | --- | --- |
| Sequence | Qvality PEP | Qvality q-value | # PSMs | Master Protein Accessions | Theo. MH+ [Da] | Ions Score Mascot | Charge Mascot | DeltaM [ppm] Mascot | RT [min] Mascot |
| AEELVLER | 0.013 | 0 | 1 | P21980 | 1000.531 | 28.18 | 2 | 2.91 | 37.64 |
| QLSSGVSEIR | 0.003 | 0 | 1 | P04792 | 1075.574 | 40.14 | 2 | 5.16 | 21.16 |
| VGDYGSLSGR | 0.018 | 0 | 1 | Q9UBR2 | 1010.490 | 38.47 | 2 | 3.76 | 21.21 |
| LKGDDLQAIKK | 0.003 | 0 | 1 | P07910 | 1228.726 | 44.31 | 3 | 1.61 | 16.58 |
| SVTEQGAELSNEER | 0.003 | 0 | 1 | P63104 | 1548.714 | 52.17 | 2 | 1.14 | 21.24 |
| DANGNSFATR | 0.005 | 0 | 1 | P62701 | 1052.476 | 41.96 | 2 | 0.88 | 16.56 |
| GALALEEKR | 0.008 | 0 | 1 | P35579 | 986.563 | 58.13 | 2 | 1.66 | 16.56 |
| AGQSAAGAAPGGGVDTR | 0.000 | 0 | 1 | P21333 | 1442.698 | 43.78 | 2 | 1.64 | 16.55 |
| ILDQSEAEK | 0.008 | 0 | 1 | Q14764 | 1032.521 | 22.18 | 2 | 0.25 | 16.52 |
| SKAEAESLYQSK | 0.007 | 0 | 2 | P04264 | 1340.669 | 45.01 | 2 | 1.00 | 16.51 |
| EIGQSVDEVEK | 0.011 | 0 | 1 | Q01082 | 1232.600 | 48.83 | 2 | 3.68 | 21.25 |
| IGSNYSVK | 0.013 | 0 | 1 | Q9Y6N5 | 867.457 | 32.24 | 2 | 2.35 | 16.45 |
| GSSSGGGYSSGSSSYGSGGR | 0.002 | 0 | 1 | P35908 | 1740.706 | 42.51 | 2 | 0.37 | 16.39 |
| NQGGYGGSSSSSSYGSGR | 0.000 | 0 | 1 | P09651 | 1694.700 | 87.45 | 2 | 1.02 | 16.60 |
| AVTEQGHELSNEER | 0.003 | 0 | 2 | P31946 | 1598.741 | 41.74 | 3 | 2.07 | 16.38 |
| VAVVNQIAR | 0.016 | 0 | 1 | Q01082 | 969.584 | 60.3 | 2 | 2.75 | 21.30 |
| VYEGERPLTK | 0.018 | 0 | 2 | P11021 | 1191.637 | 28.03 | 2 | 1.09 | 16.28 |
| NSAPASVSPDGTR | 0.011 | 0 | 1 | Q8N1I0 | 1258.602 | 33.99 | 2 | -2.81 | 16.28 |
| NKYEDEINKR | 0.000 | 0 | 1 | P04264; P04259; P02538; P05787; P13647 | 1308.654 | 52.97 | 3 | 1.71 | 16.25 |
| TLCDYSK | 0.019 | 0 | 1 | O14744 | 886.397 | 24.41 | 2 | 0.02 | 16.24 |
| SAEIDSDDTGGSAAQK | 0.004 | 0 | 1 | P33176 | 1551.677 | 45.3 | 2 | -1.56 | 16.22 |
| NFQEEQINTR | 0.014 | 0 | 1 | Q8IXB1 | 1278.607 | 35.92 | 2 | 2.42 | 21.30 |
| KLNVTEQEKIDK | 0.001 | 0 | 1 | P06733 | 1444.801 | 29.28 | 3 | 1.11 | 16.20 |
| ELELQKEQR | 0.008 | 0 | 2 | Q13813 | 1172.627 | 44.01 | 2 | -2.17 | 16.18 |
| LEEAEKAADESER | 0.000 | 0 | 1 | P06753 | 1476.681 | 59.73 | 3 | 0.90 | 16.15 |
| IYIDSNNNPER | 0.004 | 0 | 1 | Q00610 | 1334.634 | 43.53 | 2 | 2.80 | 21.32 |
| GGSGGSYGGGGSGGGYGGGSGSR | 0.001 | 0 | 1 | P35527 | 1791.728 | 74.05 | 2 | -0.13 | 16.09 |
| EYQELMNVK | 0.015 | 0 | 1 | P04259; P02538; P05787 | 1169.551 | 21.1 | 2 | 1.99 | 21.28 |
| DLVQGEHLGR | 0.008 | 0 | 1 | P23458 | 1123.585 | 53.05 | 2 | 0.91 | 21.33 |
| AAAGGGGGGAAAAGR | 0.000 | 0 | 1 | Q9UL25 | 1113.540 | 104.96 | 2 | 0.58 | 16.60 |
| VNGSLAVSR | 0.007 | 0 | 3 | O75688 | 902.505 | 60.34 | 2 | 1.16 | 16.68 |
| VNFAMNVGK | 0.009 | 0 | 1 | P14618 | 995.498 | 36.27 | 2 | 1.63 | 20.73 |
| NSNPALNDNLEK | 0.009 | 0 | 1 | O00299 | 1328.644 | 30.28 | 2 | 1.29 | 20.73 |
| GTVVTGTLER | 0.009 | 0 | 1 | P49411 | 1032.568 | 32.56 | 2 | 3.67 | 20.77 |
| VTDALNATR | 0.018 | 0 | 1 | P10809 | 960.511 | 56.63 | 2 | -1.31 | 17.19 |
| ENAGEDPGLAR | 0.003 | 0 | 1 | P81605 | 1128.528 | 34.03 | 2 | 0.01 | 17.16 |
| EAGEQGDIEPR | 0.001 | 0 | 1 | Q13283 | 1200.549 | 43.44 | 2 | 1.22 | 17.15 |
| LAQFEPSQR | 0.004 | 0 | 1 | Q08211 | 1075.553 | 40.75 | 2 | 1.08 | 20.81 |
| EAALSTALSEKR | 0.010 | 0 | 1 | P02545 | 1275.690 | 36.32 | 2 | 1.47 | 20.89 |
| TQDQISNIK | 0.006 | 0 | 1 | Q14847 | 1046.548 | 19.23 | 2 | -0.13 | 17.03 |
| FSTPEQAAK | 0.021 | 0 | 1 | P14866 | 978.489 | 31.64 | 2 | 0.16 | 17.03 |
| SGGGGGGGGCGGGGGVSSLR | 0.002 | 0 | 1 | P13645 | 1549.677 | 56.37 | 2 | 2.98 | 17.02 |
| LKQQSELQSQVR | 0.001 | 0 | 1 | Q14847 | 1443.791 | 52.31 | 2 | 4.49 | 17.02 |
| DGLGSDNIGSR | 0.014 | 0 | 1 | P98175 | 1090.512 | 25.09 | 2 | 2.94 | 21.15 |
| SKSESPKEPEQLR | 0.008 | 0 | 1 | P09651 | 1556.791 | 16.3 | 3 | 1.49 | 17.01 |
| YIDQEELNK | 0.002 | 0 | 1 | P07900; P08238 | 1151.558 | 26.46 | 2 | 1.28 | 20.94 |
| VEQATKPSFESGR | 0.003 | 0 | 1 | P38159 | 1435.718 | 18.09 | 3 | 0.45 | 16.86 |
| GGSGGGGSISGGGYGSGGGSGGR | 0.009 | 0 | 1 | P35908 | 1741.748 | 58.1 | 2 | 0.22 | 16.85 |
| IVQAEGEAEAAK | 0.001 | 0 | 1 | Q99623 | 1215.622 | 59.33 | 2 | 1.77 | 16.84 |
| EVDEQMLNVQNK | 0.003 | 0 | 1 | Q9BVA1 | 1462.684 | 16.55 | 2 | 1.43 | 20.99 |
| IGQGYLIKDGK | 0.013 | 0 | 1 | P39023 | 1191.673 | 30.63 | 2 | 1.69 | 21.02 |
| ALSAVSAQAAAAQK | 0.002 | 0 | 1 | O00268 | 1286.706 | 52.1 | 2 | 2.23 | 21.06 |
| ALQASALK | 0.021 | 0 | 1 | P04075 | 801.483 | 43.05 | 2 | 1.39 | 16.73 |
| ITITNDQNR | 0.007 | 0 | 1 | P11021 | 1074.554 | 31.1 | 2 | 1.43 | 16.72 |
| QQSELQSQVR | 0.006 | 0 | 1 | Q14847 | 1202.612 | 34.33 | 2 | 2.51 | 16.71 |
| SSQSSSQQFSGIGR | 0.002 | 0 | 1 | Q92841 | 1455.682 | 52.07 | 2 | 1.12 | 21.09 |
| STTTGHLIYK | 0.003 | 0 | 2 | P68104 | 1120.600 | 36.99 | 3 | 0.88 | 16.72 |
| VAPAQPSEEGPGR | 0.004 | 0 | 1 | P23588 | 1294.639 | 27.46 | 2 | 0.47 | 17.00 |
| AGELTEDEVER | 0.002 | 0 | 1 | P62269 | 1247.575 | 42.14 | 2 | -0.78 | 21.33 |
| FSSSSGYGGGSSR | 0.000 | 0 | 2 | P35527 | 1235.529 | 53.73 | 2 | 1.59 | 16.04 |
| VAQGVSGAVQDK | 0.001 | 0 | 1 | P12268 | 1158.611 | 47.14 | 2 | 0.78 | 16.01 |
| VDVADQAQDKDRDDR | 0.001 | 0 | 2 | P23588 | 1745.805 | 18.99 | 3 | 2.56 | 15.21 |
| ATCAPQHGAPGPGPADASK | 0.001 | 0 | 1 | P21333 | 1789.829 | 35.28 | 3 | 4.22 | 15.10 |
| VDREQLVQK | 0.004 | 0 | 1 | P61981 | 1156.632 | 27.35 | 2 | 1.32 | 22.12 |
| VPVHDVTDASK | 0.003 | 0 | 1 | P21333 | 1167.600 | 24.24 | 3 | 1.29 | 15.07 |
| SCCSCCPVGCAK | 0.000 | 0 | 1 | P13640 | 1445.510 | 46.64 | 2 | 2.42 | 15.05 |
| SYVTTSTR | 0.008 | 0 | 1 | P08670 | 914.458 | 45.92 | 2 | 0.56 | 15.04 |
| VGEFSGANK | 0.014 | 0 | 1 | P10599 | 908.447 | 35.58 | 2 | 0.71 | 15.03 |
| LEAALGEAKK | 0.002 | 0 | 1 | P02545 | 1029.594 | 21.41 | 2 | 0.39 | 14.99 |
| LEGGSGGDSEVQR | 0.000 | 0 | 1 | P62195 | 1290.592 | 69.59 | 2 | -1.20 | 14.96 |
| EITALAPSTMK | 0.001 | 0 | 1 | P60709 | 1177.613 | 26.46 | 2 | -2.05 | 22.20 |
| VLENAEGAR | 0.014 | 0 | 1 | P38646 | 958.495 | 57.9 | 2 | 0.75 | 14.87 |
| IECDDKGDGSCDVR | 0.011 | 0 | 1 | P21333; Q14315 | 1625.653 | 33.16 | 3 | 0.66 | 14.83 |
| DSYVGDEAQSKR | 0.000 | 0 | 3 | P60709 | 1354.623 | 64.07 | 2 | 0.89 | 15.77 |
| VMEEEGLKDEEKR | 0.001 | 0 | 1 | Q15208 | 1607.758 | 37.96 | 3 | -0.25 | 14.79 |
| GTGIVSAPVPK | 0.009 | 0 | 1 | P15880 | 1025.599 | 30.95 | 2 | -1.24 | 22.38 |
| GDREQLLQR | 0.003 | 0 | 1 | Q04917 | 1156.607 | 23.47 | 2 | 0.48 | 22.39 |
| EQVANSAFVER | 0.002 | 0 | 1 | P08238 | 1249.617 | 59.25 | 2 | -1.68 | 22.40 |
| LGIHEDSTNR | 0.013 | 0 | 1 | P08238 | 1141.560 | 42.99 | 2 | 1.17 | 14.71 |
| LASYLDKVR | 0.005 | 0 | 2 | P08727; P13645; Q04695; P08779 | 1064.610 | 29.9 | 3 | -2.41 | 22.56 |
| DSQDAGGFGPEDR | 0.006 | 0 | 1 | Q15149 | 1350.556 | 47.71 | 2 | -1.68 | 22.57 |
| EVSTYIKK | 0.012 | 0 | 1 | P68104 | 967.546 | 35.45 | 2 | 0.08 | 14.68 |
| QQYESVAAK | 0.007 | 0 | 1 | P08670 | 1023.511 | 47.6 | 2 | 0.71 | 14.66 |
| GGSISGGGYGSGGGK | 0.001 | 0 | 1 | P35908 | 1197.549 | 41.87 | 2 | -0.48 | 14.63 |
| GAEAANVTGPGGVPVQGSK | 0.011 | 0 | 1 | P67809 | 1695.866 | 33.17 | 2 | -0.30 | 22.60 |
| TKQDEVNAAWQR | 0.002 | 0 | 1 | Q13813 | 1445.713 | 18.4 | 2 | 1.36 | 19.71 |
| LNPHRESDGASDEAEESGSQGK | 0.000 | 0 | 3 | O75688 | 2300.002 | 42.61 | 3 | 2.90 | 15.08 |
| EDSQRPGAHLTVK | 0.002 | 0 | 1 | P09651 | 1437.744 | 16.93 | 3 | 0.65 | 14.77 |
| STSSFSCLSR | 0.009 | 0 | 1 | P35908 | 1131.510 | 39.13 | 2 | 3.16 | 21.93 |
| SLVGLGGTK | 0.019 | 0 | 1 | P35908 | 831.493 | 44.4 | 2 | 3.10 | 21.78 |
| DAALATALGDKK | 0.004 | 0 | 1 | P20700 | 1173.647 | 60.27 | 2 | 1.67 | 21.75 |
| LPREPSNPER | 0.017 | 0 | 1 | P23588 | 1194.623 | 28.29 | 3 | 1.82 | 16.00 |
| VTVVDVNESR | 0.018 | 0 | 1 | O60701 | 1117.585 | 45.65 | 2 | 1.08 | 21.35 |
| MFGGPGTASRPSSSR | 0.001 | 0 | 1 | P08670 | 1510.707 | 43.92 | 3 | 0.97 | 15.96 |
| SDLYSSGR | 0.020 | 0 | 1 | P38159 | 884.411 | 22.82 | 2 | 0.80 | 15.87 |
| GHLENNPALEK | 0.013 | 0 | 1 | P05388 | 1221.622 | 33.06 | 2 | 2.11 | 15.86 |
| EVFTSSSSSSSR | 0.002 | 0 | 1 | P08779 | 1260.570 | 23.59 | 2 | -0.49 | 15.84 |
| ASITALEAK | 0.007 | 0 | 1 | P35579 | 903.515 | 58.19 | 2 | 1.62 | 21.43 |
| SESPKEPEQLR | 0.004 | 0 | 1 | P09651 | 1299.654 | 23.23 | 3 | 0.72 | 15.87 |
| SHEGETAYIR | 0.021 | 0 | 1 | Q07955 | 1162.549 | 35.44 | 3 | 1.46 | 15.80 |
| SKEQAELEAAR | 0.013 | 0 | 2 | Q15149 | 1231.628 | 39.03 | 2 | 3.29 | 15.75 |
| VSDEAVKKDSELDK | 0.005 | 0 | 1 | O75688 | 1562.791 | 32.59 | 3 | 1.80 | 15.75 |
| AEFTVETR | 0.009 | 0 | 1 | P21333 | 952.473 | 57.76 | 2 | 1.28 | 21.43 |
| KLLEGEEER | 0.016 | 0 | 1 | P20700; P02545; Q03252 | 1102.574 | 28.85 | 2 | 0.68 | 15.73 |
| QLIVGVNK | 0.020 | 0 | 2 | P68104 | 870.541 | 41.92 | 2 | -2.07 | 21.49 |
| VEAKPEVQSQPPR | 0.003 | 0 | 1 | Q9UN86 | 1464.781 | 42.13 | 3 | 0.02 | 15.67 |
| ESEAVEWQQK | 0.015 | 0 | 1 | P26038 | 1233.575 | 21.35 | 2 | -1.66 | 21.56 |
| ALVADSHPESER | 0.005 | 0 | 2 | Q01082 | 1310.634 | 54.85 | 2 | 0.80 | 15.69 |
| VHIEIGPDGR | 0.015 | 0 | 1 | P31943 | 1092.580 | 41.21 | 2 | 0.79 | 21.58 |
| QTVAVGVIK | 0.021 | 0 | 1 | P68104 | 914.567 | 34.69 | 2 | 2.40 | 21.59 |
| AQQELEEQTR | 0.019 | 0 | 1 | P26038 | 1231.591 | 18.81 | 2 | -0.57 | 15.58 |
| LQAAYAGDK | 0.013 | 0 | 1 | Q01082 | 936.479 | 39.06 | 2 | 2.19 | 15.57 |
| TTLTAAITK | 0.012 | 0 | 1 | P49411 | 919.546 | 45.42 | 2 | 1.41 | 21.64 |
| VRTELADKVTK | 0.002 | 0 | 1 | P35579 | 1259.732 | 32.89 | 3 | 2.36 | 15.57 |
| SQYEQLAEQNR | 0.000 | 0 | 1 | P13645 | 1365.639 | 43.31 | 2 | 1.34 | 21.66 |
| TNAENEFVTIKK | 0.000 | 0 | 2 | P04264 | 1393.732 | 62.34 | 2 | 1.53 | 21.71 |
| GELAIKDANAK | 0.001 | 0 | 1 | P05787 | 1129.621 | 66.29 | 2 | 0.95 | 15.45 |
| QQPDTEAVLNGK | 0.000 | 0 | 1 | P54105 | 1299.654 | 56.23 | 2 | 4.27 | 21.74 |
| DQNTVETLQR | 0.004 | 0 | 1 | Q01082 | 1203.596 | 52.7 | 2 | 0.67 | 20.66 |
| LENEIQTYR | 0.002 | 0 | 1 | P13645 | 1165.585 | 40.54 | 2 | 4.07 | 22.62 |
| LQAEEVAQQK | 0.010 | 0 | 1 | Q15149 | 1143.600 | 24.18 | 2 | 1.35 | 17.34 |
| IVSGKDYNVTANSK | 0.000 | 0 | 1 | P00338 | 1495.775 | 36.14 | 3 | 0.24 | 17.37 |
| TSVDFKDTDYKR | 0.007 | 0 | 1 | P49902 | 1474.717 | 19.34 | 3 | 3.09 | 19.21 |
| IAVAAQNCYK | 0.002 | 0 | 1 | P60174 | 1137.572 | 39.68 | 2 | 1.58 | 19.20 |
| ITESEEVVSR | 0.003 | 0 | 1 | P02545 | 1148.579 | 68.91 | 2 | 1.84 | 19.19 |
| AEAESLYQSK | 0.004 | 0 | 1 | P04264 | 1125.542 | 44.79 | 2 | 2.53 | 19.18 |
| YLSEVASGDNK | 0.001 | 0 | 1 | P31946 | 1182.564 | 30.49 | 2 | 2.01 | 19.16 |
| LKDDEVAQLK | 0.014 | 0 | 1 | P07195 | 1158.636 | 22.42 | 2 | 3.30 | 19.15 |
| YKEVAELTR | 0.012 | 0 | 1 | Q01082 | 1108.600 | 40.32 | 2 | 2.27 | 19.15 |
| AENQSTTLPGPGR | 0.010 | 0 | 1 | Q7Z353 | 1327.660 | 38.77 | 2 | 1.57 | 19.13 |
| AQYEDIAQK | 0.013 | 0 | 1 | P04264 | 1065.521 | 20.85 | 2 | 0.96 | 19.13 |
| TNRPPLSLSR | 0.002 | 0 | 1 | Q07020 | 1140.648 | 31.15 | 3 | 1.74 | 19.17 |
| VGFAEAAR | 0.022 | 0 | 1 | P07339 | 820.431 | 40.6 | 2 | 1.66 | 19.12 |
| ATTATMATSGSAR | 0.001 | 0 | 1 | P38919 | 1283.590 | 57.79 | 2 | -3.58 | 19.11 |
| SGTSEFLNK | 0.006 | 0 | 1 | P14625 | 982.484 | 52.35 | 2 | 0.86 | 20.09 |
| VYELQASR | 0.019 | 0 | 1 | Q9Y262 | 965.505 | 27.22 | 2 | 2.19 | 20.11 |
| VEIIANDQGNR | 0.008 | 0 | 4 | P11142; P11021; P0DMV9 | 1228.628 | 47.16 | 2 | 4.52 | 20.14 |
| YEELQQTAGR | 0.002 | 0 | 1 | P13647 | 1194.575 | 30.33 | 2 | -1.38 | 19.03 |
| SGVSLAALKK | 0.008 | 0 | 1 | P10412 | 973.604 | 29.85 | 2 | 1.12 | 20.15 |
| QGANINEIR | 0.007 | 0 | 1 | Q15365 | 1014.533 | 60.33 | 2 | 3.21 | 18.99 |
| HGGGGGGFGGGGFGSR | 0.000 | 0 | 2 | P35908 | 1320.583 | 37.03 | 2 | 1.93 | 18.98 |
| AAFTECCQAADK | 0.006 | 0 | 1 | P02768 | 1371.567 | 11.2 | 2 | -0.31 | 18.95 |
| LLVSASQDGK | 0.016 | 0 | 1 | P16520 | 1017.558 | 22.67 | 2 | 3.76 | 18.93 |
| QLEEAEEEAQR | 0.000 | 0 | 1 | P35579 | 1331.607 | 51.12 | 2 | -0.93 | 18.92 |
| LVSDGNINSDR | 0.001 | 0 | 1 | Q01082 | 1189.581 | 44.54 | 2 | 1.01 | 18.91 |
| ELEAENYHDIKR | 0.011 | 0 | 1 | Q01082 | 1516.739 | 12.47 | 3 | 0.58 | 18.82 |
| TLLEGEESR | 0.004 | 0 | 2 | P04264 | 1033.516 | 40.65 | 2 | 2.37 | 20.18 |
| VTNIGNQQIDK | 0.000 | 0 | 1 | Q6P3W7 | 1229.648 | 58.79 | 2 | 0.33 | 18.76 |
| AEAESMYQIK | 0.020 | 0 | 1 | P05787 | 1185.546 | 7.76 | 2 | 2.54 | 19.10 |
| LAEFQTDSQGK | 0.002 | 0 | 1 | Q86TI2 | 1223.590 | 37.92 | 2 | 0.30 | 20.24 |
| YICENQDSISSK | 0.016 | 0 | 2 | P02768 | 1443.642 | 20.37 | 2 | 2.02 | 20.09 |
| QLDEPKLER | 0.004 | 0 | 3 | P23588 | 1127.606 | 51.17 | 2 | 2.28 | 19.27 |
| NDNDTFTVK | 0.005 | 0 | 1 | P21333; Q14315 | 1053.485 | 27.27 | 2 | 1.56 | 19.70 |
| SYSSGGEDGYVR | 0.005 | 0 | 1 | Q13347 | 1276.544 | 39.7 | 2 | -0.89 | 19.75 |
| HTGPNSPDTANDGFVR | 0.000 | 0 | 2 | P31943 | 1684.767 | 89.14 | 2 | -0.65 | 19.84 |
| SLLEGEGSSGGGGR | 0.002 | 0 | 2 | P13645 | 1262.597 | 58.22 | 2 | 0.16 | 19.83 |
| VAIESAVQQK | 0.003 | 0 | 1 | Q68CP9 | 1072.600 | 29.8 | 2 | 0.53 | 19.86 |
| SNVSDAVAQSTR | 0.001 | 0 | 1 | P60174 | 1234.602 | 70.85 | 2 | 3.36 | 19.86 |
| LITEDVQGK | 0.008 | 0 | 1 | P61247 | 1002.547 | 19.89 | 2 | 1.17 | 19.87 |
| ATAVMPDGQFK | 0.014 | 0 | 1 | Q06830 | 1180.567 | 21.52 | 2 | -0.30 | 19.88 |
| EVATNSELVQSGK | 0.000 | 0 | 1 | Q04695 | 1361.691 | 28.43 | 2 | -1.15 | 19.55 |
| SGEEGMPDLAHVMR | 0.004 | 0 | 1 | O75688 | 1560.678 | 9.34 | 3 | 1.47 | 20.07 |
| AAYEAELGDARK | 0.001 | 0 | 2 | P02545 | 1293.643 | 41.66 | 2 | -0.75 | 19.52 |
| QAASSLQQASLK | 0.000 | 0 | 1 | P38646 | 1231.664 | 78 | 2 | 4.28 | 19.46 |
| SGAVEETFR | 0.013 | 0 | 1 | Q6YN16 | 995.479 | 24.78 | 2 | 0.11 | 20.08 |
| VGQAVDVVGQAGKPK | 0.003 | 0 | 1 | Q13200 | 1452.817 | 0.94 | 2 | -1.40 | 19.44 |
| AQYEEIAQR | 0.016 | 0 | 1 | P35908; P04259; P02538 | 1107.543 | 15.89 | 2 | -0.46 | 20.47 |
| GGDLMAYDR | 0.020 | 0 | 1 | P61978 | 1013.436 | 17.12 | 2 | 0.71 | 19.43 |
| VQAVVAVAR | 0.009 | 0 | 1 | Q01082 | 912.563 | 29.61 | 2 | 0.16 | 19.42 |
| AAMAVGGAGGSR | 0.000 | 0 | 3 | O14744 | 1062.500 | 55.75 | 2 | 1.39 | 19.92 |
| QGGGGGGGSVPGIER | 0.016 | 0 | 1 | P52272 | 1284.629 | 51.43 | 2 | 1.02 | 19.42 |
| GTQGAEEVLR | 0.014 | 0 | 1 | Q15149 | 1059.543 | 37.29 | 2 | -1.98 | 19.41 |
| ALANSLACQGK | 0.005 | 0 | 1 | P04075 | 1132.578 | 30.62 | 2 | 1.09 | 19.36 |
| QVSDDLTER | 0.004 | 0 | 1 | P35232 | 1062.506 | 57.18 | 2 | -0.84 | 19.35 |
| ALAAAGYDVEKNNSR | 0.001 | 0 | 1 | P10412 | 1578.787 | 20.98 | 3 | 0.52 | 20.02 |
| TAVCDIPPR | 0.006 | 0 | 1 | Q9BVA1 | 1028.519 | 40.73 | 2 | 2.72 | 19.30 |
| AVVGVVAGGGR | 0.012 | 0 | 1 | P62917 | 941.553 | 36.15 | 2 | 1.72 | 19.30 |
| DNTNEIYSGK | 0.007 | 0 | 1 | P05556 | 1140.517 | 21.86 | 2 | 2.79 | 19.29 |
| LCTSATESEVAR | 0.006 | 0 | 1 | P31930 | 1323.621 | 11.68 | 2 | -0.47 | 19.43 |
| EVYQQQQYGSGGR | 0.004 | 0 | 1 | Q99729 | 1499.687 | 46.35 | 2 | 4.20 | 18.70 |
| DWNTLIVGK | 0.013 | 0 | 1 | O14744 | 1045.568 | 35.97 | 2 | 1.29 | 37.29 |
| QLETLGQEK | 0.003 | 0 | 1 | P05787 | 1045.552 | 44.1 | 2 | 2.80 | 18.66 |
| EQEVNILKK | 0.012 | 0 | 1 | P35579 | 1100.631 | 25.52 | 2 | -0.42 | 18.02 |
| AAIISAEGDSK | 0.002 | 0 | 1 | P35232 | 1061.547 | 31.09 | 2 | 0.31 | 17.98 |
| DYQELMNTK | 0.017 | 0 | 1 | P04264 | 1157.514 | 26.5 | 2 | -0.20 | 20.36 |
| SLETENAGLR | 0.010 | 0 | 1 | P02545 | 1089.553 | 52.83 | 2 | 3.73 | 20.37 |
| LNVEAVNTHR | 0.003 | 0 | 2 | O60825 | 1152.612 | 41.45 | 2 | 1.47 | 17.92 |
| TPGPGAQSALR | 0.019 | 0 | 1 | P62263 | 1054.564 | 45.3 | 2 | 0.84 | 17.92 |
| LLADQAEAR | 0.003 | 0 | 1 | P84098 | 986.527 | 59.91 | 2 | -0.31 | 18.32 |
| YADQEVPR | 0.013 | 0 | 1 | Q14315 | 977.469 | 23.83 | 2 | -0.95 | 17.80 |
| IQEAGTEVVK | 0.003 | 0 | 1 | P40926 | 1073.584 | 16.32 | 2 | 0.41 | 17.80 |
| VLATVTKPVGGDK | 0.004 | 0 | 1 | Q02878 | 1284.752 | 41.09 | 2 | 1.38 | 17.80 |
| INISEGNCPER | 0.004 | 0 | 1 | Q15365 | 1288.595 | 56.97 | 2 | 3.40 | 20.39 |
| LLEAAAQSTK | 0.003 | 0 | 1 | Q15149 | 1031.573 | 45.74 | 2 | 1.08 | 17.75 |
| VLANPGNSQVAR | 0.014 | 0 | 1 | Q14974 | 1225.665 | 16.5 | 2 | 0.27 | 18.02 |
| AATALKDVVK | 0.004 | 0 | 1 | Q15084 | 1015.615 | 39.92 | 2 | 0.65 | 17.70 |
| QDVDNASLAR | 0.001 | 0 | 3 | P08670 | 1088.533 | 79.15 | 2 | 1.62 | 17.66 |
| GSGTAEVELK | 0.014 | 0 | 1 | P14618 | 990.510 | 34.68 | 2 | 1.01 | 17.63 |
| TNQELQEINR | 0.003 | 0 | 1 | P07355 | 1244.623 | 27.71 | 2 | 2.27 | 20.47 |
| LAGESESNLR | 0.001 | 0 | 1 | P55072 | 1075.538 | 24.96 | 2 | 0.06 | 17.61 |
| DSELDKHLESR | 0.001 | 0 | 2 | O75688 | 1328.644 | 73.12 | 2 | 0.28 | 17.75 |
| SDEGQLSPATR | 0.001 | 0 | 1 | Q15149 | 1160.554 | 55.53 | 2 | -1.02 | 17.57 |
| GPSSVEDIK | 0.012 | 0 | 1 | P06748 | 931.473 | 33.83 | 2 | 0.88 | 17.51 |
| GFSGLDGAK | 0.016 | 0 | 1 | P02452 | 851.426 | 30 | 2 | 1.30 | 20.48 |
| DSYVGDEAQSK | 0.002 | 0 | 2 | P60709 | 1198.522 | 33.28 | 2 | 1.45 | 17.47 |
| AVTEQGAELSNEER | 0.003 | 0 | 1 | P27348 | 1532.719 | 64.09 | 2 | 0.78 | 20.49 |
| AATASAGAGGIDGKPR | 0.001 | 0 | 1 | Q02978 | 1441.739 | 58.63 | 2 | 0.54 | 20.62 |
| AFEEDQVAGR | 0.006 | 0 | 1 | O43818 | 1121.522 | 23.35 | 2 | 2.58 | 20.65 |
| GESPVDYDGGR | 0.013 | 0 | 1 | Q15084 | 1151.496 | 55.64 | 2 | 0.77 | 20.40 |
| VNEVNQFAAK | 0.003 | 0 | 1 | Q13813 | 1119.579 | 23.07 | 2 | -0.57 | 20.35 |
| VLNTNIDGR | 0.005 | 0 | 1 | P62269 | 1001.537 | 50.79 | 2 | 3.01 | 20.31 |
| SQYEVMAEQNR | 0.002 | 0 | 1 | P08727 | 1370.601 | 25.87 | 2 | -0.35 | 18.04 |
| TEADAEKTFEEK | 0.002 | 0 | 1 | P19338 | 1397.643 | 44.91 | 3 | 1.26 | 18.65 |
| NVLVESEHQVK | 0.018 | 0 | 1 | P23458 | 1281.680 | 19.75 | 2 | 1.52 | 18.65 |
| TYETTLEK | 0.009 | 0 | 1 | P02768 | 984.488 | 14.09 | 2 | 2.20 | 18.64 |
| FEMEQNLR | 0.020 | 0 | 1 | P35527 | 1082.494 | 19.62 | 2 | 0.62 | 18.64 |
| SQLLGSAHEVQR | 0.000 | 0 | 2 | Q13813 | 1324.697 | 59.71 | 2 | 2.24 | 18.63 |
| SAVTTVVNPK | 0.009 | 0 | 1 | P05556 | 1015.578 | 62.62 | 2 | 3.18 | 18.62 |
| IVADKDYSVTANSK | 0.000 | 0 | 2 | P07195 | 1510.775 | 77.43 | 2 | 3.03 | 18.62 |
| CCTESLVNR | 0.002 | 0 | 1 | P02768 | 1138.498 | 33.54 | 2 | 1.84 | 18.61 |
| LIQSHPESAEDLQEK | 0.002 | 0 | 1 | Q13813 | 1723.850 | 20.17 | 3 | 0.42 | 20.28 |
| LATNTSAPDLK | 0.011 | 0 | 1 | P27816 | 1130.605 | 18.46 | 2 | 1.16 | 20.26 |
| IADGYEQAAR | 0.003 | 0 | 1 | P48643 | 1093.527 | 26.22 | 2 | -0.55 | 18.53 |
| SLAAEEEAAR | 0.003 | 0 | 1 | Q15149 | 1046.511 | 42.85 | 2 | 1.75 | 18.49 |
| AENPSLENHR | 0.003 | 0 | 1 | O00505 | 1208.565 | 27.28 | 2 | 0.84 | 18.42 |
| NHEEEISTLR | 0.001 | 0 | 2 | P08727 | 1227.596 | 35.4 | 2 | -3.02 | 18.36 |
| VASLEESEGNKQDLK | 0.006 | 0 | 2 | Q07065 | 1646.823 | 28.32 | 2 | 0.15 | 18.38 |
| AQAEVEGLGK | 0.010 | 0 | 1 | Q15149 | 1001.526 | 37.96 | 2 | 0.88 | 18.35 |
| LLEQYKEESK | 0.002 | 0 | 1 | Q00839 | 1266.658 | 42.73 | 2 | -1.13 | 18.33 |
| NVESGEEELASK | 0.011 | 0 | 1 | Q96HS1 | 1291.601 | 42.07 | 2 | 2.76 | 20.26 |
| AGFAGDDAPR | 0.004 | 0 | 3 | P60709 | 976.448 | 69.98 | 2 | -0.37 | 18.32 |
| SGAQASSTPLSPTR | 0.012 | 0 | 1 | P02545 | 1359.686 | 53.19 | 2 | 2.28 | 18.24 |
| AQYEDIANR | 0.006 | 0 | 1 | P05787 | 1079.512 | 50.5 | 2 | 1.73 | 20.27 |
| SGASSSEQNNNSYETK | 0.000 | 0 | 1 | O60825 | 1744.726 | 46.64 | 2 | -1.68 | 18.23 |
| ETTFSSNCAGCGR | 0.000 | 0 | 1 | O14639 | 1446.574 | 33.1 | 2 | -1.00 | 18.17 |
| KVIDDTNITR | 0.003 | 0 | 1 | P05783 | 1174.643 | 37.38 | 2 | -1.35 | 18.16 |
| VSSPDYPER | 0.008 | 0 | 1 | O60825 | 1049.490 | 52.27 | 2 | -1.53 | 18.11 |
| ASREEILAQAK | 0.003 | 0 | 1 | P35579 | 1215.669 | 33.16 | 2 | -2.16 | 18.07 |
| SQYEQLAEQNRK | 0.000 | 0 | 1 | P13645 | 1493.734 | 48.84 | 2 | -1.14 | 18.05 |
| VIGSGCNLDSAR | 0.000 | 0 | 1 | P07195; P00338 | 1248.600 | 74.9 | 2 | 1.98 | 20.65 |
| SQLEEQAAR | 0.017 | 0 | 1 | Q6KB66 | 1031.512 | 21.43 | 2 | 1.69 | 14.52 |
| KLLEGEESR | 0.015 | 0 | 1 | P08670; P05787 | 1060.563 | 32.77 | 3 | 0.19 | 14.59 |
| VVTDTDETELAR | 0.001 | 0 | 1 | P05198 | 1348.659 | 35.54 | 2 | -1.33 | 22.63 |
| FDQLLAEEK | 0.013 | 0 | 1 | P35579 | 1092.557 | 41.46 | 2 | -1.55 | 26.52 |
| LLPDDPYEK | 0.013 | 0 | 1 | P78417 | 1089.546 | 32.54 | 2 | 0.49 | 26.59 |
| AKEAAEQDVEK | 0.001 | 0 | 1 | P26373 | 1217.601 | 44.99 | 2 | 0.95 | 7.21 |
| TGSESSQTGTSTTSSR | 0.000 | 0 | 5 | P23588 | 1573.694 | 98.37 | 2 | -1.22 | 7.19 |
| NRPAPYSRPK | 0.010 | 0 | 1 | Q86V81 | 1185.649 | 8.06 | 3 | 0.08 | 7.18 |
| TAAAVAAQSGILDR | 0.007 | 0 | 1 | Q9Y6N5 | 1343.728 | 39.76 | 2 | 1.24 | 26.70 |
| ANTFVAELK | 0.017 | 0 | 1 | P40926 | 992.541 | 20.26 | 2 | -0.92 | 26.71 |
| YEELQITAGR | 0.001 | 0 | 1 | P04264; P04259 | 1179.600 | 44.47 | 2 | -0.65 | 26.78 |
| QVAQQEAQR | 0.003 | 0 | 1 | Q99623 | 1057.538 | 55.21 | 2 | 3.93 | 7.11 |
| ALIAAQYSGAQVR | 0.021 | 0 | 1 | P26641 | 1347.738 | 38.94 | 2 | -0.15 | 26.80 |
| GSGGGSSGGSIGGR | 0.000 | 0 | 2 | P04264 | 1092.503 | 75.83 | 2 | -3.78 | 7.06 |
| DDRGPPQRPK | 0.004 | 0 | 1 | P23588 | 1165.607 | 8.92 | 3 | -2.33 | 7.06 |
| GAHPSGGADDVAK | 0.007 | 0 | 2 | Q07065 | 1181.555 | 34.78 | 2 | 0.68 | 7.04 |
| GGSDGYGSGR | 0.013 | 0 | 1 | P22626 | 912.381 | 24.24 | 2 | -1.54 | 7.04 |
| NVTELNEPLSNEER | 0.006 | 0 | 1 | P61981 | 1643.787 | 44.84 | 2 | 1.36 | 26.82 |
| NDKSEEEQSSSSVK | 0.000 | 0 | 1 | P07910 | 1553.693 | 79.14 | 2 | -2.12 | 6.98 |
| AQQDKLNTR | 0.008 | 0 | 1 | Q01082 | 1073.570 | 48.88 | 2 | -2.77 | 6.93 |
| NHEEEMKDLR | 0.016 | 0 | 1 | P13645 | 1316.590 | 30.79 | 2 | -1.15 | 6.92 |
| IGPLGLSPK | 0.010 | 0 | 1 | P30050 | 881.545 | 35.94 | 2 | 1.50 | 26.92 |
| RATVVESSEK | 0.010 | 0 | 1 | P61981 | 1105.585 | 23.39 | 2 | -1.78 | 6.90 |
| KGDSSAEELK | 0.014 | 0 | 1 | P26373 | 1063.527 | 35.94 | 2 | -0.40 | 6.88 |
| KDENKVDGMNAPK | 0.000 | 0 | 1 | P23588 | 1461.700 | 62.88 | 2 | -4.16 | 6.84 |
| SIRDTPAK | 0.009 | 0 | 1 | P06748 | 887.495 | 19.93 | 2 | 0.05 | 6.84 |
| IENHEGVR | 0.008 | 0 | 1 | P14618 | 953.480 | 57.44 | 2 | -0.91 | 6.81 |
| YLDGLTAER | 0.013 | 0 | 1 | P35908 | 1037.526 | 31.98 | 2 | 1.74 | 26.93 |
| FLEQQNQVLQTK | 0.001 | 0 | 1 | P35908; P04264 | 1475.785 | 60.3 | 2 | -1.95 | 26.51 |
| FNDILGR | 0.020 | 0 | 1 | P68400 | 834.447 | 41.45 | 2 | -0.74 | 26.50 |
| AAVAGEDGR | 0.005 | 0 | 1 | P07910 | 845.411 | 47.42 | 2 | -0.28 | 7.30 |
| HQGVMVGMGQK | 0.002 | 0 | 1 | P60709 | 1203.561 | 26.79 | 2 | 0.06 | 7.30 |
| SGYLLPDTK | 0.010 | 0 | 1 | P14625 | 993.525 | 24.9 | 2 | 1.35 | 25.76 |
| NKEEAAEYAK | 0.006 | 0 | 1 | P62753 | 1152.553 | 30.92 | 2 | 1.17 | 8.12 |
| LNECVDHTPK | 0.004 | 0 | 1 | P78417 | 1212.568 | 14.35 | 2 | 0.34 | 14.52 |
| IGGIGTVPVGR | 0.015 | 0 | 1 | P68104 | 1025.610 | 49.83 | 2 | 1.26 | 25.97 |
| STPKEDDSSASTSQSTR | 0.000 | 0 | 5 | P23588 | 1783.794 | 97.6 | 3 | 2.25 | 8.58 |
| AIETTDIISR | 0.004 | 0 | 1 | Q96HS1 | 1118.605 | 47.37 | 2 | 0.74 | 25.98 |
| EATWTMSNITAGR | 0.008 | 0 | 1 | P52292 | 1453.674 | 38.32 | 2 | 3.69 | 26.21 |
| YEELQSLAGK | 0.001 | 0 | 1 | P05787 | 1137.579 | 57.11 | 2 | 2.17 | 26.27 |
| EAGGGGVGGPGAK | 0.002 | 0 | 1 | Q8NC51 | 1013.501 | 39.71 | 2 | -0.32 | 7.90 |
| QGVDADINGLR | 0.000 | 0 | 1 | P35527 | 1157.591 | 71.02 | 2 | 0.37 | 26.35 |
| QVAQQEAER | 0.006 | 0 | 1 | P35232 | 1058.523 | 52.62 | 2 | 0.92 | 7.81 |
| VDQLKSDQSR | 0.008 | 0 | 1 | P04264 | 1175.601 | 26.6 | 3 | 1.13 | 7.86 |
| SRTGSESSQTGTSTTSSR | 0.000 | 0 | 3 | P23588 | 1816.827 | 107.88 | 2 | -1.17 | 6.81 |
| LQKEQEKLQR | 0.006 | 0 | 1 | P23588 | 1299.738 | 28.95 | 3 | 0.96 | 7.78 |
| KYEEVAR | 0.021 | 0 | 1 | P06753 | 894.468 | 23.11 | 2 | 0.80 | 7.74 |
| RGDDSFGDK | 0.009 | 0 | 1 | P23588 | 996.438 | 44.45 | 2 | 0.47 | 7.56 |
| IVLQIDNAR | 0.003 | 0 | 1 | P08727; P05783 | 1041.605 | 57.65 | 2 | 0.62 | 26.42 |
| SSEEIESAFR | 0.005 | 0 | 1 | Q13813 | 1154.532 | 34.53 | 2 | -0.01 | 26.43 |
| NDLAVVDVR | 0.003 | 0 | 1 | P19338 | 1000.542 | 57.48 | 2 | -0.88 | 26.45 |
| QIEAQEKPR | 0.007 | 0 | 1 | Q01082 | 1098.590 | 34.42 | 2 | -0.72 | 7.39 |
| QGVLTHGR | 0.013 | 0 | 1 | P62753 | 867.480 | 46.29 | 2 | 0.93 | 7.36 |
| EDDSSASTSQSTR | 0.000 | 0 | 1 | P23588 | 1370.567 | 65.25 | 2 | -1.10 | 7.36 |
| TNEGVIEFR | 0.008 | 0 | 1 | Q13247 | 1064.537 | 36.94 | 2 | -1.55 | 26.47 |
| SLYASSPGGVYATR | 0.008 | 0 | 1 | P08670 | 1428.712 | 34.23 | 2 | -0.81 | 26.49 |
| IVTDRETGSSK | 0.000 | 0 | 2 | P19338 | 1192.617 | 67.95 | 2 | 0.56 | 7.35 |
| AAAEAAAEAK | 0.011 | 0 | 1 | Q9UNF1 | 902.458 | 31.4 | 2 | 1.45 | 7.32 |
| QEMQEVQSSR | 0.001 | 0 | 1 | P22626 | 1237.548 | 54.96 | 2 | 1.31 | 7.74 |
| RDDRGPPQRPK | 0.007 | 0 | 1 | P23588 | 1321.708 | 7.15 | 3 | 1.31 | 6.73 |
| VKVEPSHDASK | 0.001 | 0 | 1 | P21333 | 1196.627 | 34.9 | 2 | 1.65 | 6.72 |
| NSVSQISVLSGGK | 0.020 | 0 | 1 | O15143 | 1275.690 | 52.93 | 2 | -2.93 | 26.99 |
| EVFEDAAEIR | 0.015 | 0 | 1 | P19338 | 1178.569 | 37.48 | 2 | -1.99 | 29.06 |
| YKAEDEKQR | 0.005 | 0 | 1 | P11142 | 1166.580 | 28.5 | 2 | 0.82 | 2.69 |
| ADEVAPAKK | 0.005 | 0 | 1 | P53396 | 928.510 | 28.2 | 2 | 0.58 | 2.58 |
| AGAHLQGGAKR | 0.003 | 0 | 1 | P04406 | 1065.591 | 15.02 | 3 | 0.96 | 2.68 |
| VGSAAQTR | 0.019 | 0 | 1 | P25705 | 789.421 | 34.56 | 2 | 0.58 | 2.54 |
| SSSSGSVGESSSK | 0.001 | 0 | 1 | P13645 | 1185.523 | 55.66 | 2 | 0.54 | 2.45 |
| KHEEEEAKAER | 0.002 | 0 | 1 | P25789 | 1355.655 | 32.09 | 3 | 0.15 | 2.40 |
| TGISDVFAK | 0.001 | 0 | 1 | P19338 | 937.499 | 58.08 | 2 | -0.30 | 29.33 |
| ALSSQHQAR | 0.006 | 0 | 1 | P11021 | 997.517 | 46.58 | 2 | 0.99 | 2.39 |
| AALEDTLAETEAR | 0.000 | 0 | 1 | P08727 | 1389.686 | 79.14 | 2 | -1.07 | 29.59 |
| SGSALELSVENVK | 0.000 | 0 | 2 | O75688 | 1332.701 | 52.06 | 2 | 3.08 | 30.36 |
| TQEKEQIK | 0.006 | 0 | 1 | P05787 | 1003.542 | 42.43 | 2 | 0.86 | 2.21 |
| TVDGPSGK | 0.016 | 0 | 2 | P04406 | 760.384 | 44.67 | 2 | 1.01 | 3.43 |
| TLSKEEETKK | 0.017 | 0 | 1 | P84098 | 1192.642 | 33.02 | 3 | 0.47 | 2.00 |
| VLDELTLTK | 0.004 | 0 | 1 | P13645 | 1031.598 | 51.64 | 2 | 4.27 | 30.01 |
| ASDVHEVRK | 0.020 | 0 | 1 | P14618 | 1040.548 | 21.79 | 2 | 0.05 | 1.76 |
| TDAAVSFAK | 0.002 | 0 | 1 | P05141 | 951.478 | 44.83 | 2 | 3.41 | 30.22 |
| VSALEVLPDR | 0.015 | 0 | 1 | Q9Y657 | 1098.615 | 46.5 | 2 | 2.94 | 30.36 |
| LSELEAALQR | 0.001 | 0 | 1 | P05787 | 1129.621 | 63.3 | 2 | 3.65 | 30.49 |
| ITALDEFATK | 0.001 | 0 | 1 | Q13813 | 1108.588 | 47.34 | 2 | 1.07 | 31.11 |
| KPEENPASK | 0.004 | 0 | 2 | P23588 | 999.511 | 64.39 | 2 | -0.37 | 1.47 |
| GDLGIEIPAEK | 0.001 | 0 | 1 | P14618 | 1141.610 | 48.09 | 2 | -3.44 | 31.24 |
| LLLPGELAK | 0.015 | 0 | 1 | O60814 | 953.603 | 35.76 | 2 | 5.78 | 31.75 |
| VTAEDKGTGNK | 0.016 | 0 | 1 | P11021 | 1119.564 | 47.82 | 2 | 0.74 | 0.83 |
| DALNIETAIK | 0.010 | 0 | 1 | P07355 | 1087.599 | 37.37 | 2 | 4.87 | 32.13 |
| TLPVVFDSPR | 0.019 | 0 | 1 | Q9ULW8 | 1130.620 | 33.22 | 2 | 4.19 | 32.13 |
| TTPSYVAFTDTER | 0.000 | 0 | 1 | P11142; P0DMV9 | 1487.701 | 51.29 | 2 | 1.45 | 29.81 |
| ADSERQNQEYQR | 0.014 | 0 | 1 | P08727 | 1523.683 | 16.69 | 3 | 2.32 | 8.16 |
| VSDEAVKK | 0.019 | 0 | 2 | O75688 | 875.483 | 29.18 | 2 | 0.83 | 3.03 |
| TLLDIDNTR | 0.007 | 0 | 1 | P35527 | 1060.563 | 52.03 | 2 | 2.38 | 28.72 |
| TNAENEFVTIK | 0.001 | 0 | 1 | P04264 | 1265.637 | 53.31 | 2 | 1.68 | 27.01 |
| EELYQNLTR | 0.005 | 0 | 1 | Q13813 | 1165.585 | 36.6 | 2 | 1.98 | 27.03 |
| ATNFLAHEK | 0.006 | 0 | 1 | P29692 | 1072.542 | 7.72 | 2 | 4.53 | 27.14 |
| VVNPLFEK | 0.021 | 0 | 1 | P62424 | 945.540 | 21.69 | 2 | 2.58 | 27.28 |
| AVFPSIVGRPR | 0.004 | 0 | 1 | P60709 | 1198.706 | 15.84 | 3 | 3.14 | 27.31 |
| FSSSGGGGGGGR | 0.012 | 0 | 1 | P35527 | 982.434 | 43.61 | 2 | 1.75 | 6.70 |
| FSGSGSGTDFTLK | 0.000 | 0 | 2 | A0A075B6S2 | 1303.616 | 92.55 | 2 | 3.58 | 27.52 |
| VVDALGNAIDGK | 0.003 | 0 | 1 | P25705 | 1171.632 | 60.27 | 2 | 2.01 | 27.64 |
| SGGGYGGDR | 0.002 | 0 | 1 | Q92804 | 825.349 | 58.37 | 2 | -1.64 | 5.31 |
| ISEQSDAK | 0.021 | 0 | 1 | P25705 | 877.426 | 26.45 | 2 | -0.09 | 4.86 |
| NTGIICTIGPASR | 0.001 | 0 | 1 | P14618 | 1359.705 | 61.67 | 2 | 1.60 | 27.76 |
| RQEENDKLR | 0.021 | 0 | 1 | Q13813 | 1187.613 | 26.56 | 3 | 0.99 | 4.48 |
| NLLSVAYK | 0.010 | 0 | 1 | P63104; P31946; Q04917; P61981; P27348; P62258 | 907.525 | 35.78 | 2 | 1.24 | 28.77 |
| TKGTSSFGK | 0.022 | 0 | 1 | P61927 | 912.479 | 46.52 | 2 | -0.02 | 4.46 |
| KISSPTGSK | 0.009 | 0 | 1 | Q99623 | 904.510 | 20.11 | 2 | -0.95 | 4.22 |
| TAAAAAEHSQR | 0.008 | 0 | 1 | O43242 | 1112.544 | 40.08 | 2 | -1.05 | 4.18 |
| LGESQTLQQFSR | 0.001 | 0 | 1 | Q13813 | 1393.707 | 63.88 | 2 | 3.98 | 28.01 |
| DVNAAIATIK | 0.004 | 0 | 1 | P68363 | 1015.578 | 48.22 | 2 | 1.56 | 28.27 |
| KLEDGPK | 0.003 | 0 | 2 | P68104 | 786.436 | 53.78 | 2 | 0.16 | 4.35 |
| HNAHGAGNGLR | 0.002 | 0 | 1 | O75688 | 1103.545 | 21.58 | 3 | 2.25 | 3.82 |
| AEAGDNLGALVR | 0.001 | 0 | 1 | P49411 | 1185.622 | 65.91 | 2 | 0.42 | 28.30 |
| STDEVDSKR | 0.009 | 0 | 1 | Q01082 | 1036.491 | 34.27 | 2 | 1.87 | 3.64 |
| ISGLIYEETR | 0.003 | 0 | 1 | P62805 | 1180.621 | 46.39 | 2 | 0.26 | 28.42 |
| LAKEQEQAQK | 0.013 | 0 | 1 | Q6P3W7 | 1172.627 | 21 | 3 | 0.01 | 3.53 |
| GGAGVGSMTK | 0.017 | 0 | 1 | P39019 | 880.419 | 24.63 | 2 | 0.98 | 3.39 |
| TDSSPNQAR | 0.007 | 0 | 1 | P26599 | 975.449 | 30.49 | 2 | 0.51 | 3.29 |
| VLEGNEQFINAAK | 0.006 | 0 | 1 | P35030 | 1432.743 | 53.13 | 2 | 5.47 | 27.93 |
| IEISELNR | 0.008 | 0 | 1 | P35908; P04264 | 973.531 | 53.81 | 2 | 1.83 | 25.73 |
| LGLDYEER | 0.021 | 0 | 1 | Q99623 | 994.484 | 26 | 2 | -0.37 | 25.95 |
| AENSQLTER | 0.009 | 0 | 1 | Q9P2E9 | 1047.507 | 36.77 | 2 | -0.71 | 12.98 |
| QSVVYGGK | 0.018 | 0 | 1 | Q8WWY3 | 837.446 | 42.45 | 2 | -0.99 | 13.70 |
| NSPGSQVASNPR | 0.013 | 0 | 1 | Q9H0D6 | 1213.592 | 55.9 | 2 | 4.71 | 13.65 |
| ESDGASDEAEESGSQGK | 0.000 | 0 | 1 | O75688 | 1682.662 | 74.1 | 2 | 2.70 | 13.61 |
| QDEVNAAWQR | 0.002 | 0 | 1 | Q13813 | 1216.571 | 51.5 | 2 | 3.68 | 23.37 |
| VGGTSDVEVNEKKDR | 0.000 | 0 | 1 | P10809 | 1632.819 | 35.61 | 3 | 2.17 | 13.46 |
| SSVGPSKPVSQPR | 0.013 | 0 | 1 | Q9Y657 | 1325.717 | 17.17 | 3 | 0.67 | 13.44 |
| YSQYQQAIYK | 0.001 | 0 | 2 | O14744 | 1291.632 | 36.16 | 2 | 0.69 | 23.38 |
| GSGTAEVELKK | 0.008 | 0 | 1 | P14618 | 1118.605 | 57.14 | 2 | 1.72 | 13.42 |
| DVDNAYMIK | 0.003 | 0 | 1 | P35908 | 1084.498 | 14.14 | 2 | -0.31 | 23.42 |
| SESPKEPEQLRK | 0.003 | 0 | 1 | P09651 | 1427.749 | 29.73 | 3 | 2.02 | 13.21 |
| KYEDEINKR | 0.002 | 0 | 2 | P35908 | 1194.611 | 35.23 | 3 | 1.95 | 13.20 |
| VLETAEDIQER | 0.000 | 0 | 1 | Q13813 | 1302.654 | 55.94 | 2 | 0.49 | 23.45 |
| GTEEPVKVR | 0.021 | 0 | 1 | Q14315 | 1014.558 | 21.98 | 2 | 1.82 | 13.16 |
| AAANEQLTR | 0.005 | 0 | 1 | Q9NX63 | 973.506 | 65.83 | 2 | 2.34 | 13.15 |
| EAAENSLVAYK | 0.001 | 0 | 1 | P62258 | 1194.600 | 54.64 | 2 | 2.29 | 23.53 |
| YEDEINKR | 0.007 | 0 | 2 | P35908; P04264; P04259; P02538; P05787; P13647 | 1066.516 | 31.81 | 2 | 0.73 | 13.14 |
| QINDIQLSR | 0.008 | 0 | 1 | Q13347 | 1086.590 | 29.91 | 2 | -0.84 | 23.83 |
| AFGPGLQGGSAGSPAR | 0.001 | 0 | 1 | P21333 | 1429.718 | 67.3 | 2 | -0.04 | 23.85 |
| FADLSEAANR | 0.001 | 0 | 2 | P08670 | 1093.527 | 38.31 | 2 | 0.79 | 23.90 |
| DQVANSAFVER | 0.001 | 0 | 1 | P07900 | 1235.601 | 73.54 | 2 | 2.76 | 23.97 |
| RQVDQLTNDKAR | 0.003 | 0 | 1 | P08670 | 1443.766 | 36.38 | 3 | -0.76 | 13.08 |
| ATGDETGAKVER | 0.000 | 0 | 2 | P61247 | 1233.607 | 74.54 | 2 | 3.67 | 8.23 |
| QLYEEEIR | 0.014 | 0 | 1 | P05787 | 1079.537 | 51.57 | 2 | 5.11 | 23.99 |
| ELQEAQNER | 0.019 | 0 | 1 | Q9NPI1 | 1116.528 | 31.4 | 2 | 0.99 | 12.92 |
| DISTNYYASQK | 0.012 | 0 | 1 | P14625 | 1289.601 | 45.22 | 2 | 1.08 | 23.99 |
| EAALGAGFSDK | 0.001 | 0 | 1 | P55084 | 1065.521 | 49.11 | 2 | 2.11 | 23.32 |
| RQVDQLTNDK | 0.001 | 0 | 1 | P08670 | 1216.628 | 42.54 | 2 | -0.95 | 13.75 |
| HLNDDVVK | 0.016 | 0 | 1 | Q03135 | 939.489 | 27.43 | 2 | 0.01 | 13.82 |
| YSVQTADHR | 0.002 | 0 | 2 | Q16658 | 1076.512 | 35.98 | 2 | -0.85 | 13.84 |
| KDSELDKHLESR | 0.000 | 0 | 1 | O75688 | 1456.739 | 47.07 | 3 | 0.17 | 14.50 |
| YLAEFATGNDRK | 0.012 | 0 | 1 | P62258 | 1384.686 | 33.75 | 2 | -1.73 | 22.68 |
| YDPEGDNTGEQVAVK | 0.002 | 0 | 1 | P23458 | 1621.734 | 61.87 | 2 | -0.81 | 22.73 |
| LGDSHDLQR | 0.006 | 0 | 1 | Q13813 | 1040.512 | 57.11 | 2 | 1.94 | 14.34 |
| IYEYVESR | 0.012 | 0 | 1 | Q8WWY3 | 1058.515 | 20.2 | 2 | 0.00 | 22.90 |
| GSGGLGGACGGAGFGSR | 0.008 | 0 | 1 | P04259; P02538 | 1424.634 | 26.63 | 2 | -2.79 | 22.97 |
| AQAVSEDAGGNEGR | 0.010 | 0 | 1 | P55884 | 1360.609 | 26.32 | 2 | 2.37 | 14.16 |
| DNIQGITKPAIR | 0.007 | 0 | 1 | P62805 | 1325.754 | 45.89 | 2 | 0.01 | 23.01 |
| EVQVEHIK | 0.021 | 0 | 1 | Q96DG6 | 981.536 | 25.56 | 2 | 1.17 | 14.14 |
| AKPLTDSEKR | 0.004 | 0 | 1 | P11216 | 1186.643 | 24.67 | 3 | 1.14 | 14.11 |
| ALAAAGYDVEK | 0.001 | 0 | 1 | P10412 | 1107.568 | 28.7 | 2 | -0.90 | 23.02 |
| TENSTSAPAAKPK | 0.018 | 0 | 1 | P07305 | 1343.680 | 26.02 | 2 | 1.61 | 14.04 |
| ILEQQNSSR | 0.007 | 0 | 1 | Q16181 | 1074.554 | 17.68 | 2 | -0.96 | 12.90 |
| NRDSDKTDTDWR | 0.002 | 0 | 1 | P23588 | 1508.672 | 15.27 | 3 | -2.38 | 14.00 |
| FAAATGATPIAGR | 0.000 | 0 | 1 | P08865 | 1203.648 | 46.4 | 2 | 1.68 | 23.13 |
| ILGATIENSR | 0.006 | 0 | 1 | P08727 | 1073.595 | 32.98 | 2 | -3.01 | 23.13 |
| ATSSSSGSLSATGR | 0.003 | 0 | 1 | Q03252 | 1268.608 | 56.38 | 2 | -1.62 | 13.96 |
| LKPGAPLRPK | 0.006 | 0 | 1 | P21333 | 1076.694 | 22.93 | 3 | -1.16 | 13.95 |
| VDSFHESTEGK | 0.006 | 0 | 2 | Q8WWY3 | 1235.554 | 28.64 | 2 | -0.88 | 13.94 |
| EANQQQQFNR | 0.005 | 0 | 1 | Q13813 | 1262.587 | 54.32 | 2 | -1.57 | 13.91 |
| LGQEATVGK | 0.004 | 0 | 1 | P53396 | 902.494 | 48.27 | 2 | -2.62 | 13.91 |
| TNEKVELQELNDR | 0.000 | 0 | 2 | P08670 | 1587.797 | 65.27 | 2 | -0.69 | 23.14 |
| AAYEAELGDAR | 0.001 | 0 | 1 | P02545 | 1165.548 | 57.66 | 2 | 0.10 | 23.32 |
| AEGPGLSR | 0.016 | 0 | 1 | P21333 | 786.410 | 41.4 | 2 | 0.01 | 13.87 |
| QVDQLTNDKAR | 0.001 | 0 | 2 | P08670 | 1287.665 | 54.2 | 2 | -3.27 | 13.89 |
| EAVKEIQTSAK | 0.009 | 0 | 1 | Q07065 | 1203.658 | 22.35 | 2 | -1.37 | 13.84 |
| IQTQPGYANTLR | 0.014 | 0 | 1 | Q00325 | 1361.717 | 35.02 | 2 | -2.67 | 23.12 |
| EDAANNYAR | 0.002 | 0 | 1 | P68363 | 1023.449 | 48.46 | 2 | -1.42 | 12.88 |
| AQYEEIANR | 0.009 | 0 | 1 | P13647 | 1093.527 | 41.63 | 2 | -0.44 | 19.73 |
| GGNFGFGDSR | 0.011 | 0 | 1 | P22626 | 1013.444 | 35.97 | 2 | -0.85 | 24.01 |
| TPCEEILVK | 0.001 | 0 | 1 | P21333 | 1088.566 | 48.25 | 2 | 1.88 | 24.58 |
| TATPQQAQEVHEK | 0.008 | 0 | 1 | P60174 | 1466.723 | 14.65 | 3 | -0.19 | 12.88 |
| LFGAAEVQR | 0.005 | 0 | 1 | Q13813 | 990.537 | 53.23 | 2 | 1.75 | 24.73 |
| ALEESNYELEGK | 0.000 | 0 | 1 | P13645 | 1381.648 | 42.68 | 2 | 1.56 | 24.82 |
| SETAPAAPAAPAPAEK | 0.000 | 0 | 1 | P10412 | 1520.759 | 54.89 | 2 | 1.99 | 24.96 |
| AVANQTSATFLR | 0.005 | 0 | 1 | P62191 | 1278.680 | 45.18 | 2 | 1.07 | 24.97 |
| NGYGFINR | 0.012 | 0 | 1 | P67809 | 940.464 | 33.88 | 2 | 1.76 | 24.97 |
| SDLLLSGR | 0.015 | 0 | 1 | O14744 | 860.484 | 50.22 | 2 | 2.08 | 25.00 |
| NSLDCEIVSAK | 0.003 | 0 | 1 | Q01518 | 1235.594 | 34.46 | 2 | 1.42 | 25.05 |
| TALPAQSAATLPAR | 0.001 | 0 | 1 | Q01082 | 1367.764 | 69.05 | 2 | 2.28 | 25.16 |
| EAAEQDVEK | 0.017 | 0 | 1 | P26373 | 1018.469 | 23.11 | 2 | 0.77 | 9.70 |
| KLDPAQSASR | 0.009 | 0 | 1 | Q13813 | 1072.575 | 32.2 | 2 | 0.76 | 9.55 |
| GACAGSEDAVK | 0.003 | 0 | 1 | Q13813 | 1064.468 | 49.51 | 2 | 2.52 | 9.44 |
| QINLSNIR | 0.010 | 0 | 1 | Q7KZF4 | 957.548 | 19.71 | 2 | 0.50 | 25.27 |
| ADMQNLVER | 0.020 | 0 | 1 | Q01518 | 1133.526 | 22.14 | 2 | 1.41 | 25.27 |
| SQSSDTEQQSPTSGGGK | 0.000 | 0 | 3 | P23588 | 1680.731 | 114.94 | 2 | -0.71 | 9.19 |
| KFDQLLAEEK | 0.001 | 0 | 1 | P35579 | 1220.652 | 39.78 | 2 | 3.21 | 25.28 |
| EFNAEVHRK | 0.008 | 0 | 1 | P46777 | 1129.575 | 13.36 | 3 | 0.88 | 9.00 |
| NRPEDYQGGR | 0.006 | 0 | 1 | Q15084 | 1191.550 | 16.84 | 3 | 1.84 | 8.95 |
| AQIFANTVDNAR | 0.002 | 0 | 1 | P05783 | 1319.670 | 55.7 | 2 | 4.33 | 25.33 |
| GPAGPQGPR | 0.020 | 0 | 1 | P02452 | 836.437 | 24.05 | 2 | 1.07 | 8.76 |
| CAQGCICK | 0.021 | 0 | 1 | P13640 | 996.406 | 23.17 | 2 | 0.70 | 8.63 |
| TGVVPQLVK | 0.003 | 0 | 1 | P52292 | 940.583 | 42.91 | 2 | 1.40 | 25.91 |
| QEYEQLIAK | 0.008 | 0 | 1 | P35527 | 1121.584 | 53.55 | 2 | 2.24 | 25.46 |
| LQAEIEGLK | 0.006 | 0 | 1 | P05787 | 1000.567 | 41.62 | 2 | 1.50 | 25.70 |
| STMQELNSR | 0.011 | 0 | 1 | P35527 | 1081.494 | 53.77 | 2 | 2.54 | 10.68 |
| AAQEEYVKR | 0.021 | 0 | 1 | P04075 | 1093.564 | 38.82 | 2 | 2.01 | 10.77 |
| AQHEDQVEQYKK | 0.001 | 0 | 1 | P02545 | 1502.723 | 31.89 | 3 | 0.24 | 10.37 |
| VELQELNDR | 0.003 | 0 | 1 | P08670 | 1115.569 | 68.75 | 2 | 3.07 | 24.55 |
| NAESNAELK | 0.003 | 0 | 1 | P18621 | 975.474 | 33.74 | 2 | 1.19 | 10.89 |
| LVSESSDVLPK | 0.001 | 0 | 1 | P05787 | 1173.636 | 57.98 | 2 | 2.71 | 24.70 |
| IDASKNEEDEGHSNSSPR | 0.003 | 0 | 1 | Q14103 | 1971.864 | 42.89 | 3 | 2.23 | 12.28 |
| AASIFGGAKPVDTAAR | 0.005 | 0 | 1 | P23588 | 1531.823 | 38.71 | 3 | 2.32 | 24.21 |
| QSSEAEIQAK | 0.003 | 0 | 1 | Q15149 | 1090.537 | 34.08 | 2 | 0.03 | 12.63 |
| EKQPPIDNIIR | 0.001 | 0 | 4 | P52292 | 1322.743 | 40.41 | 2 | 1.79 | 24.57 |
| LTELETAVR | 0.005 | 0 | 1 | Q13561 | 1031.573 | 39.07 | 2 | 2.38 | 24.09 |
| EATTEFSVDAR | 0.001 | 0 | 1 | P21333 | 1225.570 | 48.09 | 2 | 2.58 | 24.29 |
| SAAQAAAQTNSNAAGK | 0.000 | 0 | 1 | Q8NC51 | 1460.709 | 71.1 | 2 | 0.49 | 11.96 |
| EKEPIAASTNR | 0.011 | 0 | 1 | Q13813 | 1215.633 | 25.52 | 2 | 1.56 | 11.95 |
| EDQTEYLEER | 0.017 | 0 | 1 | P07900; P08238 | 1311.570 | 44.03 | 2 | 1.36 | 24.04 |
| GSDFDCELR | 0.007 | 0 | 1 | P61978 | 1098.452 | 30.93 | 2 | -1.20 | 24.34 |
| ALGQNPTNAEVLK | 0.000 | 0 | 2 | P60660 | 1354.733 | 46.91 | 2 | 0.60 | 24.05 |
| GCGTVLLSGPR | 0.002 | 0 | 1 | Q07020 | 1116.583 | 45.22 | 2 | 0.70 | 24.43 |
| QGTEIDGR | 0.009 | 0 | 1 | P19338 | 875.422 | 52.7 | 2 | -0.13 | 11.45 |
| KGEITGEVR | 0.006 | 0 | 1 | P21333 | 988.542 | 47.89 | 2 | 1.77 | 12.55 |
| QVHPDTGISSK | 0.001 | 0 | 1 | O60814 | 1168.596 | 57.74 | 3 | -0.18 | 12.68 |
| VEPGLGADNSVVR | 0.020 | 0 | 1 | P21333 | 1312.686 | 27.86 | 2 | 2.17 | 24.50 |
| AAREPNIDR | 0.005 | 0 | 2 | P23588 | 1041.544 | 38.14 | 2 | 0.28 | 11.55 |
| AKEAQDDLVK | 0.003 | 0 | 1 | P15311 | 1116.590 | 46.57 | 2 | -0.99 | 12.28 |
| SLPFGAQSTQR | 0.002 | 0 | 1 | Q9UHL4 | 1191.612 | 24.34 | 2 | -1.47 | 24.14 |
| QEPLLIGSTK | 0.023 | 0.00153117 | 1 | P49327 | 1085.620 | 32.59 | 2 | 3.88 | 25.34 |
| GLFDEYGSK | 0.023 | 0.00153117 | 1 | P14625 | 1015.473 | 22.44 | 2 | 1.53 | 30.59 |
| EFIQEPAK | 0.024 | 0.00153117 | 1 | O14744 | 961.499 | 42.77 | 2 | 1.04 | 20.78 |
| DIELSPEAQAK | 0.023 | 0.00153117 | 1 | Q6UB35 | 1200.611 | 29.55 | 2 | 2.22 | 25.33 |
| YLYEIAR | 0.022 | 0.00153117 | 1 | P02768 | 927.493 | 16.48 | 2 | 1.20 | 27.40 |
| EISNLLVATK | 0.022 | 0.00153117 | 1 | P23458 | 1087.636 | 31.33 | 2 | 1.83 | 29.62 |
| LSLELGGK | 0.024 | 0.00153117 | 1 |  | 816.483 | 19.18 | 2 | 4.24 | 23.03 |
| ESYSVYVYK | 0.022 | 0.00153117 | 1 | O60814 | 1137.546 | 19.9 | 2 | 1.54 | 27.69 |
| FAVAALQSK | 0.024 | 0.00153117 | 1 | Q5W0B1 | 934.536 | 15.68 | 2 | 3.38 | 25.20 |
| IAPAEGPDVSER | 0.022 | 0.00153117 | 1 | Q9Y6M1 | 1240.617 | 30.48 | 2 | -0.34 | 20.17 |
| FGPGVAFR | 0.024 | 0.00153117 | 1 | P08727 | 850.457 | 44.97 | 2 | 3.90 | 27.91 |
| LIEVDDER | 0.024 | 0.00153117 | 1 | P62753 | 988.495 | 50.86 | 2 | 1.47 | 21.48 |
| AAQGEPQVQFK | 0.023 | 0.00153117 | 1 | P62826 | 1244.627 | 30.45 | 2 | -3.71 | 29.29 |
| SAINEVVTR | 0.023 | 0.00153117 | 1 | P62899 | 988.542 | 41.41 | 2 | 4.18 | 21.27 |
| ASLSLIEK | 0.022 | 0.00153117 | 1 | P52292 | 860.509 | 18.01 | 2 | 2.29 | 24.68 |
| LASYLDK | 0.023 | 0.00153117 | 1 | P08727; P13645; Q04695; P35527; P08779 | 809.440 | 43.38 | 2 | 0.46 | 20.11 |
| VPLVAPEDLR | 0.022 | 0.00153117 | 1 | O14744 | 1108.636 | 43.15 | 2 | 5.02 | 30.09 |
| ILGPGLNK | 0.022 | 0.00153117 | 1 | P62906 | 811.504 | 33.85 | 2 | 1.09 | 20.75 |
| FSGVPDR | 0.024 | 0.00153117 | 5 | A0A075B6S2 | 777.389 | 36.03 | 2 | 1.55 | 19.19 |
| SDEKAAVAGK | 0.023 | 0.00153117 | 1 | P36578 | 975.511 | 32.85 | 2 | 1.56 | 3.72 |
| IANPVEGSSGR | 0.024 | 0.00153117 | 1 | Q15365 | 1086.554 | 28.04 | 2 | -0.16 | 18.33 |
| GALALEEK | 0.024 | 0.00153117 | 1 | P35579 | 830.462 | 24.97 | 2 | 0.48 | 19.33 |
| SLETENR | 0.022 | 0.00153117 | 1 | P05783 | 848.411 | 41.68 | 2 | 0.33 | 8.35 |
| QSLGESPR | 0.024 | 0.00153117 | 1 | O14639 | 873.442 | 39.38 | 2 | 1.49 | 13.13 |
| SYKVSTSGPR | 0.023 | 0.00153117 | 2 | P05787 | 1081.564 | 32.37 | 2 | -2.71 | 13.90 |
| AFHNEAQVNPERK | 0.023 | 0.00153117 | 1 | P17987 | 1539.766 | 2.13 | 4 | 0.55 | 14.74 |
| AVPTGDASK | 0.024 | 0.00153117 | 1 | P21333 | 845.436 | 31.77 | 2 | 1.67 | 7.28 |
| ARAEEAEAQKR | 0.024 | 0.00153117 | 1 | Q15149 | 1258.650 | 31.03 | 3 | 0.66 | 4.04 |
| KAPAQKVPAQK | 0.024 | 0.00153117 | 1 | P50914 | 1165.705 | 20.35 | 3 | -0.18 | 4.04 |
| QTATQLLK | 0.023 | 0.00153117 | 1 | P62424 | 902.531 | 30.07 | 2 | 3.86 | 19.25 |
| LAHYNKR | 0.024 | 0.00153117 | 1 | O60814 | 901.500 | 23.56 | 2 | -0.17 | 1.90 |
| GDYPLEAVR | 0.040 | 0.00247552 | 1 | P14618 | 1019.516 | 33.16 | 2 | 1.71 | 26.36 |
| QGSGSGQSPSR | 0.028 | 0.00247552 | 1 | Q86YZ3 | 1047.481 | 68 | 2 | 1.63 | 0.58 |
| DYDDMSPR | 0.026 | 0.00247552 | 1 | P61978 | 1014.383 | 11.95 | 2 | 1.19 | 16.07 |
| LAEQAER | 0.037 | 0.00247552 | 1 | P63104; P31946; Q04917; P61981; P27348; P62258 | 816.421 | 35.27 | 2 | -0.45 | 7.96 |
| KLLEGEECR | 0.036 | 0.00247552 | 1 | P35908; P04259; P02538; P13647 | 1133.562 | 15.11 | 3 | 1.66 | 15.97 |
| IDKPILK | 0.039 | 0.00247552 | 1 | P62917 | 826.540 | 23.63 | 2 | 0.14 | 15.74 |
| MGESDDSILR | 0.036 | 0.00247552 | 1 | P63220 | 1138.504 | 37.82 | 2 | 2.11 | 21.44 |
| MDDREDLVYQAK | 0.026 | 0.00247552 | 1 | P62258 | 1540.695 | 22.36 | 2 | 5.83 | 26.24 |
| KAALEEVER | 0.035 | 0.00247552 | 1 | Q15149 | 1044.568 | 31.3 | 2 | 1.64 | 15.70 |
| LDQQTLPLGGR | 0.034 | 0.00247552 | 1 | P98175 | 1197.659 | 28.75 | 2 | 1.02 | 26.30 |
| NIGLGFK | 0.035 | 0.00247552 | 1 | P62280 | 748.435 | 35.4 | 2 | -0.13 | 26.40 |
| TGYTLDVTTGQR | 0.025 | 0.00247552 | 1 | O43390; O60506 | 1311.654 | 11 | 2 | -2.47 | 26.49 |
| SIYYITGESK | 0.032 | 0.00247552 | 1 | P08238 | 1160.583 | 38.98 | 2 | -0.81 | 26.48 |
| ALELEQER | 0.033 | 0.00247552 | 1 | P26038 | 987.511 | 31.86 | 2 | -0.93 | 20.97 |
| DSDKTDTDWR | 0.031 | 0.00247552 | 1 | P23588 | 1238.528 | 16.03 | 3 | 1.18 | 16.84 |
| ALELDQER | 0.036 | 0.00247552 | 1 | P35241 | 973.495 | 7.84 | 2 | 0.15 | 21.01 |
| IREHLEK | 0.026 | 0.00247552 | 1 | P05783 | 924.526 | 18.85 | 2 | 1.02 | 6.91 |
| DLPEHAVLK | 0.036 | 0.00247552 | 1 | Q00839 | 1021.568 | 27.7 | 2 | 2.21 | 21.05 |
| KELASALK | 0.029 | 0.00247552 | 1 | P07355 | 859.525 | 31.56 | 2 | 2.30 | 13.15 |
| KSYGVENR | 0.026 | 0.00247552 | 1 | Q8NFJ5 | 952.485 | 16.65 | 2 | 0.64 | 7.50 |
| VNVGAGSHPNK | 0.039 | 0.00247552 | 1 | P21333 | 1079.559 | 39.14 | 2 | -1.45 | 7.14 |
| HLQLAIR | 0.025 | 0.00247552 | 1 | Q96QV6 | 850.526 | 18.1 | 2 | 3.22 | 21.15 |
| LLEGEESR | 0.026 | 0.00247552 | 1 | P08670; P05787 | 932.468 | 40.94 | 2 | 2.25 | 16.66 |
| VQESTKGPDEAK | 0.026 | 0.00247552 | 1 | O43390 | 1288.638 | 30.37 | 2 | -2.61 | 7.16 |
| YEYQPFAGK | 0.029 | 0.00247552 | 1 | O75083 | 1102.520 | 2.16 | 2 | -1.29 | 26.61 |
| TLKPEEQR | 0.033 | 0.00247552 | 1 | Q15149 | 1000.542 | 21.79 | 2 | 1.32 | 7.34 |
| TDSDIIAK | 0.028 | 0.00247552 | 1 | P09012 | 862.452 | 27.68 | 2 | 1.35 | 16.49 |
| GQLEQITGK | 0.040 | 0.00247552 | 1 | Q5SSJ5 | 973.531 | 15.08 | 2 | 5.34 | 21.09 |
| LAPEYEAAATR | 0.038 | 0.00247552 | 1 | P30101 | 1191.600 | 26.94 | 2 | 0.38 | 21.56 |
| YSLDPENPTK | 0.028 | 0.00247552 | 1 | P18621 | 1163.558 | 14.89 | 2 | 0.96 | 25.48 |
| VEADRPGK | 0.033 | 0.00247552 | 1 | P38159 | 913.474 | 4.9 | 2 | 1.04 | 15.57 |
| EEQFNSTFR | 0.030 | 0.00247552 | 1 | P01859 | 1157.522 | 41.82 | 2 | 1.34 | 23.81 |
| GYSFTTTAER | 0.034 | 0.00247552 | 1 | P60709 | 1132.527 | 24.28 | 2 | -0.99 | 23.34 |
| AQAEQAALR | 0.025 | 0.00247552 | 1 | Q15149 | 957.511 | 35.92 | 2 | -0.13 | 13.73 |
| DTGNIGQER | 0.040 | 0.00247552 | 1 | Q01082 | 989.465 | 26.38 | 2 | -2.85 | 13.87 |
| FICTTSAIQNR | 0.030 | 0.00247552 | 1 | P53396 | 1310.652 | 27.24 | 2 | 0.93 | 23.87 |
| VTLELGGK | 0.026 | 0.00247552 | 1 | O94788 | 816.483 | 19.62 | 2 | 4.24 | 23.03 |
| ASGPGLER | 0.037 | 0.00247552 | 1 | Q14315 | 786.410 | 46.3 | 2 | 0.08 | 12.70 |
| NQDLEFER | 0.030 | 0.00247552 | 1 | P21399 | 1050.485 | 17.03 | 2 | -0.03 | 23.01 |
| TTIKDQEDLKWAFSK | 0.041 | 0.00247552 | 1 | Q8NF91 | 1809.938 | 4.64 | 4 | 1.95 | 12.67 |
| GPPASSPAPAPK | 0.040 | 0.00247552 | 1 | Q15942 | 1076.573 | 29.41 | 2 | 0.05 | 14.75 |
| CSGPGLER | 0.029 | 0.00247552 | 1 | P21333 | 875.404 | 23.44 | 2 | -0.91 | 14.26 |
| LMELHGEGSSSGK | 0.039 | 0.00247552 | 1 | P61247 | 1347.621 | 10.56 | 3 | -1.10 | 12.65 |
| YEELQVTAGR | 0.025 | 0.00247552 | 1 | P02538 | 1165.585 | 28.65 | 2 | -0.95 | 24.06 |
| AKTPVTLK | 0.039 | 0.00247552 | 1 | P42167 | 857.545 | 6.81 | 2 | 0.40 | 11.91 |
| KVGDDIAK | 0.040 | 0.00247552 | 1 | P30050 | 845.473 | 23.48 | 2 | 1.51 | 8.05 |
| ITITNDKGR | 0.025 | 0.00247552 | 1 | P11142; P0DMV9 | 1017.569 | 44.23 | 2 | 1.42 | 11.70 |
| SGYLAGDK | 0.028 | 0.00247552 | 1 | P26038 | 810.399 | 24.2 | 2 | -0.44 | 14.56 |
| QLQEDAAR | 0.025 | 0.00247552 | 1 | Q01082 | 930.464 | 40.46 | 2 | 0.77 | 10.98 |
| VMPAPPPK | 0.030 | 0.00247552 | 2 | P23588 | 852.465 | 18.57 | 2 | 0.19 | 11.13 |
| LVSLIGSK | 0.027 | 0.00247552 | 1 | P25786 | 816.519 | 36.84 | 2 | 3.04 | 24.59 |
| TIAMDGTEGLVR | 0.029 | 0.00247552 | 1 | P06576 | 1278.636 | 31.32 | 2 | 1.59 | 24.61 |
| ATDKSFVEK | 0.038 | 0.00247552 | 1 | P35579 | 1024.531 | 45.24 | 2 | 2.72 | 10.24 |
| QASEGPLK | 0.034 | 0.00247552 | 1 | P04406 | 829.441 | 60.96 | 2 | 1.16 | 10.16 |
| VPLAGAAGGPGIGR | 0.030 | 0.00247552 | 1 | P14678 | 1192.680 | 28.88 | 2 | 2.91 | 24.99 |
| AYTNFDAER | 0.037 | 0.00247552 | 1 | P07355 | 1086.485 | 16.01 | 2 | 1.21 | 21.93 |
| YVLCTAPR | 0.034 | 0.00247552 | 1 | P25205 | 979.503 | 5.38 | 2 | 4.75 | 21.83 |
| NSTPSEPGSGR | 0.029 | 0.00247552 | 1 | P51858 | 1088.497 | 35.49 | 2 | 0.95 | 9.82 |
| TYSLGSALRPSTSR | 0.025 | 0.00247552 | 1 | P08670 | 1495.786 | 9.14 | 3 | 2.46 | 25.08 |
| LVIITAGAR | 0.038 | 0.00247552 | 1 | P00338 | 913.583 | 47.23 | 2 | 2.28 | 25.30 |
| AQYDELARK | 0.029 | 0.00247552 | 1 | P05783 | 1093.564 | 24.4 | 2 | 2.79 | 16.92 |
| VQVEYKGETK | 0.030 | 0.00247552 | 2 | P11142 | 1180.621 | 41.07 | 2 | 0.47 | 14.49 |
| KVPEKPETR | 0.041 | 0.00247552 | 1 | Q15020 | 1083.616 | 44.25 | 3 | 1.76 | 6.72 |
| KDDQMLK | 0.029 | 0.00247552 | 1 | P52292 | 877.445 | 19.25 | 2 | -2.12 | 7.12 |
| VVLVLAGR | 0.027 | 0.00247552 | 1 | P61353 | 826.551 | 23.96 | 2 | 4.35 | 27.00 |
| QLFDQVVK | 0.032 | 0.00247552 | 1 | P35241; P26038; P15311 | 976.546 | 23.37 | 2 | 0.42 | 28.67 |
| VLLGETGK | 0.041 | 0.00247552 | 1 | P62280 | 816.483 | 14.12 | 2 | 0.13 | 18.56 |
| SAITPGGLR | 0.028 | 0.00247552 | 1 | P34897 | 871.500 | 39.09 | 2 | 2.69 | 20.26 |
| LELQGPR | 0.038 | 0.00247552 | 1 | P19338 | 812.462 | 42.16 | 2 | 1.92 | 20.25 |
| LVSDGNINSDRIQEK | 0.025 | 0.00247552 | 1 | Q01082 | 1687.861 | 5.55 | 3 | 0.82 | 20.87 |
| EGLELLK | 0.037 | 0.00247552 | 1 | P06733 | 801.472 | 58.49 | 2 | 1.78 | 28.68 |
| KLESTESR | 0.027 | 0.00247552 | 1 | P02545 | 949.495 | 43.83 | 2 | 0.28 | 3.06 |
| AAEFNSNLNR | 0.028 | 0.00247552 | 1 | Q8WUB8 | 1135.549 | 27.71 | 2 | 1.23 | 20.20 |
| LTPVAYGCK | 0.031 | 0.00247552 | 1 | O60825 | 1008.518 | 28.85 | 2 | 3.04 | 20.20 |
| AALEEVER | 0.031 | 0.00247552 | 1 | Q15149 | 916.473 | 6.69 | 2 | 0.93 | 18.94 |
| NVPVITGSK | 0.040 | 0.00247552 | 1 | P35232 | 914.531 | 20.75 | 2 | 1.81 | 19.00 |
| ADTLTLK | 0.038 | 0.00247552 | 1 | Q15019 | 761.440 | 21.62 | 2 | 1.38 | 19.09 |
| AFSQFGK | 0.036 | 0.00247552 | 1 | O60506 | 784.399 | 8.26 | 2 | -0.19 | 20.11 |
| LKGLALQR | 0.034 | 0.00247552 | 1 | Q13813 | 898.583 | 25.18 | 2 | 1.40 | 19.11 |
| TGFQAVTGK | 0.025 | 0.00247552 | 1 | P20042 | 908.484 | 24.63 | 2 | 2.51 | 19.18 |
| AAPGAAGSR | 0.033 | 0.00247552 | 1 | Q92522 | 757.395 | 34.33 | 2 | -0.72 | 2.28 |
| SLQSVAEER | 0.037 | 0.00247552 | 1 | P61313 | 1018.516 | 22.45 | 2 | -0.68 | 19.98 |
| VPDVQDGVR | 0.028 | 0.00247552 | 1 | Q15149 | 984.511 | 40.33 | 2 | -0.34 | 19.98 |
| TATDEAYKDPSNLQGK | 0.029 | 0.00247552 | 1 | Q13813 | 1737.829 | 23.23 | 2 | 1.36 | 19.98 |
| MDSTANEVEAVK | 0.029 | 0.00247552 | 1 | P07237 | 1309.594 | 18.02 | 2 | -1.94 | 19.43 |
| FDDGAGGDNEVQR | 0.027 | 0.00247552 | 1 | P35998 | 1379.582 | 22.44 | 2 | -4.79 | 19.55 |
| VLSPEAVR | 0.034 | 0.00247552 | 1 | O94925 | 870.504 | 42.82 | 2 | -1.78 | 19.60 |
| DLAGSIIGK | 0.034 | 0.00247552 | 1 | P61978 | 873.504 | 19.3 | 2 | 2.38 | 30.24 |
| VKQIESK | 0.031 | 0.00247552 | 1 | P10599 | 831.493 | 32.02 | 2 | -0.50 | 1.33 |
| ASLTLEEK | 0.037 | 0.00247552 | 1 | Q6P3W7 | 890.483 | 34.17 | 2 | -0.12 | 19.63 |
| APILIATDVASR | 0.034 | 0.00247552 | 1 | Q92841 | 1226.710 | 32.44 | 2 | 3.73 | 30.40 |
| KASDVHEVRK | 0.029 | 0.00247552 | 2 | P14618 | 1168.643 | 30.32 | 2 | 0.26 | 1.17 |
| KGNYAER | 0.032 | 0.00247552 | 1 | Q96QV6 | 837.421 | 44.2 | 2 | 0.04 | 0.92 |
| AEISDLDR | 0.026 | 0.00247552 | 1 | P60510 | 960.463 | 26.39 | 2 | 3.47 | 32.22 |
| LSVAAQEAAR | 0.030 | 0.00247552 | 1 | Q15149 | 1015.553 | 35.68 | 2 | -0.84 | 18.03 |
| YIFDNVAK | 0.027 | 0.00247552 | 1 | Q15293 | 969.504 | 13.7 | 2 | 3.22 | 28.62 |
| MINTDLSR | 0.034 | 0.00247552 | 1 | P36578 | 965.472 | 22.87 | 2 | 2.63 | 18.60 |
| LLEGEECR | 0.027 | 0.00247552 | 1 | P35908; P04259; P02538; P13647 | 1005.467 | 20.24 | 2 | 1.57 | 17.95 |
| NSFTPLSSSNTIR | 0.035 | 0.00247552 | 1 | O60825 | 1423.718 | 24.64 | 2 | 2.57 | 27.62 |
| RAEFTVETR | 0.034 | 0.00247552 | 1 | P21333 | 1108.575 | 18.15 | 2 | -0.91 | 17.96 |
| IQQNTFTR | 0.031 | 0.00247552 | 1 | P21333; Q14315 | 1007.527 | 18.34 | 2 | 0.76 | 17.33 |
| HGESEFNLLGK | 0.037 | 0.00247552 | 1 | O60825 | 1230.611 | 5.83 | 2 | 0.14 | 27.45 |
| GGGEQETQELASK | 0.040 | 0.00247552 | 1 | Q96QR8 | 1333.623 | 8.81 | 2 | 0.98 | 17.30 |
| ITDFQFK | 0.041 | 0.00247552 | 1 | Q70IA6 | 898.467 | 12.64 | 2 | 1.30 | 27.43 |
| TATAGPGTTK | 0.036 | 0.00247552 | 1 | P46821 | 904.473 | 9.55 | 2 | 1.35 | 4.55 |
| AGVAPLQVK | 0.029 | 0.00247552 | 1 | P21333 | 882.541 | 34.3 | 2 | 1.35 | 20.75 |
| EIQGHQPR | 0.038 | 0.00247552 | 1 | Q01082 | 964.496 | 26.15 | 2 | 1.07 | 5.83 |
| NALLSLAK | 0.035 | 0.00247552 | 1 | P04083 | 829.514 | 16.53 | 2 | 2.83 | 27.32 |
| QSACNLEKK | 0.031 | 0.00247552 | 1 | P35579 | 1077.536 | 28.18 | 3 | -1.26 | 6.08 |
| AQYDELAR | 0.033 | 0.00247552 | 1 | P05783 | 965.469 | 23.17 | 2 | 1.06 | 20.79 |
| IALLEEAR | 0.034 | 0.00247552 | 2 | P15311 | 914.531 | 31.35 | 2 | -2.86 | 27.28 |
| AISEQTGK | 0.032 | 0.00247552 | 1 | P53396 | 833.436 | 23.7 | 2 | 1.18 | 6.46 |
| IGDFGLTK | 0.026 | 0.00247552 | 1 | P23458 | 850.467 | 24.53 | 2 | -0.27 | 27.40 |
| AVVIVDDR | 0.040 | 0.00247552 | 1 | P23246 | 886.499 | 34.21 | 2 | 2.34 | 20.61 |
| AFGSGYR | 0.040 | 0.00247552 | 1 | P23588 | 757.363 | 28.23 | 2 | 0.99 | 17.35 |
| VLTEIIASR | 0.027 | 0.00247552 | 1 | P08758 | 1001.599 | 56.19 | 2 | 3.37 | 27.93 |
| NLQYYDISAK | 0.037 | 0.00247552 | 1 | P62826 | 1214.605 | 20.62 | 2 | 2.09 | 28.27 |
| EKYIDQEELNK | 0.027 | 0.00247552 | 1 | P07900; P08238 | 1408.695 | 23.55 | 2 | 6.18 | 20.50 |
| SISLYYTGEK | 0.039 | 0.00247552 | 1 | P19338 | 1160.583 | 42.37 | 2 | 2.77 | 28.35 |
| QSLEASLAETEGR | 0.043 | 0.00358622 | 1 | P13645 | 1390.681 | 42.31 | 2 | 0.77 | 29.10 |
| EGQTICVR | 0.045 | 0.00358622 | 1 | O14744 | 962.472 | 41.27 | 2 | 0.52 | 16.70 |
| EREVEER | 0.046 | 0.00358622 | 1 | P23588 | 946.459 | 22.06 | 2 | 0.97 | 3.60 |
| ALGDYDYK | 0.044 | 0.00358622 | 1 | O75688 | 944.436 | 15.13 | 2 | -0.68 | 22.99 |
| REFIQEPAK | 0.043 | 0.00358622 | 1 | O14744 | 1117.600 | 20.58 | 2 | 0.86 | 17.79 |
| INHEGEVNR | 0.043 | 0.00358622 | 1 | Q09028 | 1067.523 | 26.87 | 2 | -1.00 | 7.02 |
| GTLDPVEK | 0.045 | 0.00358622 | 1 | P11142 | 858.457 | 10.04 | 2 | -2.93 | 18.23 |
| DYDRGYDSR | 0.046 | 0.00358622 | 1 | P23588 | 1146.481 | 35.8 | 2 | 0.61 | 15.67 |
| IAPAEAPDAK | 0.042 | 0.00358622 | 1 | O00425 | 982.520 | 28.97 | 2 | 0.73 | 15.84 |
| QLQLAQEAAQK | 0.046 | 0.00358622 | 1 | Q15149 | 1227.669 | 23.09 | 2 | 1.94 | 21.55 |
| DLYDAGVKR | 0.043 | 0.00358622 | 1 | P07355 | 1036.542 | 34 | 2 | 1.86 | 19.68 |
| VNGVDDAANFR | 0.045 | 0.00358622 | 1 | O43795 | 1177.560 | 20.41 | 2 | -0.42 | 24.00 |
| ANLSLLR | 0.043 | 0.00358622 | 1 | P23246 | 786.483 | 36.38 | 2 | 3.63 | 25.37 |
| TSYAQHQQVR | 0.043 | 0.00358622 | 1 | P61247 | 1217.602 | 23.36 | 3 | -0.21 | 8.28 |
| DISENKR | 0.045 | 0.00358622 | 1 | P11142 | 861.442 | 29.76 | 2 | 0.73 | 2.72 |
| SHTILLVQPTK | 0.044 | 0.00358622 | 1 | P84090 | 1278.742 | 38.31 | 2 | -1.42 | 29.11 |
| NIILGGVK | 0.044 | 0.00358622 | 1 | P22314 | 813.519 | 11.67 | 2 | 0.76 | 24.77 |
| AVDFAER | 0.044 | 0.00358622 | 1 | P62917 | 807.400 | 29.16 | 2 | 1.49 | 19.25 |
| LASLGIK | 0.042 | 0.00358622 | 2 | Q96HS1 | 701.456 | 36.98 | 2 | -2.24 | 22.16 |
| SVSSSSYR | 0.045 | 0.00358622 | 1 | P08670 | 872.411 | 38.01 | 2 | 1.44 | 9.50 |
| ANPFGGASHAK | 0.045 | 0.00358622 | 1 | P62266 | 1056.522 | 29.29 | 2 | 0.95 | 13.18 |
| ELAEDGYSGVEVR | 0.042 | 0.00358622 | 1 | P23396 | 1423.670 | 39.91 | 2 | -0.25 | 27.46 |
| TPAQFDADELR | 0.047 | 0.00457528 | 1 | P04083 | 1262.601 | 41.96 | 2 | 3.07 | 27.75 |
| ILLWDTR | 0.048 | 0.00457528 | 1 | Q9BQA1 | 916.525 | 33.78 | 2 | 3.66 | 35.41 |
| EESGKPGAHVTVK | 0.046 | 0.00457528 | 2 | P22626 | 1338.701 | 16.31 | 2 | -0.63 | 7.44 |
| VSAQEVR | 0.050 | 0.00457528 | 1 | P15311 | 788.426 | 37.54 | 2 | 1.83 | 9.90 |
| AGGPGLER | 0.050 | 0.00457528 | 1 | P21333 | 756.400 | 25.31 | 2 | 0.74 | 12.28 |
| SPGAPGPLTLK | 0.049 | 0.00457528 | 1 | Q15942 | 1037.599 | 24 | 2 | 1.95 | 24.84 |
| KPALVAK | 0.048 | 0.00457528 | 1 | P29692; P24534 | 726.487 | 37.91 | 2 | 1.08 | 6.40 |
| TTIFSPEGR | 0.048 | 0.00457528 | 1 | P25789 | 1007.516 | 23.06 | 2 | 2.82 | 24.55 |
| IKEWYEK | 0.050 | 0.00457528 | 1 | P13645 | 995.520 | 21.61 | 2 | 0.66 | 19.74 |
| FQEAEERPK | 0.048 | 0.00457528 | 1 | Q92598 | 1133.559 | 4.23 | 2 | -9.41 | 12.96 |
| RPDQQLQGEGK | 0.047 | 0.00457528 | 1 | Q8NC51 | 1255.639 | 40.35 | 2 | 1.28 | 12.91 |
| AAQNISK | 0.048 | 0.00457528 | 1 | Q99623 | 731.405 | 36.34 | 2 | 1.11 | 1.90 |
| GGGGNFGPGPGSNFR | 0.046 | 0.00457528 | 1 | P22626 | 1377.629 | 20.62 | 2 | 3.11 | 24.64 |
| LLAEKER | 0.048 | 0.00457528 | 1 | P02545 | 858.504 | 27.62 | 2 | 1.04 | 8.25 |
| DLVAIEAK | 0.047 | 0.00457528 | 1 | Q01082 | 858.493 | 32.44 | 2 | 3.96 | 25.32 |
| VGLQVVAVK | 0.048 | 0.00457528 | 1 | P10809 | 912.588 | 23.44 | 2 | 0.89 | 25.76 |
| FANYIDKVR | 0.048 | 0.00457528 | 1 | P08670 | 1125.605 | 32.22 | 3 | 2.78 | 23.96 |
| IGVITNR | 0.050 | 0.00457528 | 1 | P62701 | 772.468 | 43.8 | 2 | -1.17 | 18.03 |
| SSTKQPTVGGTSSTPR | 0.048 | 0.00457528 | 1 | Q68CP9 | 1590.808 | 22.99 | 3 | 0.93 | 13.43 |
| EDIYSGGGGGGSR | 0.048 | 0.00457528 | 1 | Q13151 | 1211.529 | 32.29 | 2 | 2.33 | 17.28 |
| SGQGAFGNMCR | 0.050 | 0.00457528 | 1 | P36578 | 1200.488 | 28.12 | 2 | 1.83 | 15.28 |
| NMQDMVEDYR | 0.049 | 0.00457528 | 1 | P04264 | 1332.519 | 20.8 | 2 | -1.02 | 19.87 |
| GPLVNASLR | 0.050 | 0.00457528 | 1 | O14744 | 926.542 | 22.3 | 2 | 0.20 | 23.15 |
| VINVGQR | 0.048 | 0.00457528 | 1 | O60825 | 785.463 | 27.14 | 2 | 0.16 | 15.46 |
| ELEDATETADAMNR | 0.049 | 0.00457528 | 1 | P35579 | 1581.670 | 34.5 | 2 | -0.61 | 20.84 |
| LYDVETK | 0.049 | 0.00457528 | 1 | Q6P3W7 | 867.446 | 22.55 | 2 | 2.28 | 19.25 |
| ELNITAAK | 0.047 | 0.00457528 | 1 | P62081 | 859.488 | 7.92 | 2 | -3.94 | 19.64 |
| HIYYITGETK | 0.049 | 0.00457528 | 1 | P07900 | 1224.626 | 14.83 | 2 | 2.48 | 21.75 |
| SEMEVQDAELK | 0.050 | 0.00457528 | 1 | Q9UQ80 | 1294.583 | 16.07 | 2 | -1.66 | 19.94 |
| TVPVEAVTSK | 0.052 | 0.00556185 | 1 | Q14247 | 1030.578 | 26.83 | 2 | 2.75 | 19.42 |
| VLVDQTTGLSR | 0.051 | 0.00556185 | 1 | Q15717 | 1188.658 | 16.41 | 2 | 2.96 | 24.34 |
| GYADSPSK | 0.051 | 0.00556185 | 1 | Q01518 | 824.378 | 27.2 | 2 | 0.14 | 9.03 |
| ASDVHEVR | 0.051 | 0.00556185 | 1 | P14618 | 912.453 | 29.52 | 2 | -0.28 | 5.48 |
| YFSEADKIK | 0.053 | 0.00556185 | 1 | P12268 | 1100.562 | 1.82 | 3 | 0.34 | 19.98 |
| SEITELRR | 0.051 | 0.00556185 | 1 | P13645 | 1003.553 | 25.74 | 2 | 2.25 | 17.44 |
| AAQDRDQIYR | 0.051 | 0.00556185 | 1 | P62995 | 1235.613 | 19.23 | 3 | 0.80 | 14.72 |
| TAEAELSR | 0.051 | 0.00556185 | 1 | Q08378 | 876.442 | 17.46 | 2 | 1.87 | 14.76 |
| GKQFAQALR | 0.051 | 0.00556185 | 1 | O60825 | 1018.579 | 14.1 | 2 | 0.75 | 16.59 |
| NAEQYKDQADK | 0.051 | 0.00556185 | 1 | P35579 | 1309.602 | 48.39 | 2 | 1.47 | 8.77 |
| GLTSVINQK | 0.050 | 0.00556185 | 1 | P07195 | 959.552 | 27.99 | 2 | 3.53 | 22.14 |
| KQEALVAR | 0.051 | 0.00556185 | 1 | Q13813 | 914.542 | 20.26 | 2 | -0.86 | 8.18 |
| LSKEEIER | 0.051 | 0.00556185 | 1 | P0DMV9 | 1003.542 | 21.86 | 2 | 2.20 | 14.37 |
| VEEIMEK | 0.052 | 0.00556185 | 1 | O75688 | 877.434 | 24.35 | 2 | -0.17 | 17.59 |
| HVLVTLGEK | 0.051 | 0.00556185 | 1 | P60660 | 995.588 | 23.8 | 2 | 1.37 | 20.77 |
| GEGQLGPAER | 0.052 | 0.00556185 | 1 | P13639 | 1013.501 | 19.62 | 2 | 0.52 | 16.22 |
| STELLIR | 0.053 | 0.00556185 | 1 | P68431 | 831.493 | 38.48 | 2 | 1.78 | 23.63 |
| FTPVASK | 0.053 | 0.00556185 | 1 | Q15942 | 749.419 | 26.74 | 2 | 1.90 | 14.16 |
| LISISGK | 0.053 | 0.00556185 | 2 | P06748 | 717.451 | 38.87 | 2 | 2.00 | 21.07 |
| DTLYEAVR | 0.054 | 0.00660728 | 1 | P62906 | 966.489 | 26.47 | 2 | 2.31 | 25.28 |
| STVHEILCK | 0.053 | 0.00660728 | 1 | P07355 | 1128.572 | 19.53 | 2 | 0.05 | 26.76 |
| FVSFLGR | 0.054 | 0.00660728 | 1 | P52292 | 825.462 | 10.47 | 2 | 2.84 | 32.11 |
| LTICPQAENR | 0.053 | 0.00660728 | 1 | Q9ULW8 | 1201.599 | 38.15 | 2 | 0.63 | 21.77 |
| IKETLEK | 0.053 | 0.00660728 | 1 | P13533 | 860.509 | 14.93 | 2 | 0.80 | 11.43 |
| VGVNGFGR | 0.054 | 0.00660728 | 1 | P04406 | 805.432 | 39.08 | 2 | 2.26 | 21.08 |
| GLNISAVR | 0.054 | 0.00660728 | 1 | P23588 | 829.489 | 34.42 | 2 | 1.07 | 23.00 |
| ISSSSFSR | 0.054 | 0.00749378 | 1 | P05787 | 870.432 | 19.44 | 2 | 0.69 | 17.62 |
| TAENATSGETLEENEAGD | 0.054 | 0.00749378 | 1 | Q9UQ80 | 1837.757 | 17.91 | 2 | 2.26 | 25.16 |
| VAQLLER | 0.056 | 0.00749378 | 1 | Q15149 | 828.494 | 31.07 | 2 | 1.67 | 20.47 |
| QESTEYR | 0.055 | 0.00749378 | 1 | P08670 | 912.406 | 34.61 | 2 | 1.33 | 7.82 |
| NTVVPTKK | 0.056 | 0.00749378 | 1 | P11021 | 886.536 | 40.27 | 2 | 1.36 | 6.66 |
| LYDIDVAK | 0.056 | 0.00749378 | 1 | P62750 | 936.504 | 14.4 | 2 | 3.10 | 27.08 |
| VINEEYK | 0.057 | 0.00749378 | 1 | Q09028 | 894.457 | 24.92 | 2 | 2.17 | 15.70 |
| LQDEIQNMKEEMAR | 0.055 | 0.00749378 | 1 | P08670 | 1766.805 | 7.56 | 3 | 1.05 | 17.15 |
| NKPGVYTK | 0.055 | 0.00749378 | 2 | Q9BYE2 | 906.504 | 26.79 | 2 | 0.04 | 5.93 |
| EQSQLTATQTR | 0.054 | 0.00749378 | 1 | Q6P2Q9 | 1262.634 | 9.3 | 2 | 0.93 | 15.59 |
| GGSGGSYGR | 0.059 | 0.00749378 | 1 | P35527 | 797.354 | 52.7 | 2 | -0.27 | 3.17 |
| TDTGEPMGR | 0.058 | 0.00749378 | 1 | P07900 | 979.415 | 19.5 | 2 | 0.59 | 7.26 |
| TGIYEEK | 0.057 | 0.00749378 | 1 | P27824 | 839.415 | 20.38 | 2 | 0.09 | 15.10 |
| AHATTSNTVSK | 0.057 | 0.00749378 | 1 | Q6NZI2 | 1116.564 | 23.52 | 2 | 1.09 | 1.46 |
| TTPSVVAFTADGER | 0.055 | 0.00749378 | 1 | P38646 | 1450.717 | 28.49 | 2 | -0.87 | 29.64 |
| AEYDLTTR | 0.057 | 0.00749378 | 1 | P60228 | 1010.479 | 11.58 | 2 | 2.55 | 30.32 |
| DFNPTATVK | 0.059 | 0.00749378 | 1 | P19367 | 992.505 | 0.37 | 2 | -0.11 | 23.90 |
| TAAQLKK | 0.058 | 0.00749378 | 1 | P26640 | 759.472 | 2.34 | 2 | 0.67 | 0.80 |
| IQLQDAGR | 0.059 | 0.00749378 | 1 | Q9H936 | 900.490 | 7.03 | 2 | -0.70 | 18.80 |
| KIFVGTK | 0.058 | 0.00749378 | 1 | P62701 | 792.498 | 33.2 | 2 | 0.45 | 13.12 |
| TTAQVLIR | 0.059 | 0.00852868 | 1 | C9JRZ8 | 901.547 | 26.37 | 2 | 1.23 | 20.69 |
| FTGSEIR | 0.059 | 0.00852868 | 1 | P31930 | 809.415 | 17.58 | 2 | 2.51 | 17.24 |
| VLSIGDGIAR | 0.059 | 0.00852868 | 1 | P25705 | 1000.579 | 33.4 | 2 | 1.92 | 28.17 |
| NLDDGIDDER | 0.063 | 0.0091144 | 1 | P11940 | 1161.502 | 12.98 | 2 | 0.67 | 22.83 |
| PGPTPSGTNVGSSGR | 0.066 | 0.0091144 | 1 | P60468 | 1370.666 | 36.64 | 2 | 0.31 | 16.57 |
| RDQALTEEHAR | 0.061 | 0.0091144 | 1 | P12814 | 1325.656 | 10.54 | 3 | -0.83 | 8.09 |
| AGGTGLER | 0.065 | 0.0091144 | 1 | Q14315 | 760.395 | 35.5 | 2 | 1.24 | 8.08 |
| KFDDFQK | 0.064 | 0.0091144 | 1 | Q13813 | 927.457 | 24.46 | 2 | 1.01 | 17.24 |
| VGSSNFR | 0.069 | 0.0091144 | 1 | P05787 | 766.384 | 46.73 | 2 | -0.11 | 12.66 |
| NSLPDTVQIR | 0.068 | 0.0091144 | 1 | P56537 | 1142.616 | 27.14 | 2 | -0.78 | 26.14 |
| RPELEDSTLR | 0.063 | 0.0091144 | 1 | Q15149 | 1215.633 | 22.69 | 2 | -1.05 | 19.65 |
| ALQSGPPQSR | 0.063 | 0.0091144 | 1 | O00425 | 1040.548 | 39 | 2 | 0.76 | 13.97 |
| IEEELGSK | 0.067 | 0.0091144 | 1 | P06733 | 904.462 | 23.95 | 2 | 0.54 | 16.43 |
| SITNTTVCTK | 0.060 | 0.0091144 | 1 | Q15637 | 1124.562 | 13.84 | 2 | 1.71 | 16.59 |
| DISTNYYASQKK | 0.064 | 0.0091144 | 1 | P14625 | 1417.696 | 1.35 | 2 | 3.66 | 19.81 |
| SFVLNLGK | 0.066 | 0.0091144 | 1 | P09382 | 877.514 | 11.96 | 2 | 5.32 | 31.79 |
| LKDDEVAQLKK | 0.068 | 0.0091144 | 1 | P07195 | 1286.731 | 13.25 | 3 | 0.79 | 16.30 |
| ALTQTGGPHVK | 0.059 | 0.0091144 | 1 | P21333 | 1108.611 | 19.73 | 2 | 0.07 | 13.12 |
| VKAEGPGLSR | 0.068 | 0.0091144 | 1 | P21333 | 1013.574 | 30.57 | 2 | 0.56 | 13.61 |
| SALALAIK | 0.069 | 0.0091144 | 1 | P25789 | 786.508 | 8.87 | 2 | 2.31 | 26.15 |
| MQASIEK | 0.068 | 0.0091144 | 1 | P06748 | 822.403 | 17.8 | 2 | -0.53 | 7.07 |
| TLTPLGR | 0.064 | 0.0091144 | 1 | Q96HS1 | 757.457 | 12.46 | 2 | 1.03 | 20.68 |
| FANYIDK | 0.061 | 0.0091144 | 1 | P08670 | 870.436 | 38.11 | 2 | -0.84 | 22.11 |
| TVEVAEGEAVR | 0.069 | 0.0091144 | 1 | Q86UE4 | 1159.595 | 36.75 | 2 | 3.30 | 20.23 |
| VDPVNFK | 0.060 | 0.0091144 | 1 | P02008 | 818.441 | 22.54 | 2 | 2.06 | 21.99 |
| LAQYESK | 0.068 | 0.0091144 | 1 | P24534 | 838.431 | 17.26 | 2 | -0.20 | 10.35 |
| ELASALK | 0.062 | 0.0091144 | 1 | P07355 | 731.430 | 36.76 | 2 | 1.27 | 17.68 |
| STAGDTHLGGEDFDNR | 0.066 | 0.0091144 | 1 | P11142 | 1691.726 | 35.81 | 2 | 1.40 | 21.83 |
| VNIVPVIAK | 0.067 | 0.0091144 | 1 | Q15019 | 952.619 | 26.82 | 2 | 1.05 | 28.80 |
| TAGINVR | 0.061 | 0.0091144 | 1 | P50991 | 730.421 | 30.87 | 2 | 1.29 | 14.93 |
| ALSTGEKGFGYK | 0.060 | 0.0091144 | 1 | P62937 | 1257.647 | 38.41 | 2 | 0.98 | 20.22 |
| VSADNTVGR | 0.066 | 0.0091144 | 1 | O00303 | 918.464 | 16.28 | 2 | 1.57 | 12.15 |
| ELSGTIK | 0.068 | 0.0091144 | 1 | P30050 | 747.425 | 26.75 | 2 | 0.62 | 14.70 |
| GLGLDESGLAK | 0.063 | 0.0091144 | 1 | Q9Y265 | 1059.568 | 22.14 | 2 | 3.32 | 27.83 |
| AAKPTTTPEVK | 0.066 | 0.0091144 | 1 | P46821 | 1142.642 | 26.75 | 3 | 1.26 | 8.95 |
| LTLLNPK | 0.069 | 0.0091144 | 1 | P53396 | 798.508 | 23.18 | 2 | -0.79 | 24.43 |
| DLEGLSQR | 0.064 | 0.0091144 | 1 | P35579 | 917.469 | 38.8 | 2 | -0.81 | 22.70 |
| LSVDYGKK | 0.069 | 0.0091144 | 1 | P68363 | 909.504 | 38.97 | 2 | 0.75 | 15.44 |
| LASYLDR | 0.067 | 0.0091144 | 1 | P05783 | 837.446 | 46.81 | 2 | 3.02 | 21.70 |
| LSVDYGK | 0.066 | 0.0091144 | 1 | P68363 | 781.409 | 25.89 | 2 | -0.78 | 19.61 |
| NAMGSLASQATK | 0.065 | 0.0091144 | 1 | P55036 | 1194.578 | 11.87 | 2 | 0.29 | 15.54 |
| EKEAAEQAER | 0.071 | 0.00984184 | 1 | Q969G3 | 1160.554 | 39.87 | 2 | -1.02 | 4.29 |
| LKSTVTK | 0.074 | 0.00984184 | 1 | Q6P3W7 | 776.488 | 24.67 | 2 | 0.51 | 2.21 |
| KHEDFEK | 0.073 | 0.00984184 | 1 | Q13813 | 932.447 | 17.9 | 2 | 0.56 | 2.84 |
| DDEVQVVR | 0.074 | 0.00984184 | 1 | P61254 | 959.479 | 20.56 | 2 | 0.24 | 19.81 |
| TKFETEQALR | 0.072 | 0.00984184 | 2 | P08727; Q04695 | 1222.643 | 43.42 | 3 | 1.71 | 18.73 |
| SEITELR | 0.074 | 0.00984184 | 1 | P13645 | 847.452 | 29.34 | 2 | 2.50 | 20.55 |
| AGFAGDQIPK | 0.073 | 0.00984184 | 1 | P42025 | 1003.521 | 9.03 | 2 | -2.23 | 23.46 |
| ENTQTTIK | 0.072 | 0.00984184 | 1 | P61978 | 934.484 | 59.37 | 2 | -0.14 | 7.55 |
| EVAGHTEQLQMSR | 0.073 | 0.00984184 | 1 | P08727 | 1501.706 | 5.82 | 2 | 4.78 | 15.30 |
| SQSAAVTPSSTTSSTR | 0.070 | 0.00984184 | 1 | Q16186 | 1567.756 | 3.13 | 2 | 0.68 | 15.42 |
| DKPTNNFPK | 0.075 | 0.00984184 | 1 | O60825 | 1060.542 | 6.15 | 3 | 0.60 | 14.07 |
| LQLELSK | 0.074 | 0.00984184 | 1 | P02545 | 830.498 | 31.21 | 2 | 3.04 | 25.30 |
| VTNDNPECR | 0.070 | 0.00984184 | 1 | O00622 | 1104.474 | 32.05 | 2 | -0.15 | 9.32 |
| FIEIAAR | 0.072 | 0.00984184 | 1 | Q00839 | 819.472 | 26.32 | 2 | 3.83 | 24.73 |
| IAGQVAAANK | 0.070 | 0.00984184 | 1 | P39019 | 942.537 | 7 | 2 | -2.05 | 12.06 |
| NPADLPK | 0.070 | 0.00984184 | 1 | P13639 | 754.409 | 34.79 | 2 | 1.66 | 15.61 |
| DAQAIFER | 0.070 | 0.00984184 | 1 | O15541 | 949.474 | 27.3 | 2 | 2.93 | 26.43 |
| TKTEISEMNR | 0.072 | 0.00984184 | 2 | P05787 | 1224.589 | 16.21 | 2 | -0.07 | 7.52 |
| IIAPPERK | 0.074 | 0.00984184 | 1 | P60709 | 923.567 | 17.02 | 2 | -0.63 | 13.96 |
| EAEGQLQK | 0.073 | 0.00984184 | 1 | Q15149 | 902.458 | 28.08 | 2 | 2.39 | 8.05 |
| FGPALSVK | 0.072 | 0.00984184 | 1 | P11940 | 818.477 | 22.8 | 2 | 3.55 | 25.27 |
| ESNVQEVR | 0.074 | 0.00984184 | 1 | P62714 | 960.474 | 16.82 | 2 | 2.96 | 10.54 |
| ILTTEGR | 0.090 | 0.00991222 | 1 | Q08211 | 789.446 | 24.34 | 2 | 1.66 | 14.70 |
| ITITNDK | 0.092 | 0.00991222 | 1 | P11142; P0DMV9 | 804.446 | 26.38 | 2 | 0.45 | 14.59 |
| SEETQERER | 0.085 | 0.00991222 | 1 | P23588 | 1163.529 | 17.43 | 3 | 0.26 | 2.43 |
| INVYYNEATGNK | 0.090 | 0.00991222 | 1 | Q9BVA1 | 1385.670 | 18.27 | 2 | 4.43 | 24.69 |
| TPVEVPVGGFK | 0.078 | 0.00991222 | 1 | Q15149 | 1129.625 | 21.09 | 2 | -1.64 | 29.03 |
| IGGAQNR | 0.078 | 0.00991222 | 1 | P14174 | 715.385 | 20.64 | 2 | 1.46 | 2.36 |
| SVNELIYK | 0.087 | 0.00991222 | 1 | P18124 | 965.530 | 9.96 | 2 | 2.63 | 24.91 |
| DSQDGSSYR | 0.093 | 0.00991222 | 1 | Q14847 | 1014.412 | 12.8 | 2 | 1.61 | 10.20 |
| SALDQYR | 0.080 | 0.00991222 | 1 | Q15149 | 852.421 | 21.92 | 2 | 2.51 | 20.28 |
| LAADDFR | 0.087 | 0.00991222 | 1 | P08727; P13645; P05783; Q04695; P08779 | 807.400 | 21.74 | 2 | 0.73 | 22.89 |
| VTVPLVR | 0.092 | 0.00991222 | 1 | Q9UKM9 | 783.509 | 24.03 | 2 | 3.60 | 24.60 |
| LRDLEDSLAR | 0.077 | 0.00991222 | 1 | P02545 | 1187.638 | 20.24 | 3 | 4.63 | 24.53 |
| AVAAATYK | 0.085 | 0.00991222 | 1 | Q15020 | 794.441 | 22.64 | 2 | 0.51 | 12.01 |
| MDLLLTK | 0.094 | 0.00991222 | 1 | Q6P3W7 | 849.475 | 4.31 | 2 | -1.65 | 24.50 |
| IQNAGGSVMIQR | 0.087 | 0.00991222 | 1 | O75688 | 1273.668 | 16.65 | 2 | 1.92 | 21.54 |
| SILPTAPR | 0.078 | 0.00991222 | 1 | P23588 | 854.509 | 30.74 | 2 | 3.02 | 23.78 |
| SEETQER | 0.077 | 0.00991222 | 1 | P23588 | 878.385 | 11.29 | 2 | 1.16 | 0.64 |
| VTPDTDWAR | 0.090 | 0.00991222 | 1 | P53396 | 1060.506 | 18.89 | 2 | 6.43 | 23.94 |
| ILENAQR | 0.084 | 0.00991222 | 1 | P42224 | 843.468 | 14.98 | 2 | -0.99 | 13.97 |
| NAQKIDR | 0.092 | 0.00991222 | 1 | Q96DG6 | 844.464 | 27.14 | 2 | 0.88 | 0.91 |
| ASGVAVSDGVIK | 0.094 | 0.00991222 | 1 | P23528 | 1144.621 | 44.93 | 2 | -5.81 | 31.45 |
| EIHQFNR | 0.079 | 0.00991222 | 1 | Q01082 | 943.474 | 16.13 | 2 | 1.33 | 13.12 |
| KVQAAQSEAK | 0.092 | 0.00991222 | 1 | Q16891 | 1059.579 | 29.66 | 2 | -1.75 | 1.06 |
| AVTTPGKK | 0.085 | 0.00991222 | 1 | P19338 | 801.483 | 22.89 | 2 | -0.28 | 1.19 |
| ASNVTNK | 0.079 | 0.00991222 | 1 | P07910 | 775.394 | 27.58 | 2 | 0.47 | 14.09 |
| LITDLQDQNQK | 0.080 | 0.00991222 | 1 | P33176 | 1315.685 | 21.16 | 2 | -4.45 | 22.88 |
| EAALSTALSEK | 0.077 | 0.00991222 | 1 | P02545 | 1119.589 | 30.96 | 2 | 2.37 | 24.17 |
| TFITQQGIK | 0.084 | 0.00991222 | 1 | Q96CW1 | 1035.583 | 6.75 | 2 | 3.87 | 24.25 |
| ANVGAGKKPKE | 0.087 | 0.00991222 | 1 | P62847 | 1098.627 | 5.14 | 3 | 1.65 | 1.71 |
| ISRPGDSDDSR | 0.088 | 0.00991222 | 1 | P14866 | 1204.555 | 27.82 | 2 | 1.08 | 9.89 |
| YAEAVTR | 0.085 | 0.00991222 | 1 | P00558 | 809.415 | 14.15 | 2 | 1.45 | 14.45 |
| AVASEIFK | 0.093 | 0.00991222 | 1 | O00159 | 864.483 | 15.62 | 2 | 2.88 | 24.53 |
| VKESITR | 0.080 | 0.00991222 | 1 | P21333 | 832.489 | 16.46 | 2 | 1.47 | 7.23 |
| TIAQDYGVLK | 0.093 | 0.00991222 | 1 | Q06830 | 1107.604 | 17.22 | 2 | -2.79 | 28.25 |
| IMATPEQVGK | 0.079 | 0.00991222 | 1 | P12956 | 1089.561 | 13.75 | 2 | 1.54 | 17.49 |
| GVVEVTHDLQK | 0.087 | 0.00991222 | 1 | P50454 | 1224.658 | 10.84 | 2 | 3.17 | 21.34 |
| SSFSQHAR | 0.076 | 0.00991222 | 1 | P02545 | 919.438 | 38.39 | 2 | 0.71 | 6.56 |
| RQLETLGQEK | 0.090 | 0.00991222 | 1 | P05787 | 1201.654 | 36.8 | 2 | 2.51 | 16.07 |
| VDIAEGR | 0.092 | 0.00991222 | 1 | Q15056 | 759.400 | 48.94 | 2 | -0.19 | 16.14 |
| CNTCGEPITDR | 0.087 | 0.00991222 | 1 | Q15942 | 1322.546 | 18.68 | 2 | -0.78 | 17.12 |
| SGNFGGSR | 0.091 | 0.00991222 | 1 | P22626 | 781.359 | 26.21 | 2 | -1.39 | 6.80 |
| YLAEVASGEKK | 0.088 | 0.00991222 | 1 | Q04917 | 1194.636 | 3.66 | 2 | 3.61 | 16.80 |
| SVIISIK | 0.091 | 0.00991222 | 2 | Q0IIM8 | 759.497 | 23.22 | 2 | 1.39 | 26.90 |
| DISSIGLK | 0.077 | 0.00991222 | 1 | Q86VP6 | 832.477 | 7.94 | 2 | -0.36 | 26.87 |
| SEVDMLK | 0.085 | 0.00991222 | 1 | P07355 | 837.402 | 6.9 | 2 | 1.56 | 16.80 |
| VASQGEVVR | 0.088 | 0.00991222 | 1 | P07814 | 944.516 | 26.45 | 2 | -0.70 | 13.34 |
| FASFIDK | 0.092 | 0.00991222 | 1 | P35908; P04259; P02538; P05787; P13647 | 827.430 | 35.73 | 2 | -0.28 | 26.75 |
| LSGIEER | 0.083 | 0.00991222 | 1 | Q01082 | 803.426 | 49.18 | 2 | -0.06 | 16.30 |
| FEGLTAR | 0.077 | 0.00991222 | 1 | Q15208 | 793.420 | 19.32 | 2 | 2.53 | 21.27 |
| DYSSGFGGK | 0.082 | 0.00991222 | 1 | Q14247 | 917.400 | 3.58 | 2 | 3.61 | 21.17 |
| GANLKDYYSR | 0.080 | 0.00991222 | 1 | Q08211 | 1186.585 | 16.31 | 2 | -1.42 | 20.83 |
| VAQPTITDNK | 0.077 | 0.00991222 | 1 | P21333 | 1086.579 | 5.94 | 2 | 0.06 | 17.15 |
| HWHMDHFCCFECEASLGGQR | 0.090 | 0.00991222 | 1 | O43900 | 2580.006 | 5.27 | 2 | 2.70 | 25.97 |
| HPNIVSLQDVLMQDSR | 0.092 | 0.00991222 | 1 | P06493 | 1867.933 | 1.33 | 2 | 1.35 | 20.76 |
| LVTDLTK | 0.084 | 0.00991222 | 1 | P02768 | 789.472 | 37.29 | 2 | 2.50 | 20.48 |
| VLVEDPERPACAPAAPRLQMHHVAQVLR | 0.094 | 0.00991222 | 1 | O94759 | 3176.657 | 1.45 | 3 | 2.89 | 7.24 |
| DISLSDYK | 0.088 | 0.00991222 | 1 | Q06830 | 940.462 | 21.51 | 2 | 3.31 | 28.06 |
| FGTSETSK | 0.084 | 0.00991222 | 1 | P49792 | 856.405 | 13.64 | 2 | 1.17 | 9.35 |
| EQTEILESSR | 0.093 | 0.00991222 | 1 | Q5T200 | 1191.585 | 27.85 | 2 | 2.32 | 20.62 |
| QAKQESTEYRR | 0.093 | 0.00991222 | 1 | P08670 | 1395.698 | 12.84 | 3 | 0.13 | 4.86 |
| GTQSIPNDSPAR | 0.076 | 0.00991222 | 1 | Q9NQ92 | 1242.607 | 16.54 | 2 | 0.62 | 17.40 |
| EAPAPPKAEAK | 0.092 | 0.00991222 | 1 | P62750 | 1108.600 | 33.49 | 2 | 2.27 | 9.74 |
| TIAPCQK | 0.092 | 0.00991222 | 1 | P38646 | 817.424 | 9.94 | 2 | -1.81 | 8.50 |
| AFEDEKK | 0.077 | 0.00991222 | 1 | Q9UQ80 | 866.425 | 23.7 | 2 | 0.40 | 5.41 |
| AQFEQLKDGK | 0.086 | 0.00991222 | 1 | Q15149 | 1163.606 | 18.49 | 3 | 0.83 | 17.39 |
| TLLADQGEIR | 0.087 | 0.00991222 | 1 | O94776 | 1115.606 | 35.87 | 2 | 5.25 | 25.46 |
| GFPVLSK | 0.089 | 0.00991222 | 1 | O14744 | 747.440 | 23.04 | 2 | 1.44 | 25.66 |
| LTPEEIER | 0.081 | 0.00991222 | 2 | P11021 | 986.515 | 24.33 | 2 | 2.85 | 21.63 |
| PENVAPR | 0.078 | 0.00991222 | 1 | P50991 | 782.416 | 37.81 | 2 | 0.22 | 8.14 |
| LGLTEIR | 0.081 | 0.00991222 | 1 | Q8WWY3 | 801.483 | 36.09 | 2 | 2.46 | 25.84 |
| LPLQDVYK | 0.078 | 0.00991222 | 1 | P68104 | 975.551 | 13.11 | 2 | 6.19 | 27.66 |
| FSELTAEK | 0.087 | 0.00991222 | 1 | P48643 | 924.467 | 19.92 | 2 | 1.24 | 19.70 |
