# Supplementary material for: KPNA2 promotes renal cell carcinoma proliferation and metastasis via NPM
Source: J Cell Mol Med. 2021 Sep 1;25(19):9255–67. doi: 10.1111/jcmm.16846 (PMC8500977; doi:10.1111/jcmm.16846)
Supplement: Supplementary file 8 — Table S3 [file JCMM-25-9255-s008.docx]

| **Supplementray table3 specific proteins identified in ACHN cells infected by KPNA2-overexpressed (OE)** | | | | | | | |
| --- | --- | --- | --- | --- | --- | --- | --- |
| Accession | Gene Name | Description | Coverage | # Unique Peptides | # AAs | MW [kDa] | calc. pI |
| P31946 | YWHAB | 14-3-3 protein beta/alpha OS=Homo sapiens OX=9606 GN=YWHAB PE=1 SV=3 | 16.260 | 2 | 246 | 28.065 | 4.83 |
| P62258 | YWHAE | 14-3-3 protein epsilon OS=Homo sapiens OX=9606 GN=YWHAE PE=1 SV=1 | 19.608 | 3 | 255 | 29.155 | 4.74 |
| Q04917 | YWHAH | 14-3-3 protein eta OS=Homo sapiens OX=9606 GN=YWHAH PE=1 SV=4 | 14.228 | 2 | 246 | 28.201 | 4.84 |
| P61981 | YWHAG | 14-3-3 protein gamma OS=Homo sapiens OX=9606 GN=YWHAG PE=1 SV=2 | 19.433 | 3 | 247 | 28.285 | 4.89 |
| P27348 | YWHAQ | 14-3-3 protein theta OS=Homo sapiens OX=9606 GN=YWHAQ PE=1 SV=1 | 11.837 | 1 | 245 | 27.747 | 4.78 |
| P63104 | YWHAZ | 14-3-3 protein zeta/delta OS=Homo sapiens OX=9606 GN=YWHAZ PE=1 SV=1 | 11.837 | 1 | 245 | 27.728 | 4.79 |
| Q13200 | PSMD2 | 26S proteasome non-ATPase regulatory subunit 2 OS=Homo sapiens OX=9606 GN=PSMD2 PE=1 SV=3 | 1.652 | 1 | 908 | 100.136 | 5.2 |
| P55036 | PSMD4 | 26S proteasome non-ATPase regulatory subunit 4 OS=Homo sapiens OX=9606 GN=PSMD4 PE=1 SV=1 | 3.183 | 1 | 377 | 40.711 | 4.79 |
| P62191 | PSMC1 | 26S proteasome regulatory subunit 4 OS=Homo sapiens OX=9606 GN=PSMC1 PE=1 SV=1 | 2.727 | 1 | 440 | 49.154 | 6.21 |
| P35998 | PSMC2 | 26S proteasome regulatory subunit 7 OS=Homo sapiens OX=9606 GN=PSMC2 PE=1 SV=3 | 3.002 | 1 | 433 | 48.603 | 5.95 |
| P62195 | PSMC5 | 26S proteasome regulatory subunit 8 OS=Homo sapiens OX=9606 GN=PSMC5 PE=1 SV=1 | 3.202 | 1 | 406 | 45.597 | 7.55 |
| P62280 | RPS11 | 40S ribosomal protein S11 OS=Homo sapiens OX=9606 GN=RPS11 PE=1 SV=3 | 9.494 | 2 | 158 | 18.419 | 10.3 |
| P62263 | RPS14 | 40S ribosomal protein S14 OS=Homo sapiens OX=9606 GN=RPS14 PE=1 SV=3 | 7.285 | 1 | 151 | 16.263 | 10.05 |
| P15880 | RPS2 | 40S ribosomal protein S2 OS=Homo sapiens OX=9606 GN=RPS2 PE=1 SV=2 | 3.754 | 1 | 293 | 31.305 | 10.24 |
| P63220 | RPS21 | 40S ribosomal protein S21 OS=Homo sapiens OX=9606 GN=RPS21 PE=1 SV=1 | 12.048 | 1 | 83 | 9.106 | 8.5 |
| P62266 | RPS23 | 40S ribosomal protein S23 OS=Homo sapiens OX=9606 GN=RPS23 PE=1 SV=3 | 7.692 | 1 | 143 | 15.798 | 10.49 |
| P62847 | RPS24 | 40S ribosomal protein S24 OS=Homo sapiens OX=9606 GN=RPS24 PE=1 SV=1 | 8.271 | 1 | 133 | 15.413 | 10.78 |
| P23396 | RPS3 | 40S ribosomal protein S3 OS=Homo sapiens OX=9606 GN=RPS3 PE=1 SV=2 | 5.350 | 1 | 243 | 26.671 | 9.66 |
| P62701 | RPS4X | 40S ribosomal protein S4, X isoform OS=Homo sapiens OX=9606 GN=RPS4X PE=1 SV=2 | 9.125 | 3 | 263 | 29.579 | 10.15 |
| P62081 | RPS7 | 40S ribosomal protein S7 OS=Homo sapiens OX=9606 GN=RPS7 PE=1 SV=1 | 4.124 | 1 | 194 | 22.113 | 10.1 |
| P08865 | RPSA | 40S ribosomal protein SA OS=Homo sapiens OX=9606 GN=RPSA PE=1 SV=4 | 4.407 | 1 | 295 | 32.833 | 4.87 |
| Q9H0D6 | XRN2 | 5'-3' exoribonuclease 2 OS=Homo sapiens OX=9606 GN=XRN2 PE=1 SV=1 | 1.263 | 1 | 950 | 108.513 | 7.47 |
| P05388 | RPLP0 | 60S acidic ribosomal protein P0 OS=Homo sapiens OX=9606 GN=RPLP0 PE=1 SV=1 | 3.470 | 1 | 317 | 34.252 | 5.97 |
| P30050 | RPL12 | 60S ribosomal protein L12 OS=Homo sapiens OX=9606 GN=RPL12 PE=1 SV=1 | 14.545 | 3 | 165 | 17.808 | 9.42 |
| P50914 | RPL14 | 60S ribosomal protein L14 OS=Homo sapiens OX=9606 GN=RPL14 PE=1 SV=4 | 5.116 | 1 | 215 | 23.417 | 10.93 |
| P18621 | RPL17 | 60S ribosomal protein L17 OS=Homo sapiens OX=9606 GN=RPL17 PE=1 SV=3 | 10.326 | 2 | 184 | 21.383 | 10.17 |
| Q07020 | RPL18 | 60S ribosomal protein L18 OS=Homo sapiens OX=9606 GN=RPL18 PE=1 SV=2 | 11.170 | 2 | 188 | 21.621 | 11.72 |
| P62750 | RPL23A | 60S ribosomal protein L23a OS=Homo sapiens OX=9606 GN=RPL23A PE=1 SV=1 | 12.179 | 2 | 156 | 17.684 | 10.45 |
| P61254 | RPL26 | 60S ribosomal protein L26 OS=Homo sapiens OX=9606 GN=RPL26 PE=1 SV=1 | 5.517 | 1 | 145 | 17.248 | 10.55 |
| P61353 | RPL27 | 60S ribosomal protein L27 OS=Homo sapiens OX=9606 GN=RPL27 PE=1 SV=2 | 5.882 | 1 | 136 | 15.788 | 10.56 |
| P61927 | RPL37 | 60S ribosomal protein L37 OS=Homo sapiens OX=9606 GN=RPL37 PE=1 SV=2 | 9.278 | 1 | 97 | 11.071 | 11.74 |
| P46777 | RPL5 | 60S ribosomal protein L5 OS=Homo sapiens OX=9606 GN=RPL5 PE=1 SV=3 | 3.030 | 1 | 297 | 34.341 | 9.72 |
| Q02878 | RPL6 | 60S ribosomal protein L6 OS=Homo sapiens OX=9606 GN=RPL6 PE=1 SV=3 | 4.514 | 1 | 288 | 32.708 | 10.58 |
| P18124 | RPL7 | 60S ribosomal protein L7 OS=Homo sapiens OX=9606 GN=RPL7 PE=1 SV=1 | 3.226 | 1 | 248 | 29.207 | 10.65 |
| P62424 | RPL7A | 60S ribosomal protein L7a OS=Homo sapiens OX=9606 GN=RPL7A PE=1 SV=2 | 6.015 | 2 | 266 | 29.977 | 10.61 |
| P62917 | RPL8 | 60S ribosomal protein L8 OS=Homo sapiens OX=9606 GN=RPL8 PE=1 SV=2 | 9.728 | 3 | 257 | 28.007 | 11.03 |
| O15143 | ARPC1B | Actin-related protein 2/3 complex subunit 1B OS=Homo sapiens OX=9606 GN=ARPC1B PE=1 SV=3 | 3.495 | 1 | 372 | 40.923 | 8.35 |
| Q01518 | CAP1 | Adenylyl cyclase-associated protein 1 OS=Homo sapiens OX=9606 GN=CAP1 PE=1 SV=5 | 5.895 | 3 | 475 | 51.869 | 8.06 |
| P05141 | SLC25A5 | ADP/ATP translocase 2 OS=Homo sapiens OX=9606 GN=SLC25A5 PE=1 SV=7 | 3.020 | 1 | 298 | 32.831 | 9.69 |
| C9JRZ8 | AKR1B15 | Aldo-keto reductase family 1 member B15 OS=Homo sapiens OX=9606 GN=AKR1B15 PE=1 SV=2 | 2.532 | 1 | 316 | 36.514 | 6.7 |
| P12814 | ACTN1 | Alpha-actinin-1 OS=Homo sapiens OX=9606 GN=ACTN1 PE=1 SV=2 | 1.233 | 1 | 892 | 102.993 | 5.41 |
| P04083 | ANXA1 | Annexin A1 OS=Homo sapiens OX=9606 GN=ANXA1 PE=1 SV=2 | 5.491 | 2 | 346 | 38.69 | 7.02 |
| P07355 | ANXA2 | Annexin A2 OS=Homo sapiens OX=9606 GN=ANXA2 PE=1 SV=2 | 18.289 | 8 | 339 | 38.58 | 7.75 |
| P08758 | ANXA5 | Annexin A5 OS=Homo sapiens OX=9606 GN=ANXA5 PE=1 SV=2 | 2.813 | 1 | 320 | 35.914 | 5.05 |
| Q96CW1 | AP2M1 | AP-2 complex subunit mu OS=Homo sapiens OX=9606 GN=AP2M1 PE=1 SV=2 | 2.069 | 1 | 435 | 49.623 | 9.54 |
| P06576 | ATP5F1B | ATP synthase subunit beta, mitochondrial OS=Homo sapiens OX=9606 GN=ATP5F1B PE=1 SV=3 | 2.268 | 1 | 529 | 56.525 | 5.4 |
| Q68CP9 | ARID2 | AT-rich interactive domain-containing protein 2 OS=Homo sapiens OX=9606 GN=ARID2 PE=1 SV=2 | 1.417 | 2 | 1835 | 197.268 | 7.42 |
| P42025 | ACTR1B | Beta-centractin OS=Homo sapiens OX=9606 GN=ACTR1B PE=1 SV=1 | 2.660 | 1 | 376 | 42.267 | 6.4 |
| P07814 | EPRS | Bifunctional glutamate/proline--tRNA ligase OS=Homo sapiens OX=9606 GN=EPRS PE=1 SV=5 | 0.595 | 1 | 1512 | 170.483 | 7.33 |
| Q9NPI1 | BRD7 | Bromodomain-containing protein 7 OS=Homo sapiens OX=9606 GN=BRD7 PE=1 SV=1 | 1.382 | 1 | 651 | 74.092 | 6.39 |
| P27824 | CANX | Calnexin OS=Homo sapiens OX=9606 GN=CANX PE=1 SV=2 | 1.182 | 1 | 592 | 67.526 | 4.6 |
| Q96DG6 | CMBL | Carboxymethylenebutenolidase homolog OS=Homo sapiens OX=9606 GN=CMBL PE=1 SV=1 | 6.122 | 2 | 245 | 28.03 | 7.18 |
| P68400 | CSNK2A1 | Casein kinase II subunit alpha OS=Homo sapiens OX=9606 GN=CSNK2A1 PE=1 SV=1 | 1.790 | 1 | 391 | 45.115 | 7.74 |
| Q9UBR2 | CTSZ | Cathepsin Z OS=Homo sapiens OX=9606 GN=CTSZ PE=1 SV=1 | 3.300 | 1 | 303 | 33.846 | 7.11 |
| Q6NZI2 | CAVIN1 | Caveolae-associated protein 1 OS=Homo sapiens OX=9606 GN=CAVIN1 PE=1 SV=1 | 2.821 | 1 | 390 | 43.45 | 5.6 |
| Q03135 | CAV1 | Caveolin-1 OS=Homo sapiens OX=9606 GN=CAV1 PE=1 SV=4 | 4.494 | 1 | 178 | 20.458 | 6.02 |
| O00299 | CLIC1 | Chloride intracellular channel protein 1 OS=Homo sapiens OX=9606 GN=CLIC1 PE=1 SV=4 | 4.979 | 1 | 241 | 26.906 | 5.17 |
| P23528 | CFL1 | Cofilin-1 OS=Homo sapiens OX=9606 GN=CFL1 PE=1 SV=3 | 7.229 | 1 | 166 | 18.491 | 8.09 |
| Q9NQ92 | COPRS | Coordinator of PRMT5 and differentiation stimulator OS=Homo sapiens OX=9606 GN=COPRS PE=1 SV=3 | 6.522 | 1 | 184 | 20.054 | 4.18 |
| P06493 | CDK1 | Cyclin-dependent kinase 1 OS=Homo sapiens OX=9606 GN=CDK1 PE=1 SV=3 | 5.387 | 1 | 297 | 34.074 | 8.4 |
| P31930 | UQCRC1 | Cytochrome b-c1 complex subunit 1, mitochondrial OS=Homo sapiens OX=9606 GN=UQCRC1 PE=1 SV=3 | 3.958 | 2 | 480 | 52.612 | 6.37 |
| P21399 | ACO1 | Cytoplasmic aconitate hydratase OS=Homo sapiens OX=9606 GN=ACO1 PE=1 SV=3 | 0.900 | 1 | 889 | 98.337 | 6.68 |
| Q07065 | CKAP4 | Cytoskeleton-associated protein 4 OS=Homo sapiens OX=9606 GN=CKAP4 PE=1 SV=2 | 6.478 | 3 | 602 | 65.983 | 5.92 |
| P49902 | NT5C2 | Cytosolic purine 5'-nucleotidase OS=Homo sapiens OX=9606 GN=NT5C2 PE=1 SV=1 | 2.139 | 1 | 561 | 64.928 | 6.14 |
| Q8N1I0 | DOCK4 | Dedicator of cytokinesis protein 4 OS=Homo sapiens OX=9606 GN=DOCK4 PE=1 SV=3 | 0.661 | 1 | 1966 | 225.063 | 7.65 |
| P81605 | DCD | Dermcidin OS=Homo sapiens OX=9606 GN=DCD PE=1 SV=2 | 10.000 | 1 | 110 | 11.277 | 6.54 |
| Q9UHL4 | DPP7 | Dipeptidyl peptidase 2 OS=Homo sapiens OX=9606 GN=DPP7 PE=1 SV=3 | 2.236 | 1 | 492 | 54.307 | 6.32 |
| Q86TI2 | DPP9 | Dipeptidyl peptidase 9 OS=Homo sapiens OX=9606 GN=DPP9 PE=1 SV=3 | 1.275 | 1 | 863 | 98.201 | 6.46 |
| P25205 | MCM3 | DNA replication licensing factor MCM3 OS=Homo sapiens OX=9606 GN=MCM3 PE=1 SV=3 | 0.990 | 1 | 808 | 90.924 | 5.77 |
| Q8IXB1 | DNAJC10 | DnaJ homolog subfamily C member 10 OS=Homo sapiens OX=9606 GN=DNAJC10 PE=1 SV=2 | 1.261 | 1 | 793 | 91.021 | 7.18 |
| Q13561 | DCTN2 | Dynactin subunit 2 OS=Homo sapiens OX=9606 GN=DCTN2 PE=1 SV=4 | 2.244 | 1 | 401 | 44.204 | 5.21 |
| P49792 | RANBP2 | E3 SUMO-protein ligase RanBP2 OS=Homo sapiens OX=9606 GN=RANBP2 PE=1 SV=2 | 0.496 | 1 | 3224 | 357.974 | 6.2 |
| Q15717 | ELAVL1 | ELAV-like protein 1 OS=Homo sapiens OX=9606 GN=ELAVL1 PE=1 SV=2 | 3.374 | 1 | 326 | 36.069 | 9.17 |
| P68104 | EEF1A1 | Elongation factor 1-alpha 1 OS=Homo sapiens OX=9606 GN=EEF1A1 PE=1 SV=1 | 13.203 | 7 | 462 | 50.109 | 9.01 |
| P24534 | EEF1B2 | Elongation factor 1-beta OS=Homo sapiens OX=9606 GN=EEF1B2 PE=1 SV=3 | 6.222 | 1 | 225 | 24.748 | 4.67 |
| P29692 | EEF1D | Elongation factor 1-delta OS=Homo sapiens OX=9606 GN=EEF1D PE=1 SV=5 | 5.694 | 1 | 281 | 31.103 | 5.01 |
| P26641 | EEF1G | Elongation factor 1-gamma OS=Homo sapiens OX=9606 GN=EEF1G PE=1 SV=3 | 2.975 | 1 | 437 | 50.087 | 6.67 |
| P13639 | EEF2 | Elongation factor 2 OS=Homo sapiens OX=9606 GN=EEF2 PE=1 SV=4 | 1.981 | 2 | 858 | 95.277 | 6.83 |
| P49411 | TUFM | Elongation factor Tu, mitochondrial OS=Homo sapiens OX=9606 GN=TUFM PE=1 SV=2 | 6.858 | 3 | 452 | 49.51 | 7.61 |
| P84090 | ERH | Enhancer of rudimentary homolog OS=Homo sapiens OX=9606 GN=ERH PE=1 SV=1 | 10.577 | 1 | 104 | 12.251 | 5.92 |
| P20042 | EIF2S2 | Eukaryotic translation initiation factor 2 subunit 2 OS=Homo sapiens OX=9606 GN=EIF2S2 PE=1 SV=2 | 2.703 | 1 | 333 | 38.364 | 5.8 |
| P60228 | EIF3E | Eukaryotic translation initiation factor 3 subunit E OS=Homo sapiens OX=9606 GN=EIF3E PE=1 SV=1 | 1.798 | 1 | 445 | 52.187 | 6.04 |
| O00303 | EIF3F | Eukaryotic translation initiation factor 3 subunit F OS=Homo sapiens OX=9606 GN=EIF3F PE=1 SV=1 | 2.521 | 1 | 357 | 37.54 | 5.45 |
| Q9Y262 | EIF3L | Eukaryotic translation initiation factor 3 subunit L OS=Homo sapiens OX=9606 GN=EIF3L PE=1 SV=1 | 1.418 | 1 | 564 | 66.684 | 6.34 |
| Q15056 | EIF4H | Eukaryotic translation initiation factor 4H OS=Homo sapiens OX=9606 GN=EIF4H PE=1 SV=5 | 2.823 | 1 | 248 | 27.368 | 7.23 |
| P56537 | EIF6 | Eukaryotic translation initiation factor 6 OS=Homo sapiens OX=9606 GN=EIF6 PE=1 SV=1 | 4.082 | 1 | 245 | 26.582 | 4.68 |
| P15311 | EZR | Ezrin OS=Homo sapiens OX=9606 GN=EZR PE=1 SV=4 | 5.631 | 3 | 586 | 69.37 | 6.27 |
| P49327 | FASN | Fatty acid synthase OS=Homo sapiens OX=9606 GN=FASN PE=1 SV=3 | 0.398 | 1 | 2511 | 273.254 | 6.44 |
| P09382 | LGALS1 | Galectin-1 OS=Homo sapiens OX=9606 GN=LGALS1 PE=1 SV=2 | 5.926 | 1 | 135 | 14.706 | 5.5 |
| O94925 | GLS | Glutaminase kidney isoform, mitochondrial OS=Homo sapiens OX=9606 GN=GLS PE=1 SV=1 | 1.196 | 1 | 669 | 73.414 | 7.77 |
| P78417 | GSTO1 | Glutathione S-transferase omega-1 OS=Homo sapiens OX=9606 GN=GSTO1 PE=1 SV=2 | 7.884 | 2 | 241 | 27.548 | 6.6 |
| P11216 | PYGB | Glycogen phosphorylase, brain form OS=Homo sapiens OX=9606 GN=PYGB PE=1 SV=5 | 1.186 | 1 | 843 | 96.635 | 6.86 |
| P62826 | RAN | GTP-binding nuclear protein Ran OS=Homo sapiens OX=9606 GN=RAN PE=1 SV=3 | 9.722 | 2 | 216 | 24.408 | 7.49 |
| P16520 | GNB3 | Guanine nucleotide-binding protein G(I)/G(S)/G(T) subunit beta-3 OS=Homo sapiens OX=9606 GN=GNB3 PE=1 SV=1 | 2.941 | 1 | 340 | 37.197 | 5.67 |
| P0DMV9 | HSPA1B | Heat shock 70 kDa protein 1B OS=Homo sapiens OX=9606 GN=HSPA1B PE=1 SV=1 | 6.396 | 1 | 641 | 70.009 | 5.66 |
| Q92598 | HSPH1 | Heat shock protein 105 kDa OS=Homo sapiens OX=9606 GN=HSPH1 PE=1 SV=1 | 1.049 | 1 | 858 | 96.804 | 5.39 |
| P04792 | HSPB1 | Heat shock protein beta-1 OS=Homo sapiens OX=9606 GN=HSPB1 PE=1 SV=2 | 4.878 | 1 | 205 | 22.768 | 6.4 |
| P02008 | HBZ | Hemoglobin subunit zeta OS=Homo sapiens OX=9606 GN=HBZ PE=1 SV=2 | 4.930 | 1 | 142 | 15.627 | 8.21 |
| P51858 | HDGF | Hepatoma-derived growth factor OS=Homo sapiens OX=9606 GN=HDGF PE=1 SV=1 | 4.583 | 1 | 240 | 26.772 | 4.73 |
| Q5SSJ5 | HP1BP3 | Heterochromatin protein 1-binding protein 3 OS=Homo sapiens OX=9606 GN=HP1BP3 PE=1 SV=1 | 1.627 | 1 | 553 | 61.169 | 9.67 |
| Q13151 | HNRNPA0 | Heterogeneous nuclear ribonucleoprotein A0 OS=Homo sapiens OX=9606 GN=HNRNPA0 PE=1 SV=1 | 4.262 | 1 | 305 | 30.822 | 9.29 |
| Q14103 | HNRNPD | Heterogeneous nuclear ribonucleoprotein D0 OS=Homo sapiens OX=9606 GN=HNRNPD PE=1 SV=1 | 5.070 | 1 | 355 | 38.41 | 7.81 |
| O60506 | SYNCRIP | Heterogeneous nuclear ribonucleoprotein Q OS=Homo sapiens OX=9606 GN=SYNCRIP PE=1 SV=2 | 3.050 | 1 | 623 | 69.56 | 8.59 |
| O43390 | HNRNPR | Heterogeneous nuclear ribonucleoprotein R OS=Homo sapiens OX=9606 GN=HNRNPR PE=1 SV=1 | 3.791 | 1 | 633 | 70.899 | 8.13 |
| P19367 | HK1 | Hexokinase-1 OS=Homo sapiens OX=9606 GN=HK1 PE=1 SV=3 | 0.981 | 1 | 917 | 102.42 | 6.8 |
| P10412 | HIST1H1E | Histone H1.4 OS=Homo sapiens OX=9606 GN=HIST1H1E PE=1 SV=2 | 18.721 | 4 | 219 | 21.852 | 11.03 |
| Q92522 | H1FX | Histone H1x OS=Homo sapiens OX=9606 GN=H1FX PE=1 SV=1 | 4.225 | 1 | 213 | 22.474 | 10.76 |
| O60814 | HIST1H2BK | Histone H2B type 1-K OS=Homo sapiens OX=9606 GN=HIST1H2BK PE=1 SV=3 | 28.571 | 4 | 126 | 13.882 | 10.32 |
| P68431 | HIST1H3A | Histone H3.1 OS=Homo sapiens OX=9606 GN=HIST1H3A PE=1 SV=2 | 5.147 | 1 | 136 | 15.394 | 11.12 |
| P62805 | HIST1H4A | Histone H4 OS=Homo sapiens OX=9606 GN=HIST1H4A PE=1 SV=2 | 21.359 | 2 | 103 | 11.36 | 11.36 |
| Q86YZ3 | HRNR | Hornerin OS=Homo sapiens OX=9606 GN=HRNR PE=1 SV=2 | 1.930 | 1 | 2850 | 282.228 | 10.04 |
| Q6YN16 | HSDL2 | Hydroxysteroid dehydrogenase-like protein 2 OS=Homo sapiens OX=9606 GN=HSDL2 PE=1 SV=1 | 2.153 | 1 | 418 | 45.366 | 7.99 |
| A0A075B6S2 | IGKV2D-29 | Immunoglobulin kappa variable 2D-29 OS=Homo sapiens OX=9606 GN=IGKV2D-29 PE=3 SV=1 | 16.667 | 2 | 120 | 13.135 | 7.12 |
| O00505 | KPNA3 | Importin subunit alpha-4 OS=Homo sapiens OX=9606 GN=KPNA3 PE=1 SV=2 | 1.919 | 1 | 521 | 57.775 | 4.94 |
| Q14974 | KPNB1 | Importin subunit beta-1 OS=Homo sapiens OX=9606 GN=KPNB1 PE=1 SV=2 | 1.370 | 1 | 876 | 97.108 | 4.78 |
| O00425 | IGF2BP3 | Insulin-like growth factor 2 mRNA-binding protein 3 OS=Homo sapiens OX=9606 GN=IGF2BP3 PE=1 SV=2 | 3.454 | 2 | 579 | 63.666 | 8.87 |
| P42167 | TMPO | Lamina-associated polypeptide 2, isoforms beta/gamma OS=Homo sapiens OX=9606 GN=TMPO PE=1 SV=2 | 1.762 | 1 | 454 | 50.639 | 9.38 |
| Q03252 | LMNB2 | Lamin-B2 OS=Homo sapiens OX=9606 GN=LMNB2 PE=1 SV=4 | 3.710 | 1 | 620 | 69.906 | 5.59 |
| P07195 | LDHB | L-lactate dehydrogenase B chain OS=Homo sapiens OX=9606 GN=LDHB PE=1 SV=2 | 13.772 | 4 | 334 | 36.615 | 6.05 |
| P14174 | MIF | Macrophage migration inhibitory factor OS=Homo sapiens OX=9606 GN=MIF PE=1 SV=4 | 6.087 | 1 | 115 | 12.468 | 7.88 |
| P40926 | MDH2 | Malate dehydrogenase, mitochondrial OS=Homo sapiens OX=9606 GN=MDH2 PE=1 SV=3 | 5.621 | 2 | 338 | 35.481 | 8.68 |
| Q9UNF1 | MAGED2 | Melanoma-associated antigen D2 OS=Homo sapiens OX=9606 GN=MAGED2 PE=1 SV=2 | 1.650 | 1 | 606 | 64.914 | 9.32 |
| O94776 | MTA2 | Metastasis-associated protein MTA2 OS=Homo sapiens OX=9606 GN=MTA2 PE=1 SV=1 | 1.497 | 1 | 668 | 74.976 | 9.66 |
| Q9NX63 | CHCHD3 | MICOS complex subunit MIC19 OS=Homo sapiens OX=9606 GN=CHCHD3 PE=1 SV=1 | 3.965 | 1 | 227 | 26.136 | 8.28 |
| P46821 | MAP1B | Microtubule-associated protein 1B OS=Homo sapiens OX=9606 GN=MAP1B PE=1 SV=2 | 0.851 | 2 | 2468 | 270.468 | 4.81 |
| Q9H936 | SLC25A22 | Mitochondrial glutamate carrier 1 OS=Homo sapiens OX=9606 GN=SLC25A22 PE=1 SV=1 | 2.477 | 1 | 323 | 34.448 | 9.29 |
| Q70IA6 | MOB2 | MOB kinase activator 2 OS=Homo sapiens OX=9606 GN=MOB2 PE=1 SV=1 | 2.954 | 1 | 237 | 26.909 | 6.79 |
| Q6UB35 | MTHFD1L | Monofunctional C1-tetrahydrofolate synthase, mitochondrial OS=Homo sapiens OX=9606 GN=MTHFD1L PE=1 SV=1 | 1.125 | 1 | 978 | 105.724 | 8.06 |
| P60660 | MYL6 | Myosin light polypeptide 6 OS=Homo sapiens OX=9606 GN=MYL6 PE=1 SV=2 | 14.570 | 2 | 151 | 16.919 | 4.65 |
| P13533 | MYH6 | Myosin-6 OS=Homo sapiens OX=9606 GN=MYH6 PE=1 SV=5 | 0.361 | 1 | 1939 | 223.596 | 5.73 |
| Q8NF91 | SYNE1 | Nesprin-1 OS=Homo sapiens OX=9606 GN=SYNE1 PE=1 SV=4 | 0.171 | 1 | 8797 | 1010.456 | 5.53 |
| P67809 | YBX1 | Nuclease-sensitive element-binding protein 1 OS=Homo sapiens OX=9606 GN=YBX1 PE=1 SV=3 | 8.333 | 2 | 324 | 35.903 | 9.88 |
| P06748 | NPM1 | Nucleophosmin OS=Homo sapiens OX=9606 GN=NPM1 PE=1 SV=2 | 10.544 | 4 | 294 | 32.555 | 4.78 |
| P62937 | PPIA | Peptidyl-prolyl cis-trans isomerase A OS=Homo sapiens OX=9606 GN=PPIA PE=1 SV=2 | 7.273 | 1 | 165 | 18.001 | 7.81 |
| Q8WUB8 | PHF10 | PHD finger protein 10 OS=Homo sapiens OX=9606 GN=PHF10 PE=1 SV=3 | 2.008 | 1 | 498 | 56.015 | 6.62 |
| Q00325 | SLC25A3 | Phosphate carrier protein, mitochondrial OS=Homo sapiens OX=9606 GN=SLC25A3 PE=1 SV=2 | 3.315 | 1 | 362 | 40.069 | 9.38 |
| P00558 | PGK1 | Phosphoglycerate kinase 1 OS=Homo sapiens OX=9606 GN=PGK1 PE=1 SV=3 | 1.679 | 1 | 417 | 44.586 | 8.1 |
| Q8NC51 | SERBP1 | Plasminogen activator inhibitor 1 RNA-binding protein OS=Homo sapiens OX=9606 GN=SERBP1 PE=1 SV=2 | 9.804 | 3 | 408 | 44.938 | 8.65 |
| Q15365 | PCBP1 | Poly(rC)-binding protein 1 OS=Homo sapiens OX=9606 GN=PCBP1 PE=1 SV=2 | 8.708 | 3 | 356 | 37.474 | 7.09 |
| P11940 | PABPC1 | Polyadenylate-binding protein 1 OS=Homo sapiens OX=9606 GN=PABPC1 PE=1 SV=2 | 2.830 | 2 | 636 | 70.626 | 9.5 |
| O43900 | PRICKLE3 | Prickle planar cell polarity protein 3 OS=Homo sapiens OX=9606 GN=PRICKLE3 PE=1 SV=2 | 3.252 | 1 | 615 | 68.565 | 7.88 |
| Q92841 | DDX17 | Probable ATP-dependent RNA helicase DDX17 OS=Homo sapiens OX=9606 GN=DDX17 PE=1 SV=2 | 3.567 | 2 | 729 | 80.222 | 8.27 |
| Q9UQ80 | PA2G4 | Proliferation-associated protein 2G4 OS=Homo sapiens OX=9606 GN=PA2G4 PE=1 SV=3 | 9.137 | 3 | 394 | 43.759 | 6.55 |
| Q16186 | ADRM1 | Proteasomal ubiquitin receptor ADRM1 OS=Homo sapiens OX=9606 GN=ADRM1 PE=1 SV=2 | 3.931 | 1 | 407 | 42.127 | 5.07 |
| P25786 | PSMA1 | Proteasome subunit alpha type-1 OS=Homo sapiens OX=9606 GN=PSMA1 PE=1 SV=1 | 3.042 | 1 | 263 | 29.537 | 6.61 |
| P25789 | PSMA4 | Proteasome subunit alpha type-4 OS=Homo sapiens OX=9606 GN=PSMA4 PE=1 SV=1 | 10.728 | 3 | 261 | 29.465 | 7.72 |
| O00622 | CYR61 | Protein CYR61 OS=Homo sapiens OX=9606 GN=CYR61 PE=1 SV=1 | 2.362 | 1 | 381 | 41.998 | 8.21 |
| Q15084 | PDIA6 | Protein disulfide-isomerase A6 OS=Homo sapiens OX=9606 GN=PDIA6 PE=1 SV=1 | 7.045 | 3 | 440 | 48.091 | 5.08 |
| P07237 | P4HB | Protein disulfide-isomerase OS=Homo sapiens OX=9606 GN=P4HB PE=1 SV=3 | 2.362 | 1 | 508 | 57.081 | 4.87 |
| Q86UE4 | MTDH | Protein LYRIC OS=Homo sapiens OX=9606 GN=MTDH PE=1 SV=2 | 1.890 | 1 | 582 | 63.799 | 9.32 |
| P60468 | SEC61B | Protein transport protein Sec61 subunit beta OS=Homo sapiens OX=9606 GN=SEC61B PE=1 SV=2 | 15.625 | 1 | 96 | 9.968 | 11.56 |
| Q9ULW8 | PADI3 | Protein-arginine deiminase type-3 OS=Homo sapiens OX=9606 GN=PADI3 PE=1 SV=2 | 3.012 | 2 | 664 | 74.695 | 5.54 |
| P21980 | TGM2 | Protein-glutamine gamma-glutamyltransferase 2 OS=Homo sapiens OX=9606 GN=TGM2 PE=1 SV=2 | 1.164 | 1 | 687 | 77.28 | 5.22 |
| P35241 | RDX | Radixin OS=Homo sapiens OX=9606 GN=RDX PE=1 SV=1 | 2.744 | 1 | 583 | 68.521 | 6.37 |
| Q9UN86 | G3BP2 | Ras GTPase-activating protein-binding protein 2 OS=Homo sapiens OX=9606 GN=G3BP2 PE=1 SV=2 | 2.697 | 1 | 482 | 54.088 | 5.55 |
| Q9UL25 | RAB21 | Ras-related protein Rab-21 OS=Homo sapiens OX=9606 GN=RAB21 PE=1 SV=3 | 6.667 | 1 | 225 | 24.332 | 7.94 |
| Q15293 | RCN1 | Reticulocalbin-1 OS=Homo sapiens OX=9606 GN=RCN1 PE=1 SV=1 | 2.417 | 1 | 331 | 38.866 | 5 |
| O94788 | ALDH1A2 | Retinal dehydrogenase 2 OS=Homo sapiens OX=9606 GN=ALDH1A2 PE=1 SV=3 | 1.544 | 1 | 518 | 56.688 | 6.05 |
| Q8NFJ5 | GPRC5A | Retinoic acid-induced protein 3 OS=Homo sapiens OX=9606 GN=GPRC5A PE=1 SV=2 | 2.241 | 1 | 357 | 40.225 | 8.15 |
| Q9P2E9 | RRBP1 | Ribosome-binding protein 1 OS=Homo sapiens OX=9606 GN=RRBP1 PE=1 SV=5 | 0.638 | 1 | 1410 | 152.365 | 8.6 |
| O15541 | RNF113A | RING finger protein 113A OS=Homo sapiens OX=9606 GN=RNF113A PE=1 SV=1 | 2.332 | 1 | 343 | 38.763 | 5.69 |
| Q5W0B1 | RNF219 | RING finger protein 219 OS=Homo sapiens OX=9606 GN=RNF219 PE=1 SV=1 | 1.240 | 1 | 726 | 81.066 | 5.72 |
| P38159 | RBMX | RNA-binding motif protein, X chromosome OS=Homo sapiens OX=9606 GN=RBMX PE=1 SV=3 | 7.417 | 3 | 391 | 42.306 | 10.05 |
| P98175 | RBM10 | RNA-binding protein 10 OS=Homo sapiens OX=9606 GN=RBM10 PE=1 SV=3 | 2.366 | 2 | 930 | 103.469 | 5.97 |
| Q9UKM9 | RALY | RNA-binding protein Raly OS=Homo sapiens OX=9606 GN=RALY PE=1 SV=1 | 2.288 | 1 | 306 | 32.444 | 9.17 |
| Q9Y265 | RUVBL1 | RuvB-like 1 OS=Homo sapiens OX=9606 GN=RUVBL1 PE=1 SV=1 | 2.412 | 1 | 456 | 50.196 | 6.42 |
| Q15019 | SEPT2 | Septin-2 OS=Homo sapiens OX=9606 GN=SEPT2 PE=1 SV=1 | 4.432 | 2 | 361 | 41.461 | 6.6 |
| Q16181 | SEPT7 | Septin-7 OS=Homo sapiens OX=9606 GN=SEPT7 PE=1 SV=2 | 2.059 | 1 | 437 | 50.648 | 8.63 |
| P34897 | SHMT2 | Serine hydroxymethyltransferase, mitochondrial OS=Homo sapiens OX=9606 GN=SHMT2 PE=1 SV=3 | 1.786 | 1 | 504 | 55.958 | 8.53 |
| Q07955 | SRSF1 | Serine/arginine-rich splicing factor 1 OS=Homo sapiens OX=9606 GN=SRSF1 PE=1 SV=2 | 4.032 | 1 | 248 | 27.728 | 10.36 |
| Q13247 | SRSF6 | Serine/arginine-rich splicing factor 6 OS=Homo sapiens OX=9606 GN=SRSF6 PE=1 SV=2 | 2.616 | 1 | 344 | 39.563 | 11.43 |
| Q15208 | STK38 | Serine/threonine-protein kinase 38 OS=Homo sapiens OX=9606 GN=STK38 PE=1 SV=1 | 4.301 | 2 | 465 | 54.155 | 7.15 |
| P62714 | PPP2CB | Serine/threonine-protein phosphatase 2A catalytic subunit beta isoform OS=Homo sapiens OX=9606 GN=PPP2CB PE=1 SV=1 | 2.589 | 1 | 309 | 35.552 | 5.43 |
| P60510 | PPP4C | Serine/threonine-protein phosphatase 4 catalytic subunit OS=Homo sapiens OX=9606 GN=PPP4C PE=1 SV=1 | 2.606 | 1 | 307 | 35.057 | 5.06 |
| P50454 | SERPINH1 | Serpin H1 OS=Homo sapiens OX=9606 GN=SERPINH1 PE=1 SV=2 | 2.632 | 1 | 418 | 46.411 | 8.69 |
| P42224 | STAT1 | Signal transducer and activator of transcription 1-alpha/beta OS=Homo sapiens OX=9606 GN=STAT1 PE=1 SV=2 | 0.933 | 1 | 750 | 87.28 | 6.05 |
| P14678 | SNRPB | Small nuclear ribonucleoprotein-associated proteins B and B' OS=Homo sapiens OX=9606 GN=SNRPB PE=1 SV=2 | 5.833 | 1 | 240 | 24.594 | 11.19 |
| Q9Y657 | SPIN1 | Spindlin-1 OS=Homo sapiens OX=9606 GN=SPIN1 PE=1 SV=3 | 8.779 | 2 | 262 | 29.582 | 6.96 |
| Q15637 | SF1 | Splicing factor 1 OS=Homo sapiens OX=9606 GN=SF1 PE=1 SV=4 | 1.565 | 1 | 639 | 68.286 | 8.98 |
| P23246 | SFPQ | Splicing factor, proline- and glutamine-rich OS=Homo sapiens OX=9606 GN=SFPQ PE=1 SV=2 | 2.122 | 2 | 707 | 76.102 | 9.44 |
| Q15020 | SART3 | Squamous cell carcinoma antigen recognized by T-cells 3 OS=Homo sapiens OX=9606 GN=SART3 PE=1 SV=1 | 1.765 | 2 | 963 | 109.865 | 5.57 |
| Q9Y6N5 | SQOR | Sulfide:quinone oxidoreductase, mitochondrial OS=Homo sapiens OX=9606 GN=SQOR PE=1 SV=1 | 4.889 | 2 | 450 | 49.929 | 9.11 |
| Q969G3 | SMARCE1 | SWI/SNF-related matrix-associated actin-dependent regulator of chromatin subfamily E member 1 OS=Homo sapiens OX=9606 GN=SMARCE1 PE=1 SV=2 | 2.433 | 1 | 411 | 46.621 | 4.88 |
| Q0IIM8 | TBC1D8B | TBC1 domain family member 8B OS=Homo sapiens OX=9606 GN=TBC1D8B PE=1 SV=2 | 0.625 | 1 | 1120 | 128.627 | 5.95 |
| P17987 | TCP1 | T-complex protein 1 subunit alpha OS=Homo sapiens OX=9606 GN=TCP1 PE=1 SV=1 | 2.338 | 1 | 556 | 60.306 | 6.11 |
| P50991 | CCT4 | T-complex protein 1 subunit delta OS=Homo sapiens OX=9606 GN=CCT4 PE=1 SV=4 | 2.597 | 2 | 539 | 57.888 | 7.83 |
| P48643 | CCT5 | T-complex protein 1 subunit epsilon OS=Homo sapiens OX=9606 GN=CCT5 PE=1 SV=1 | 3.327 | 2 | 541 | 59.633 | 5.66 |
| P10599 | TXN | Thioredoxin OS=Homo sapiens OX=9606 GN=TXN PE=1 SV=3 | 15.238 | 2 | 105 | 11.73 | 4.92 |
| Q86V81 | ALYREF | THO complex subunit 4 OS=Homo sapiens OX=9606 GN=ALYREF PE=1 SV=3 | 3.891 | 1 | 257 | 26.872 | 11.15 |
| O00268 | TAF4 | Transcription initiation factor TFIID subunit 4 OS=Homo sapiens OX=9606 GN=TAF4 PE=1 SV=2 | 1.290 | 1 | 1085 | 110.047 | 9.94 |
| Q96QR8 | PURB | Transcriptional activator protein Pur-beta OS=Homo sapiens OX=9606 GN=PURB PE=1 SV=3 | 4.167 | 1 | 312 | 33.22 | 5.43 |
| P62995 | TRA2B | Transformer-2 protein homolog beta OS=Homo sapiens OX=9606 GN=TRA2B PE=1 SV=1 | 3.472 | 1 | 288 | 33.646 | 11.25 |
| O94759 | TRPM2 | Transient receptor potential cation channel subfamily M member 2 OS=Homo sapiens OX=9606 GN=TRPM2 PE=1 SV=2 | 1.863 | 1 | 1503 | 171.089 | 7.5 |
| P55072 | VCP | Transitional endoplasmic reticulum ATPase OS=Homo sapiens OX=9606 GN=VCP PE=1 SV=4 | 1.241 | 1 | 806 | 89.266 | 5.26 |
| Q9BYE2 | TMPRSS13 | Transmembrane protease serine 13 OS=Homo sapiens OX=9606 GN=TMPRSS13 PE=2 SV=4 | 1.365 | 1 | 586 | 63.113 | 8.63 |
| P55084 | HADHB | Trifunctional enzyme subunit beta, mitochondrial OS=Homo sapiens OX=9606 GN=HADHB PE=1 SV=3 | 2.321 | 1 | 474 | 51.262 | 9.41 |
| P06753 | TPM3 | Tropomyosin alpha-3 chain OS=Homo sapiens OX=9606 GN=TPM3 PE=1 SV=2 | 7.018 | 2 | 285 | 32.93 | 4.72 |
| P35030 | PRSS3 | Trypsin-3 OS=Homo sapiens OX=9606 GN=PRSS3 PE=1 SV=2 | 4.276 | 1 | 304 | 32.508 | 7.49 |
| Q9BVA1 | TUBB2B | Tubulin beta-2B chain OS=Homo sapiens OX=9606 GN=TUBB2B PE=1 SV=1 | 7.416 | 3 | 445 | 49.921 | 4.89 |
| P09012 | SNRPA | U1 small nuclear ribonucleoprotein A OS=Homo sapiens OX=9606 GN=SNRPA PE=1 SV=3 | 2.837 | 1 | 282 | 31.259 | 9.83 |
| Q8WWY3 | PRPF31 | U4/U6 small nuclear ribonucleoprotein Prp31 OS=Homo sapiens OX=9606 GN=PRPF31 PE=1 SV=2 | 6.814 | 4 | 499 | 55.421 | 5.78 |
| P22314 | UBA1 | Ubiquitin-like modifier-activating enzyme 1 OS=Homo sapiens OX=9606 GN=UBA1 PE=1 SV=3 | 0.756 | 1 | 1058 | 117.774 | 5.76 |
| O60701 | UGDH | UDP-glucose 6-dehydrogenase OS=Homo sapiens OX=9606 GN=UGDH PE=1 SV=1 | 2.024 | 1 | 494 | 54.989 | 7.12 |
| O43795 | MYO1B | Unconventional myosin-Ib OS=Homo sapiens OX=9606 GN=MYO1B PE=1 SV=3 | 0.968 | 1 | 1136 | 131.902 | 9.38 |
| O00159 | MYO1C | Unconventional myosin-Ic OS=Homo sapiens OX=9606 GN=MYO1C PE=1 SV=4 | 0.753 | 1 | 1063 | 121.606 | 9.41 |
| P26640 | VARS | Valine--tRNA ligase OS=Homo sapiens OX=9606 GN=VARS PE=1 SV=4 | 0.554 | 1 | 1264 | 140.387 | 7.59 |
| O75083 | WDR1 | WD repeat-containing protein 1 OS=Homo sapiens OX=9606 GN=WDR1 PE=1 SV=4 | 1.485 | 1 | 606 | 66.152 | 6.65 |
| P12956 | XRCC6 | X-ray repair cross-complementing protein 6 OS=Homo sapiens OX=9606 GN=XRCC6 PE=1 SV=2 | 1.642 | 1 | 609 | 69.799 | 6.64 |
| Q5T200 | ZC3H13 | Zinc finger CCCH domain-containing protein 13 OS=Homo sapiens OX=9606 GN=ZC3H13 PE=1 SV=1 | 0.600 | 1 | 1668 | 196.519 | 9.42 |
